# Prevalence of chronic kidney disease stages 3–5 in low- and middle-income countries in Asia: A systematic review and meta-analysis

**DOI:** 10.1371/journal.pone.0264393

**Published:** 2022-02-25

**Authors:** Pongpan Suriyong, Chidchanok Ruengorn, Chairat Shayakul, Puree Anantachoti, Penkarn Kanjanarat

**Affiliations:** 1 Department of Pharmaceutical Care, Faculty of Pharmacy, Chiang Mai University, Chiang Mai, Thailand; 2 Pharmacoepidemiology and Statistics Research Center (PESRC), Faculty of Pharmacy, Chiang Mai University, Chiang Mai, Thailand; 3 Renal Division, Department of Medicine, Faculty of Medicine Siriraj Hospital, Mahidol University, Bangkok, Thailand; 4 Social and Administrative Pharmacy Department, Faculty of Pharmaceutical Sciences, Chulalongkorn University, Bangkok, Thailand; International University of Health and Welfare, School of Medicine, JAPAN

## Abstract

Chronic kidney disease (CKD) is a major public health problem in low- and middle-income countries (LMICs). Although CKD prevalence has been rapidly increasing in LMICs, particularly in Asia, quantitative studies on the current epidemiology of CKD in this region are limited. This study aimed to identify the prevalence of CKD stages 3–5 in LMICs in Asia, by subregion, country economy classification, identification of CKD, traditional and non-traditional risk factors. A systematic review and meta-analysis of observational studies was conducted through a literature search of seven electronic databases and grey literature search published until November 2021. The Newcastle-Ottawa quality assessment scale (NOS) was used to assess the risk of bias of each study. A random-effects model was used to estimate pooled prevalence. The protocol is registered in the International Prospective Register of Systematic Reviews (PROSPERO CRD42019120519). Of 4,548 potentially relevant records, 110 studies with moderate and high quality were included with 4,760,147 subjects. The average prevalence (95% CI) of CKD stages 3–5 in 14 LMICs in Asia was 11.2% (9.3–13.2%). The prevalence of CKD stages 3–5 was varied among subregions and country economic classification. CKD prevalence was 8.6% (7.2–10.2%) in east Asia, 12.0% (7.7–17.0%) in south-east Asia, 13.1% (8.7–18.2%) in western Asia, and 13.5% (9.5–18.0%) in south Asia. CKD prevalence was 9.8% (8.3–11.5%) in upper-middle-income countries and 13.8% (9.9–18.3%) in lower-middle-income countries. Prevalence of CKD stage 3–5 in LMICs in Asia is comparable to global prevalence. High level of heterogeneity was observed. Study of factors and interventions that lead to the delay of CKD progression is needed.

## Introduction

The global prevalence of chronic kidney disease (CKD) was estimated to be 13.4% in all five stages and 10.6% in stages 3–5 in a recent systematic review and meta-analysis [1]. CKD cases have been continuously increasing, with a 7% increase in end-stage renal disease (ESRD) observed worldwide [2]. The mean prevalence of CKD in high-income countries is approximately 8.6% in men and 9.6% in women [3]. In 2010, about 500 million people worldwide were afflicted with CKD, with 80% of those people living in low- and middle-income countries (LMICs) [3, 4]. A lower income has been identified to be a significant factor associated with CKD prevalence [5]. LMICs have undergone rapid urbanisation that has coincided with a growing number of people with chronic diseases such as diabetes and hypertension, which may lead to CKD. In Asia, 41 countries have been classified as LMICs by the World Bank [6, 7]. The prevalence of CKD in Asia varies widely [8]. Although several studies have assessed CKD prevalence in different countries in Asia, there are very few quantitative studies on the current epidemiology of CKD and its risk factors in this region. Therefore, the objectives of this study aimed to examine the prevalence of CKD stages 3–5 in LMICs in Asia.

## Methods

### Data sources and search strategy

The protocol was published in the PROSPERO (CRD42019120519). Observational studies published up to 30 November 2021 were searched from the following seven databases: PubMed/Medline, ScienceDirect, Embase, Scopus, Cochrane Library, Thai Library Integrated System and Thai Thesis Database. A search of ‘grey literature’ was also conducted through OpenSIGLE, conference proceedings and a manual search. Articles published in English or Thai were considered, using the following keywords: ‘prevalence’, ‘chronic kidney disease’ and ‘Asia’.

### Selection criteria

Published studies that specified the prevalence of CKD stages 3–5 as an outcome were included. CKD stages 3–5 was defined as an estimated glomerular filtration rate (eGFR) <60 mL/min/1.73m^2^. Two investigators independently selected relevant studies based on the following inclusion criteria: (1) the observational studies, including cross-sectional study and cohort study; (2) the study participants aged 15 years and above; (3) the study participants included both males and females; (4) included at least 50 participants; (5) reported the prevalence of CKD stage 3–5 and ESRD; (6) the observed participants were non-dialysis-dependent CKD; and (7) CKD estimated by serum creatinine (Scr) or eGFR using the CKD-EPI creatinine equation (CKD-EPI), four-variable Modification of Diet in Renal Disease (MDRD), body surface area (BSA) or standardised Cockcroft-Gault (CG) equations. Studies were excluded based on the following criteria: (1) review articles, (2) not available in full-text version, (3) selected participants based on the absence or presence of kidney disease, (4) studied only in pregnant women, (5) studied only in children and (6) duplicate publications.

### Data extraction

The citations of all searched articles were imported into Endnote X9 citation management software [9]. Two authors, PK and PS, screened the titles and abstracts of each study independently. Duplicate publications were removed. Full review and quality assessment were conducted for all full-text articles by two authors (PS and PK). Then, data were extracted from each study and entered into the standard data collection form [10, 11]. Data extraction was independently performed by the two authors (PS and PK) [10, 11]. Dissimilarities in data extraction were discussed until a consensus was reached. The following data were extracted: title of the article, first author’s surname, authors’ affiliations, publication year, study period, study design, country where the study was conducted, targeted population, sample size, inclusion criteria, exclusion criteria, diagnosis methods for CKD, number of cases, number of controls, percentage of males and females, number of observation CKD cases, and CKD risk factors [10–12]. The prevalence of CKD stages 3–5, odds ratios (OR), relative risk (RR), or the adjusted OR if available were extracted [10–12].

### Quality assessment

Study quality and risk of bias were evaluated by the two authors (PS and PK). The Newcastle-Ottawa quality assessment scale (NOS) was used to assess the bias of each study. If any assessments were inconsistent, these were resolved through discussion to reach a consensus for each study [13–15]. The study characteristics were rated using seven and eight multiple choice questions across three areas [15, 16] including: (1) the selection of study participants (maximum of 4 stars in cohort studies and 5 stars in cross-sectional studies); (2) the comparability of groups by adjusting for first and second most appropriate factors (maximum of 2 stars); and (3) assessment of interested outcome in cohort and cross-sectional studies (maximum of 3 stars) [11, 13, 15, 16]. A total score of <3 was considered low quality, 4–6 was moderate and 7–10 was high quality.

### Publication bias testing

The validity of systematic reviews and meta-analyses on publication bias was tested using an objective judgment funnel plot and Egger’s test [10, 17, 18]. When publication bias was present, the trim-and-fill method was performed to identify and correct it.

### Statistical analysis

Meta-analyses were conducted using Stata version 14 statistical software [19]. Analyses of pooled estimates of the prevalence of CKD stages 3–5 were performed using inverse of the Freeman-Tukey double arcsine transformation for stabilising the variance prior to the estimation [20, 21] and presented as percentages with a 95% confidence interval (95% CI), I^2^, and p-value for heterogeneity. The prevalence of CKD stages 3–5 was presented using forest plots. Heterogeneity was assessed for the statistical significance using Cochran’s Q, I^2^, Tau^2^ and p-value <0.05. If I^2^ ≤50%, pooled prevalence was analysed using a fixed-effects model. If I^2^ >50%, pooled prevalence was analysed using a random-effects model [11, 22, 23].

Furthermore, sensitivity analysis was performed to repeat the random-effects of meta-analysis after the addition of a low-quality study on the pooled estimate of the prevalence of CKD stages 3–5 [17]. Subgroup analysis was performed by geographical subregion in Asia, economic group based on the World Bank, study design, measurement method of eGFR, quality of study, gender, proportion of hypertensive population, and proportion of diabetic population. Meta-regression identified the effect of study design on pooled OR of each risk factor on prevalence of CKD stage 3–5 in LMICs in Asia.[17].

## Results

**Fig 1:**
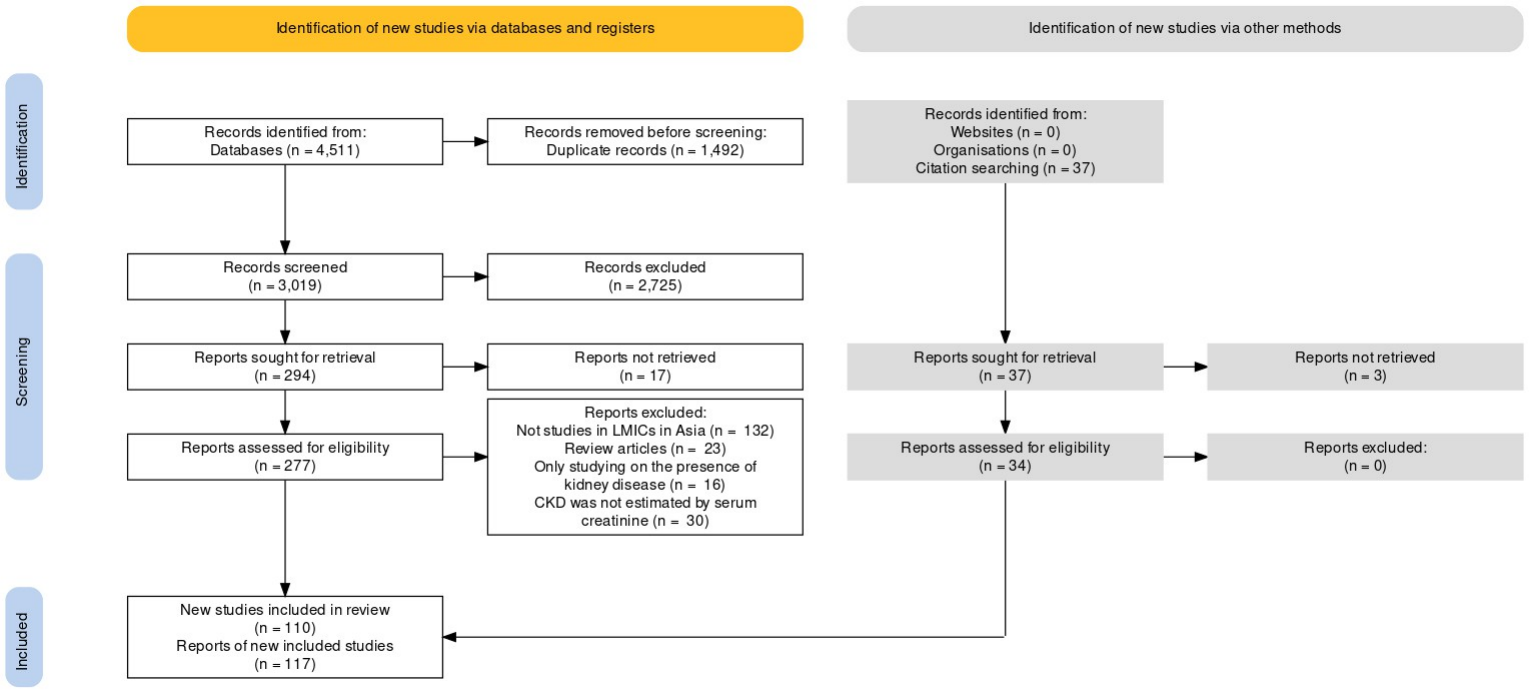
PRISMA flow chart for identifying relevant studies.

The initial search retrieved all 4,511 records from the databases. After screening the titles and abstracts, 2,725 records were excluded as nonrelevant based on the selection criteria. A full-text review of 277 studies was conducted, yielding 76 that met the criteria. In addition, 34 studies from citation search were included. Of these, 110 studies (76 database and 34 citation searching), 117 reports were included to estimate the pooled prevalence of CKD stages 3–5 (Fig 1).

### Characteristics of the included studies

All 110 included studies were published in English and conducted between 2004 and 2021. A total of 4,760,147subjects was included in the meta-analysis of pooled estimate prevalence of CKD stages 3–5. The number of participants ranged from 100 in an Indian cross-sectional study [24] to 3,091,379 in a Chinese cross-sectional study [25]. The study populations’ age ranged from 15 to 95 years. There were 100 cross-sectional studies (61 high- and 39 moderate-quality studies) and 10 cohort studies (three high- and seven moderate-quality studies). China had the largest number of population samples in 47 studies. Most population lived in east Asia and upper-middle income countries. They were classified as CKD patients by the CKD-EPI method for the measurement of eGFR. Most observed studies had low proportions of hypertensive patients and high proportions of diabetes patients in study populations [26, 27] (Table 1). The other characteristics of the included studies are presented in S4 Appendix.

**Table 1:** Characteristic of studies included in the meta-analysis.

Study characteristics	Number of participants	Number of reports (N = 117)	%
**Geographical subregion in Asia**			
East Asia	4,272,462	48	41.0
South Asia	311,067	41	35.1
Southeast Asia	99,159	17	14.5
Western Asia	77,459	11	9.4
**Economic group (classified by the World Bank)**			
Upper-middle income countries	4,415,867	69	59.0
Lower-middle income countries	321,682	45	38.5
Low income countries	22,598	3	2.5
**Study design**			
Cross-sectional study	4,715,186	108	92.3
Cohort study	44,961	9	7.7
**Methods for measurement of eGFR**			
CKD-EPI	4,119,935	46	39.3
MDRD _186_	133,764	36	30.8
eMDRD _175_	442,723	29	24.8
Cockcroft-Gault	457	2	1.7
Not reported	63,268	4	3.4
**Quality of study**			
High-quality study	1,102,079	67	57.3
Moderate-quality study	3,658,068	50	42.7
**Proportions of hypertension patients in study populations** [26]			
≤ 36.9%	3,732,160	58	49.6
> 36.9%	494,622	43	36.7
Not reported	533,365	16	13.7
**Proportions of diabetes patients in study populations** [27]			
≤ 8.1%	3,588,668	32	27.4
> 8.1%	611,899	63	53.8
Not reported	559,580	22	18.8

* estimated prevalence calculated using random-effect models; eGFR, estimated glomerular filtration rate; CKD, chronic kidney disease; EPI, epidemiology collaboration equation; MDRD_186_, Modification of Diet in Renal Disease Study with constant factor of 186; eMDRD_175_, estimated Modification of Diet in Renal Disease Study with constant factor of 175

### Quality assessment and publication bias testing

All 110 studies had a NOS score ≥4, as reported in S4 Appendix. Accordingly, 64 studies were considered high quality and 46 studies were moderate quality. Assessment of publication bias by funnel plot and Egger’s test of the logit prevalence of CKD stages 3–5 in LMICs in Asia showed no small-study effect (p = 0.137).

### Prevalence

**Fig 2:**
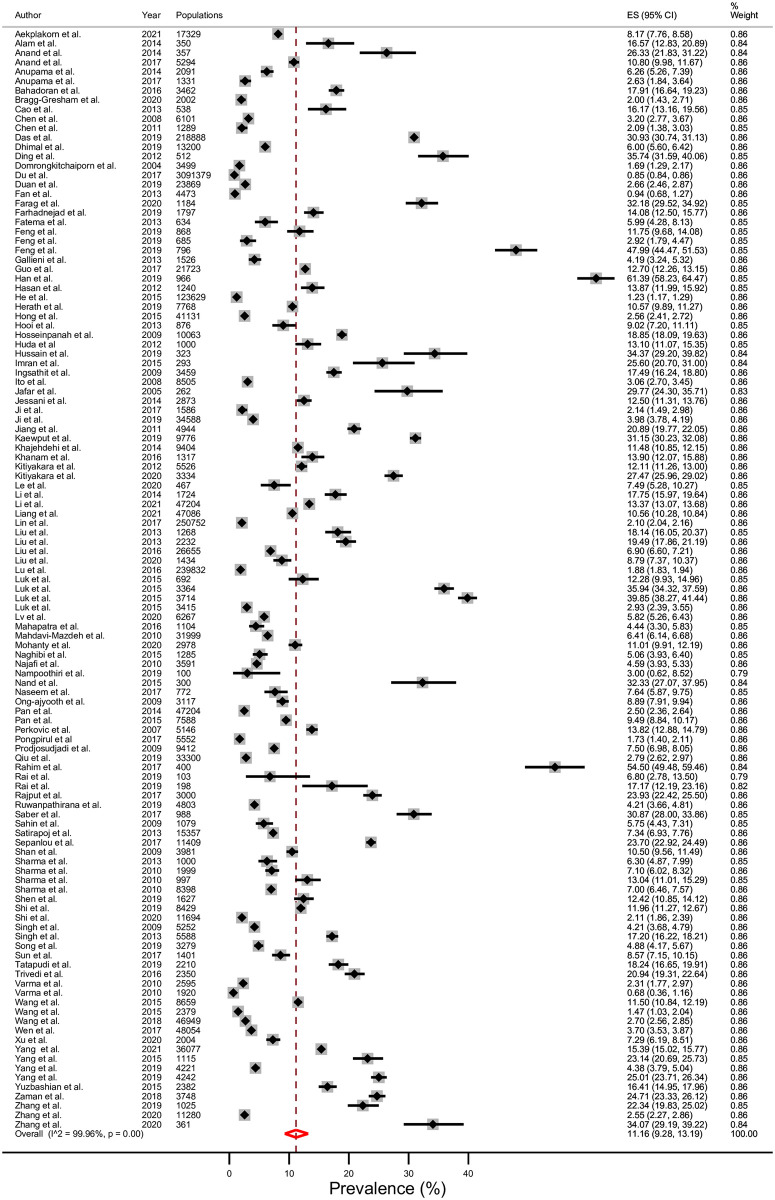
Prevalence of chronic kidney disease (CKD) stages 3–5 in low- and middle- income countries (LMICs) in Asia using a random-effects model.

**Fig 3:**
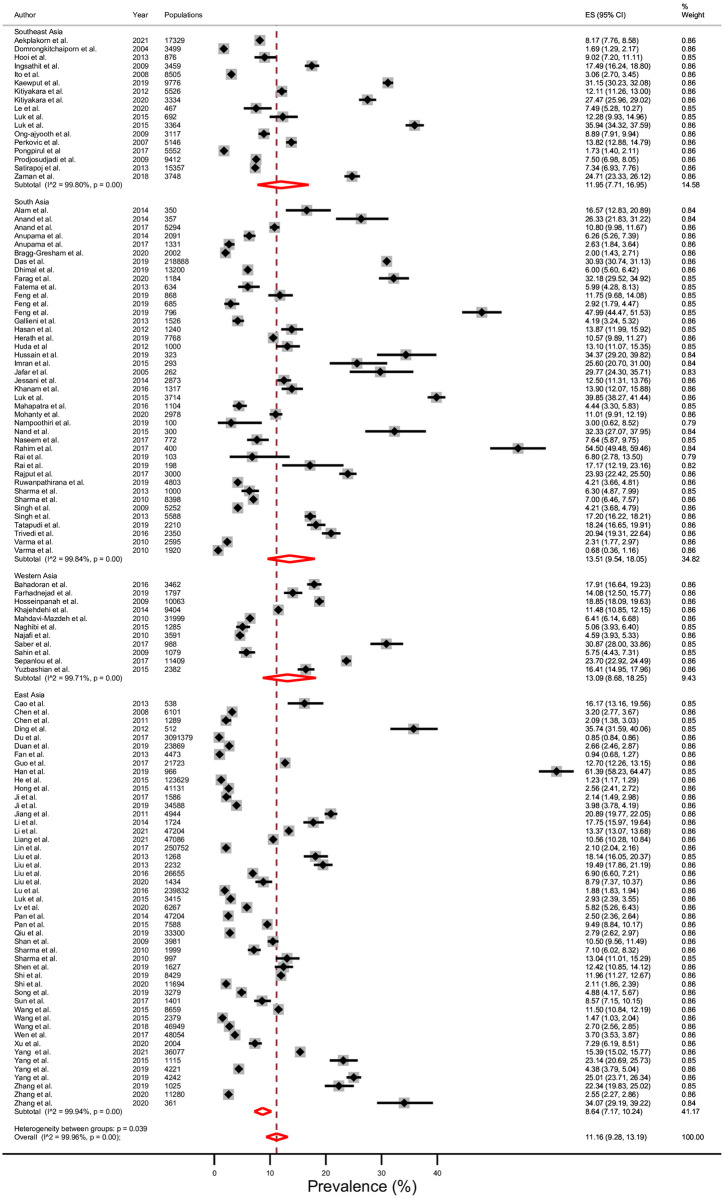
Prevalence of chronic kidney disease (CKD) stages 3–5 in low- and middle- income countries (LMICs) according to subregion of Asia using a random-effects model.

**Fig 4:**
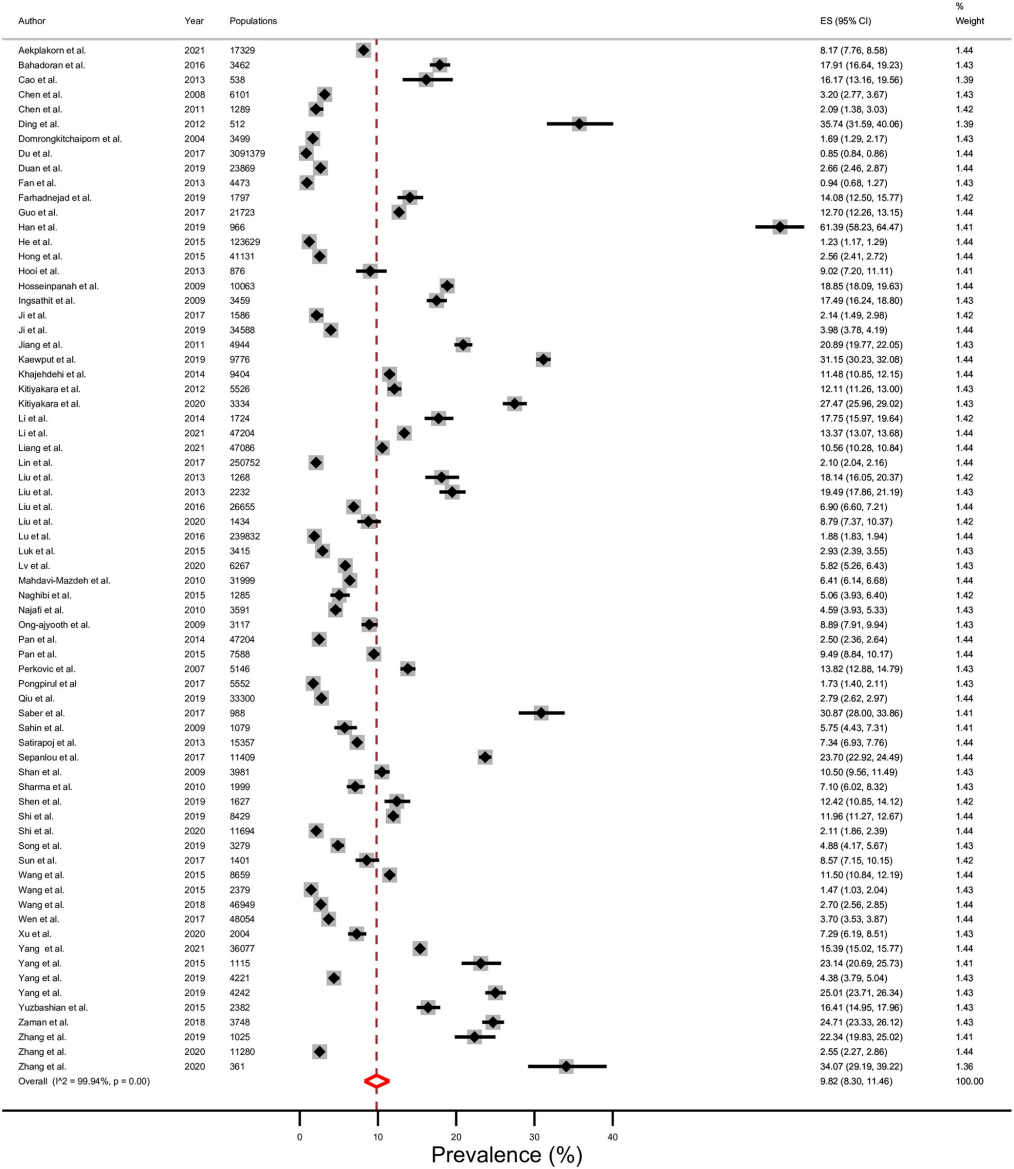
Prevalence of chronic kidney disease (CKD) stages 3–5 in upper-middle income countries using a random-effects model.

**Fig 5:**
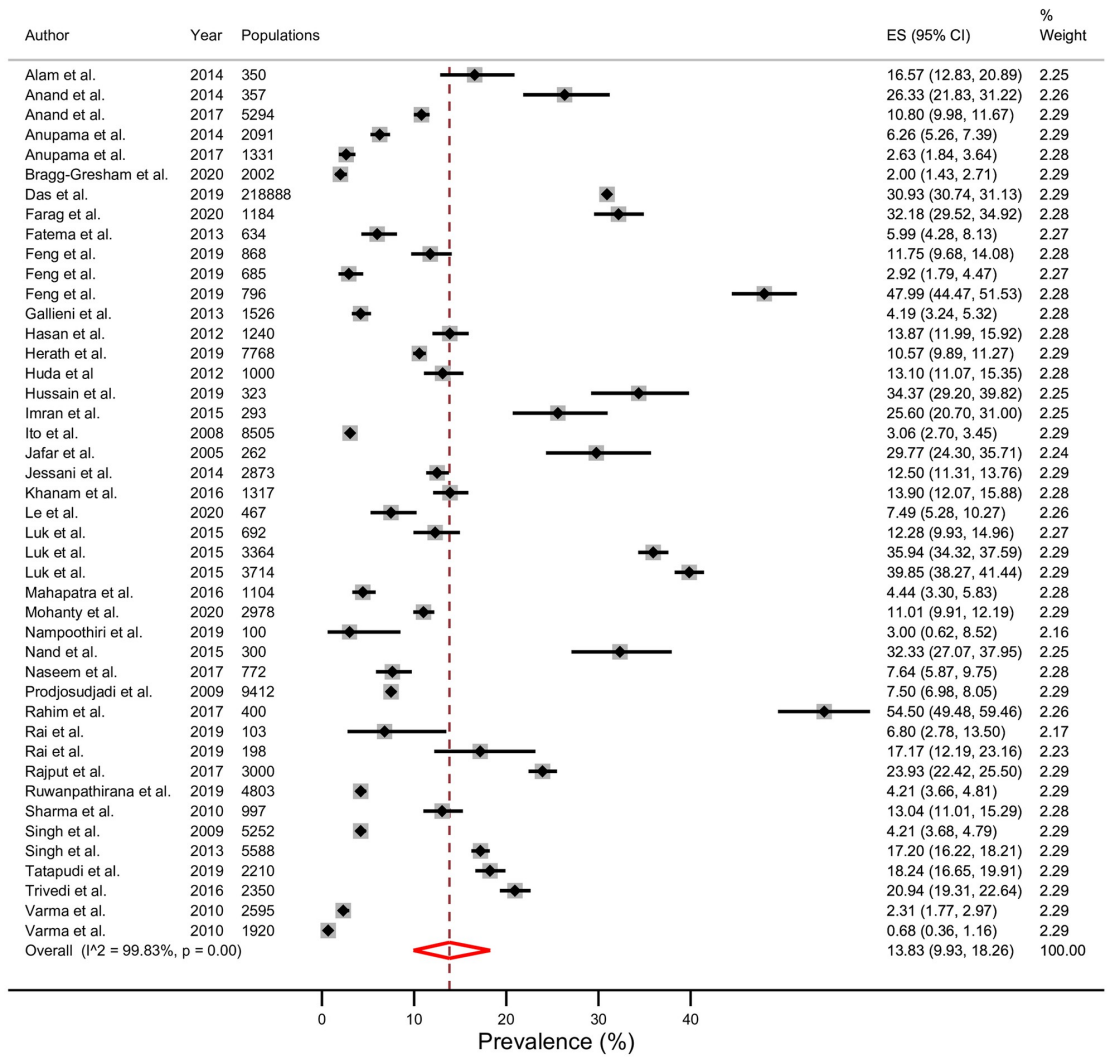
Prevalence of chronic kidney disease (CKD) stages 3–5 in lower-middle income countries using a random-effects model.

The average prevalence of CKD stages 3–5 in 14 LMICs in Asia was 11.2% (95% CI; 9.3–13.2%), Tau^2^ = 0.12, I^2^ = 99.96%, and p <0.001 (Fig 2). The prevalence of CKD stages 3–5 divided by geographical subregion in Asia (Fig 3) was 8.6% (7.2–10.2%) in east Asia, 12.0% (7.7–17.0%) in south-east Asia, 13.1% (8.7–18.2%) in western Asia, and 13.5% (9.5–18.0%) in south Asia. The prevalence of CKD stages 3–5 was 9.8% in upper-middle income countries (Fig 4); 13.8% in lower-middle income countries (Fig 5); and 6.4% in one low-income country. In addition, the rising mean prevalence of CKD stages 3–5 was observed in year 2011–2021 (12.4%) compared with 9.5% in the year before 2011(Table 2). In comparing with the global mean CKD stages 3–5 prevalence in the year 2015 [1] (Table 2), mean CKD prevalence in three countries; Thailand (12.4%), India (11.7%) and Malaysia (9.0%), was comparable to the global mean CKD stages 3–5 prevalence. The mean CKD prevalence in six countries; Philippines (35.9%), Bangladesh (19.8%), Sri Lanka (17.6%), Pakistan (14.3%), Iran (14.0%) and Mongolia (13.0%), was higher than global mean CKD stage 3–5 prevalence [1]. Conversely, mean CKD prevalence in five countries; China (8.6%), Indonesia (7.5%), Vietnam (7.1%), Nepal (6.4%) and Turkey (5.8%), was lower than global mean CKD stages 3–5 prevalence [1].

**Table 2:** Pooled estimate prevalence of chronic kidney disease (CKD) stages 3–5 in low- and middle-income countries (LMICs) by individual countries and in comparison with global mean CKD stages 3–5 prevalence 2015 [1] and study period using a random-effects model.

Countries/ Study period	Number of reports	Total number of populations	Pooled prevalence ^a^ (95% CI^b^)	I^2^	p-value
**Lower than global CKD stage 3–5 prevalence** (<9%)					
Turkey	1	1,079	5.75 (4.43–7.31)	NR	NR
Nepal	3	22,598	6.44 (5.67–7.25)	76.51%	<0.001
Vietnam	3	9,664	7.08 (2.08–14.69)	98.00%	<0.001
Indonesia	1	9,412	7.50 (6.98–8.05)	NR	NR
China	47	4,271,465	8.56 (7.08–10.16)	99.94%	<0.001
**Comparable to global CKD stage 3–5 prevalence** (9–12%)					
Malaysia	1	876	9.02 (7.20–11.11)	NR	NR
India	21	45,163	11.73 (7.36–16.96)	99.60%	<0.001
Thailand	11	75,843	12.42 (7.25–18.73)	99.83%	<0.001
**Higher than global CKD stage 3–5 prevalence** (>12%)					
Mongolia	1	997	13.04 (11.01–15.29)	NR	NR
Iran	10	76,380	13.96 (9.19–19.53)	99.73%	<0.001
Pakistan	6	5,235	14.29 (8.14–21.82)	97.52%	<0.001
Sri Lanka	3	13,367	17.63 (5.35–34.99)	99.77%	<0.001
Bangladesh	8	224,704	19.77 (11.62–29.44)	99.42%	<0.001
Philippines	1	3,364	35.94 (34.32–37.59)	NR	NR
**Study period**					
Year before 2011	49	496,636	9.49 (7.53–11.66)	99.83%	<0.001
Year 2011–2021	68	4,263,511	12.44 (9.70–15.46)	99.97%	<0.001

^a^ Pooled prevalence calculated using random-effect model;

^b^ 95% confidence interval;

NR, not reported;

### Effect of traditional risk factors and non-traditional risk factors on mean prevalence of chronic kidney disease (CKD) stages 3–5 in low- and middle-income countries (LMICs) in Asia

In 69 of 110 studies, the meta-analysis of CKD prevalence and covariates were analyzed. CKD was significantly associated with 10 traditional risk factors: elderly population (OR = 3.79), obese (OR = 1.33), hypertension (OR = 2.55), diabetes (OR = 2.25), hypertriglyceridemia (OR = 1.45), hypercholesterolemia (OR = 1.33), low level of high-density lipoprotein cholesterol (OR = 1.28), history of coronary heart disease (recalculated OR using trim-and-fill analysis for publication bias = 1.99), history of stroke (OR = 4.88) and history of cardiovascular disease (OR = 2.76). Furthermore, CKD was significantly associated with five non-traditional risk factors, including education level (OR = 2.01), hyperuricemia (OR = 2.74), anemia (OR = 2.80), family history of CKD (OR = 2.82) and nonsteroidal anti-inflammatory drugs (NSAIDs) use (OR = 1.97). On the contrary, this meta-analysis found the insignificant associations between CKD stages 3–5 and gender, marital status, lower body weight, dyslipidemia, high level of low-density lipoprotein cholesterol (LDLc), smoking status, alcohol consumption, physical activity, family history of hypertension, and CD4 cell count in HIV patients (Table 3).

**Table 3:** Pooled estimate odd ratio and meta-regression: The effect of traditional risk factors and non-traditional risk factors on prevalence of chronic kidney disease (CKD) stage 3–5 in low- and middle-income countries (LMICs) in Asia.

Risk factors	Number of reports	Total number of populations	Pooled OR^a^ (95% CI^b^)	I^2^, p-value	Univariable meta- regression by study design (p-value)
**Traditional risk factors**					
Elderly (age ≥60 years or <60 years)	12	78,831	3.79 (2.02–7.13)	99.50%, <0.001	0.922
Male	57	3,725,926	0.88 (0.71–1.09)	99.10%,0.234	0.751
Obese (BMI ≥25 or 18–25 kg/m^2^)	29	3,373,809	1.33 (1.15–1.53)	94.60%, <0.001	0.258
Lower weight (BMI <18 or 18–25 kg/m^2^)	16	200,483	1.00 (0.77–1.28)	85.90%, 0.975	0.523
Hypertension (yes or no)	49	3,816,788	2.55 (2.00–3.25)	99.20%, <0.001	0.881
Diabetes (yes or no)	40	3,754,121	2.25 (1.63–3.12)	99.20%, <0.001	0.833
Dyslipidemia (yes or no)	10	111,060	1.91 (0.79–4.62)	99.60%, 0.149	0.607
Hypertriglyceridemia (yes or no)	8	324,667	1.45 (1.24–1.71)	89.0%, <0.001	NR
Hypercholesterolemia (yes or no)	6	315,127	1.33 (1.03–1.72)	88.40%, 0.03	NR
High LDLc (yes or no)	6	346,250	2.06 (0.54–7.93)	99.50%, 0.293	NR
Low HDLc (yes or no)	7	297,740	1.28 (1.06–1.55)	86.8%, 0.009	0.650
History of CHD (yes or no)	10	156,885	1.99 (1.18–3.35)^d^	-, 0.01^d^	0.955
History of stroke (yes or no)	4	78,983	4.88 (2.23–10.69)	88.60%, <0.001	NR
History of CVD (yes or no)	6	124,508	2.76 (2.25–3.38)	69.20%, <0.001	0.511
**Non-traditional risk factors**					
Education (< high school or ≥ high school)	18	452,300	2.01 (1.33–3.02)	99.20%, 0.001	0.342
Marital status (being unmarried or married)	4	48,193	1.60 (0.87–2.97)	96.10%, 0.134	0.875
Hyperuricemia (yes or no)	14	250,620	2.74 (1.40–5.36)	99.60%, 0.003	0.552
Anemia (yes or no)	3	43,714	2.80 (2.55–3.08)	0%, <0.001	0.486
Smoking status (current smoker or non-smoker)	32	583,337	0.83 (0.54–1.28)	99.40%, 0.398	0.960
Alcohol consumption (current drinker or non-drinker)	19	498,173	0.83 (0.56–1.24)	98.60%, 0.365	0.207
Family history of HT (yes or no)	3	6,842	2.23 (0.56–8.81)	94.90%, 0.254	NR
Family history of CKD (yes or no)	2	6,128	2.82 (1.73–4.59)	0%, <0.001	NR
Physical activity (inactive or active)	8	160,248	1.57 (0.97–2.53)	99.10%, 0.064	NR
NSAIDs use (yes or no)	4	70,661	1.97 (1.48–2.61)	56.60%, <0.001	NR
CD4 cell count^c^ (≥200 or <200 cells/ml)	2	6,090	1.08 (0.74–1.58)	41.10%, 0.679	NR

^a^ OR calculated using random-effect model;

^b^ 95% confidence interval,

^c^ CD4 cell count only in HIV patients;

^d^ the recalculated OR using trim-and-fill analysis with twelve adjusted studies;

CKD, chronic kidney disease; BMI, body mass index; CHD, coronary heart disease; CVD, cardiovascular disease; LDLc, low-density lipoprotein cholesterol; HDLc, high-density lipoprotein cholesterol; HT, hypertension; NSAIDs, non-steroidal anti-inflammatory drugs; NR, not reported;

### Sensitivity analyses

Because the included studies were found to be the high or moderate quality, sensitivity analysis was not performed as planned. The mean prevalence of CKD stages 3–5 was 11.0% in high-quality studies and 11.4% in moderate-quality studies. The estimated prevalence of CKD stages 3–5 by study designs was 8.2% for a cohort study and 11.4% for a cross-sectional study. The mean prevalence of CKD stages 3–5 by methods for measurement of eGFR was 19.7% for CG, 10.3% for eMDRD_175_, 10.9% for CKD-EPI and 12.3% for MDRD_186_. CKD stage 3–5 prevalence was higher in the studies with high proportions of hypertensive patients in the study populations (14.1%) than those with low proportions (9.4%). Similarly, the prevalence of CKD stage 3–5 in the studies with high proportions of diabetic patients in study populations (14.1%) was higher than those with low proportions (6.4%) (P > 0.05; Table 4).

**Table 4:** Subgroup analysis of the pooled prevalence of CKD stage 3–5 in low- and middle-income countries in Asia.

Subgroup	Pooled prevalence (95% CI)	I^2^, p-value	p-value for difference
**Geographical subregion in Asia**			
East Asia	8.64 (7.17–10.24)	99.94%, <0.001	0.039
Southeast Asia	11.95 (7.71–16.95)	99.80%, <0.001	
Western Asia	13.09 (8.68–18.25)	99.71%, <0.001	
South Asia	13.51 (9.54–18.05)	99.84%, <0.001	
**Economic group (classified by the World Bank)**			
Upper-middle income countries	9.82 (8.30–11.46)	99.94%, <0.001	<0.001
Lower-middle income countries	13.83 9.93–18.26)	99.83%, <0.001	
Low income countries	6.44 (5.67–7.25)	N/A, <0.001	
**Study design**			
Cross-sectional study	11.42 (9.45–13.55)	99.97%, <0.001	0.378
Cohort study	8.24 (3.06–15.61)	99.82%, <0.001	
**Methods for measurement of eGFR**			
CKD-EPI	10.87 (7.76–14.43)	99.98%, <0.001	<0.001
MDRD _186_	12.27 (9.33–15.55)	99.66%, <0.001	
eMDRD _175_	10.33 (7.70–13.30)	99.88%, <0.001	
Cockcroft-Gault	19.69 (16.14–23.49)	N/A, <0.001	
Not reported	10.20 (6.63–14.43)	99.31%, <0.001	
**Quality of study**			
High-quality study	10.98 (8.24–14.07)	99.96%, <0.001	0.836
Moderate-quality study	11.37 (9.24–13.70)	99.92%, <0.001	
**Gender of study populations**			
Male	9.09 (7.08–11.31)	99.87%, <0.001	0.563
Female	9.98 (7.94–12.23)	99.84%, <0.001	
**Proportions of hypertension patients in study populations**			
≤ 36.9%	9.37 (7.63–11.27)	99.92%, <0.001	0.055
> 36.9%	14.07 (10.72–17.79)	99.88%, <0.001	
Not reported	10.48 (3.95–19.64)	99.98%, <0.001	
**Proportions of diabetes patients in study populations**			
≤ 8.1%	6.41 (4.82–8.21)	99.91%, <0.001	<0.001
> 8.1%	14.11 (11.65–16.77)	99.86%, <0.001	
Not reported	10.95 (5.11–18.64)	99.98%, <0.001	

* estimated prevalence calculated using random-effect models; eGFR, estimated glomerular filtration rate; CKD, chronic kidney disease; EPI, epidemiology collaboration equation; MDRD_186_, Modification of Diet in Renal Disease Study with constant factor of 186; eMDRD_175_, estimated Modification of Diet in Renal Disease Study with constant factor of 175

## Discussion

CKD was rated as the 16^th^ highest cause for loss of life in the year 2017 [28], leading to poor health quality and very high costs of medical care [29]. Most people with CKD in stages 3–5 were undiagnosed and presented to seek care in the last stage [30]. This is the first systematic review and meta-analysis to assess the prevalence of CKD stages 3–5 and its associated risk factors in LMICs in Asia. The present study found that the current prevalence of CKD stages 3–5 in LMICs in Asia is 11.2% (95% CI; 9.3–13.2%), comprising 117 populations in LMICs across Asia. High CKD prevalence could lead to high treatment costs and a great macroeconomic burden [31]. Although, CKD commonly affects patients and families with insufficient resources [32], there are limited studies on the burden of CKD, especially in LMICs in Asia.

The estimated prevalence of CKD stages 3–5 in LMICs in Asia was found in this study resembles the global CKD stages 3–5 prevalence of 10.6% [1], and approximately 1 in every 10 people was affected by CKD (range from 0.7% to 61.4%). The prevalence of CKD stages 3–5 reported in this study was higher in south Asia (13.5%) and western Asia (13.1%) than those in south-east Asia (12.0%) and east Asia (8.6%). There was no relevant study from central Asia included in this meta-analysis. According to this study’s findings, the global prevalence of CKD stages 3–5 [1] is comparable to that reported in Thailand, India and Malaysia. The prevalence of CKD stages 3–5 in six countries (Philippines, Sri Lanka, Pakistan, Iran, Bangladesh and Mongolia) is higher than the global prevalence [1]. Conversely, lower levels of prevalence are observed in China, Indonesia, Vietnam, Nepal and Turkey, when compared with the global prevalence of CKD stages 3–5 [1]. Furthermore, this study found a wide range of estimated prevalence of CKD stages 3–5, which might be due to the different number of participants and high heterogeneity of their characteristics, in which Tau^2^ 0.12 and I^2^ 99.96% indicate high heterogeneity. Variations in CKD prevalence estimates appear to depend on many factors, such as target population, sample size and participation rate [3, 4, 33, 34]. The rising trend of global burden of CKD was reported from 1990 to 2019 due to hypertension [35]. This study found that the prevalence of CKD stages 3–5 in year 2011–2021 (12.4%) was higher than the years before 2011 (9.5%) in LMICs in Asia. Although CKD stage 3–5 prevalence in the year 2011–2021 was determined from larger populations (4.2 million in 68 reports) than the year before 2011 (0.5 million in 49 reports). The CKD stages 3–5 prevalence seemed to be higher using CG than MDRD_186_, CKD-EPI and eMDRD_175_. The differences in CKD stages 3–5 prevalence across studies in LMIC groups might be influenced by inadequately characterised data for CKD stage 3–5, disorganized data collection methods, inconsistent methods to identify kidney dysfunction, and used of different creatinine-based eGFR equations [4, 36, 37]. When countries with different economic classifications were compared, the prevalence of CKD stages 3–5 in lower-middle income countries was higher than upper-middle income countries. However, the comparison of the CKD stages 3–5 prevalence in low-income country and other groups must be interpreted carefully because CKD prevalence was available from only three studies. In LMICs, the country’s economy might affect the CKD stages 3–5 prevalence estimation due to differences in accessibility and quality of the CKD screening program from different health care systems when compared with high-income countries [3, 4, 33]. Previous studies have shown that individuals with a lower income were more likely to present CKD progression, which may be due to an inadequate diet, unhealthy lifestyle and poor access to health information and quality healthcare services [5, 38–40].

In addition, this study found 15 risk factors (10 traditional and five non-traditional risk factors) that are significantly associated with CKD stages 3–5 in LMICs in Asia. People had an increased risk of CKD stages 3–5 if they were elderly, had less than high school education, suffered from obesity, hypertension, diabetes, hypertriglyceridemia, hypercholesterolaemia, low levels of HDLc, hyperuricaemia, anemia, history of CHD, history of stroke, history of CVD, family history of CKD, and NSAIDs medication use. Previous studies that identified major risk factors for CKD included only the factors of age, education, obesity, diabetes, hypertension, stroke, hyperuricaemia and a family history of CKD [3, 5, 41–47]. Individuals with obesity and unhealthy metabolic syndrome had a high risk of CKD [46, 48]. In particular, this study found an association between non-traditional risk factors unrelated to diabetes, hypertension or the other traditional causes of CKD and uncertain etiology (CKDu). In this meta-analysis study, five risk factors were found to be significantly associated with CKDu, including education lower than high school, hyperuricemia, anemia, history of CKD among family members and NSAIDs medication use. In addition, there were two individual studies reported other CKDu risk factors, such as environmental factors (home location in the Indonesia study) [49] and occupational factors (agriculture in the Sri Lanka study) [50]. Thus, CKD is associated with a plurality of risk factors in traditional risk factors and non-traditional risk factors, in which a few studies were conducted. Similarly, previous systematic reviews reported that CKD non-traditional etiology (CKDnT) or CKD of uncertain (CKDu) was associated with the parent history of CKD and farmers exposed to agrochemicals, heavy metals, hard water, dehydration and heat-stress [51, 52]. Besides, there was significant association between NSAIDs medication use and CKD pooled from four cross-sectional surveys (three studies reported a significant association and one study reported an insignificant association), similarly to the previous meta-analysis reported that high-dose NSAID medication exposure would increase the risk of CKD [10]. Also, further research is required to explain the association between NSAIDs exposure and CKD stage 3–5. On the other hand, this meta-analysis reported no significant statistical association between CKD stages 3–5 and some risk factors, including gender, dyslipidemia, high LDLc level, smoking status, alcohol consumption and physical activity. Conversely, most studies found gender and smoking to be independent risk factors for CKD stages 3–5 [3, 41, 46, 53]. Two previous meta-analyses reported that gender and alcohol consumption were not associated with CKD [54, 55]. Besides, the insignificant association between CKD and dyslipidemia, low LDLc level and physical activity were pooled amongst the diversely individual study results. The meta-regression in this study showed no statistical effect of different study designs on associated risk factors and CKD. Some risk factors were analyzed using a small number of studies (<10 studies), including marital status, hypertriglyceridemia, hypercholesterolaemia, high LDLc, low HDLc, anemia, history of stroke, history of CVD, family history of CKD, family history of HT, physical activity, NSAIDs use, and CD4 cell count in HIV patients. Most studies were cross-sectional prevalence surveys in various targeted-populations and did not aim to determine the disease-related risk factors.

This meta-analysis consisted of individual studies with residual confounding and undetermined sources of bias. Unfortunately, these publication biases were difficult to quantify [3]. However, this study attempted to minimise the risk of bias with various methods [56]. This systematic review considered studies with a broad publication date and included articles that were published in more than one language (English and Thai) [57]. We also included unpublished literature identified from manual searches and conferences. All study selection processes were independently investigated by two reviewers. Observational studies were included through a homogeneous protocol regarding the identification of CKD stage 3–5 using Scr or eGFR for kidney function estimations [58]. Duplicate publications were excluded by reaching a consensus between two reviewers according to the criteria for comparing reports. The pooled prevalence identified in this study was estimated from studies with moderate to high quality. No discovered publication bias distorted the result of the estimated prevalence of CKD by the funnel plot and Egger’s test [59].

This study dealt with several limitations. First, the language of publications was restricted to only English and Thai, although grey literature and studies from relevant conferences were included. Second, there was high heterogeneity in the estimated prevalence across populations of different sizes due to differences in individual study criteria, including variations in the methods used to measure eGFR and the disparities of targeted-population, as well as the difference of national policies of each country, political commitment that drives Universal Health Coverage where access to health care is high, healthcare and information systems with limited resources to address the findings of chronic kidney disease prevalence. However, the author did make an effort to robust the included study quality by using NOS quality assessments and they were found to be moderate or high quality. In conclusion, this meta-analysis presented the current epidemiology of CKD stages 3–5 in LMICs in Asia. The findings of this meta-analysis might be of use in CKD diagnostics and aetiology research. In addition, the results might be used in future strategy planning to improve early detection, slow the progression and prevent CKD stages 3–5 in LMICs in Asia.

## Conclusion

This meta-analysis reported a prevalence of CKD stages 3–5 of 11.2% in LMICs in Asia, with the highest prevalence of 13.5% observed in countries in south Asia and the lowest prevalence of 8.6% among countries in east Asia. The estimated prevalence of CKD stages 3–5 in LMICs in Asia varied among individual countries and subregions.

## Supporting information

S1 Appendix Preferred reporting items for systematic reviews and meta-analyses (PRISMA) checklist. (DOCX)

S2 Appendix PRISMA 2020 for abstracts checklist. (DOCX)

S3 Appendix List of low- and middle-income counties in Asia. (PDF)

S4 Appendix Keyword search and search strategy. (PDF)

S5 Appendix Characteristics of included studies and Newcastle-Ottawa quality assessment scale (NOS). (PDF)

S6 Appendix Other forest plots in chronic kidney disease (CKD) prevalence and associated risk factors. (PDF)

S7 Appendix Funnel plots, Egger’ s test and trim-and-fill method in the pooled prevalence and associated risk factors of chronic kidney disease (CKD) in LMICs in Asia. (PDF)

S8 Appendix Prevalence of chronic kidney disease stages 3–5 in low- and middle-income countries in Asia: Protocol for a systematic review and meta-analysis. (PDF)
