# Supplementary material for: Prevalence of chronic kidney disease stages 3–5 in low- and middle-income countries in Asia: A systematic review and meta-analysis
Source: PLoS One. 2022 Feb 25;17(2):e0264393. doi: 10.1371/journal.pone.0264393 (PMC8880400; doi:10.1371/journal.pone.0264393)
Supplement: S3 Appendix — (PDF) [file pone.0264393.s003.pdf]

S3 Appendix. List of low- and middle- income counties in Asia [1, 2].

**A**	• Afghanistan<br>• Armenia<br>• Azerbaijan	**N**	• Nepal<br>• North Korea
**B**	• Bahrain<br>• Bangladesh<br>• Bhutan	**P**	• Pakistan<br>• Palestine<br>• Philippines
**C**	• Cambodia<br>• China<br>• Cyprus	**R**	• Russia
**G**	• Georgia	**S**	• Sri Lanka<br>• Syria
**I**	• India<br>• Indonesia<br>• Iran<br>• Iraq<br>• Israel	**T**	• Tajikistan<br>• Thailand<br>• Timor-Leste<br>• Turkey<br>• Turkmenistan
**J**	• Jordan	**U**	• Uzbekistan
**K**	• Kazakhstan<br>• Kuwait<br>• Kyrgyzstan	**V**	• Vietnam
**L**	• Laos<br>• Lebanon	**Y**	• Yemen
**M**	• Malaysia<br>• Maldives<br>• Mongolia<br>• Myanmar (formerly Burma)		

Reference to LMIC in Asia

1. COUNTRIES-ofthe-WORLD.COM [Internet]. List of countries in Asia 2020 [cited 2020 Nov 6]. Available from: https://www.countries-ofthe-world.com/.
2. Current classification by income [Internet]. 2029 [cited 15 Jan 2021]. Available from: https://datahelpdesk.worldbank.org/knowledgebase/articles/906519.
