# Supplementary material for: Prevalence of chronic kidney disease stages 3–5 in low- and middle-income countries in Asia: A systematic review and meta-analysis
Source: PLoS One. 2022 Feb 25;17(2):e0264393. doi: 10.1371/journal.pone.0264393 (PMC8880400; doi:10.1371/journal.pone.0264393)
Supplement: S4 Appendix — (PDF) [file pone.0264393.s004.pdf]

S4 Appendix. Keyword search and search strategy.

1. Keyword search

We would search the databases using the following free text and exploded MeSH terms:

1. Prevalence: prevalence, epidemiology, and burden

2. Chronic kidney disease: CKD, chronic kidney disease, chronic renal failure, chronic kidney failure, chronic renal insufficiency, chronic kidney insufficiency, ESRD, and end stage renal disease.

3. Region: Asia

2. Search strategy

Period of searching:

We searched the literature that published from inception until 30th Nov, 2021

Table S1. Systematic Review Search Strategy: between the group of prevalence key search, chronic kidney disease key search, and Asia

**Database**	**Search term**	**No. of Article**
PubMed/Medline	**#1** "renal insufficiency, chronic" [MeSH]	124,666
	**#2** "kidney failure, chronic" [MeSH]	97,581
	**#3** "CKD"	36,347
	**#4** "chronic kidney disease"	60,855
	**#5** "chronic renal failure"	24,343
	**#6** "chronic kidney insufficiency"	234
	**#7** "ESRD"	18,113
	**#8** "end stage renal disease"	36,079
	**#9** #1 OR #2 OR #3 OR #4 OR #5 OR #6 OR #7 OR #8	179,175
	**#10** "prevalence"[Mesh]	322,575
	**#11** "epidemiology"[Mesh]	27,861
	**#12** "burden"	271,927
	**#13** #10 OR #11 OR #12	603,328
	**#14** "asia" [Mesh]	948,389
	**#15** #9 AND #13	11,063
	**#16** #15 AND #14	1,774

Database	Search term	No. of Article
ScienceDirect	**#1** "chronic renal insufficiency"	1,154
	**#2** "chronic kidney failure"	363
	**#3** "CKD"	10,135
	**#4** "chronic kidney disease"	18,271
	**#5** "chronic renal failure"	5,573
	**#6** "chronic kidney insufficiency"	24
	**#7** "ESRD"	4,947
	**#8** "end stage renal disease"	10,476
	**#9** #1 OR #2 OR #3 OR #4 OR #5 OR #6 OR #7 OR #8	35,843
	**#10** "prevalence"	193,000
	**#11** "epidemiology"	67,363
	**#12** "burden"	87,989
	**#13** #10 OR #11 OR #12	324,979
	**#14** "asia*"	73,384
	**#15** #9 AND #13	5,857
	**#16** #15 AND #14	208
Scopus	**#1** "chronic renal insufficiency"	6,280
	**#2** "chronic kidney failure"	108,253

Database	Search term	No. of Article
	**#3** "CKD"	39,955
	**#4** "chronic kidney disease"	81,552
	**#5** "chronic renal failure"	31,472
	**#6** "chronic kidney insufficiency"	205
	**#7** "ESRD"	20,088
	**#8** "end stage renal disease"	54,312
	**#9** #1 OR #2 OR #3 OR #4 OR #5 OR #6 OR #7 OR #8	210,898
	**#10** "prevalence"	1,108,724
	**#11** "epidemiology"	639,769
	**#12** "burden"	408,616
	**#13** #10 OR #11 OR #12	1,973,200
	**#14** "asia*"	656,027
	**#15** #9 AND #13	32,712
	**#16** #15 AND #14	1,201
Cochrane Library	**#1** "renal insufficiency, chronic" [MeSH]	7,192
	**#2** "kidney failure,chronic" [MeSH]	4,867
	**#3** "CKD"	6,322
	**#4** "chronic kidney disease"	8,734

Database	Search term	No. of Article
	**#5** "chronic renal failure"	2,158
	**#6** "chronic kidney insufficiency"	15
	**#7** "ESRD"	2,225
	**#8** "end stage renal disease"	4,408
	**#9** #1 OR #2 OR #3 OR #4 OR #5 OR #6 OR #7 OR #8	17,080
	**#10** "prevalence"[Mesh]	4,881
	**#11** "epidemiology"[Mesh]	42
	**#12** "burden"	27,549
	**#13** #10 OR #11 OR #12	31,264
	**#14** "asia" [Mesh]	22,353
	**#15** #9 AND #13	609
	**#16** #15 AND #14	20
EMBASE	**#1** "chronic renal insufficiency"	33,167
	**#2** "chronic kidney failure"	47,549
	**#3** "CKD"	30,071
	**#4** "chronic kidney disease"	51,608
	**#5** "chronic renal failure"	34,184
	**#6** "chronic kidney insufficiency"	29,764

Database	Search term	No. of Article
	**#7** "ESRD"	18,135
	**#8** "end stage renal disease"	30,557
	**#9** #1 OR #2 OR #3 OR #4 OR #5 OR #6 OR #7 OR #8	77,935
	**#10** "prevalence"	339,289
	**#11** "epidemiology"	1,044,294
	**#12** "burden"	158,573
	**#13** #10 OR #11 OR #12	1,264,167
	**#14** "asia*"	143,365
	**#15** #9 AND #13	31,886
	**#16** #15 AND #14	1,306
Thai Library Integrated System (ThaiLIS)	**#1** "chronic renal insufficiency"	0
	**#2** "chronic kidney failure"	0
	**#3** "CKD"	165
	**#4** "chronic kidney disease"	115
	**#5** "chronic renal failure"	63
	**#6** "chronic kidney insufficiency"	0
	**#7** "ESRD"	29

Database	Search term	No. of Article
	**#8** "end stage renal disease"	47
	**#9** #1 OR #2 OR #3 OR #4 OR #5 OR #6 OR #7 OR #8	390
	**#10** "prevalence"	1,562
	**#11** "epidemiology"	557
	**#12** "burden"	768
	**#13** #10 OR #11 OR #12	2,755
	**#14** "asia"	3,025
	**#15** #9 AND #13	2
	**#16** #15 AND #14	2
Thai Thesis Database	**#1** "chronic renal insufficiency"	0
	**#2** "chronic kidney failure"	2
	**#3** "CKD"	149
	**#4** "chronic kidney disease"	102
	**#5** "chronic renal failure"	62
	**#6** "chronic kidney insufficiency"	0
	**#7** "ESRD"	26
	**#8** "end stage renal disease"	43

Database	Search term	No. of Article
	**#9** #1 OR #2 OR #3 OR #4 OR #5 OR #6 OR #7 OR #8	336
	**#10** "prevalence"	1,428
	**#11** "epidemiology"	539
	**#12** "burden"	705
	**#13** #10 OR #11 OR #12	2,549
	**#14** "asia"	2,692
	**#15** #9 AND #13	0
	**#16** #15 AND #14	0
Grey Literature (OpenSIGLE)	**#1** "chronic renal insufficiency"	16
	**#2** "chronic kidney failure"	46
	**#3** "CKD"	58
	**#4** "chronic kidney disease"	113
	**#5** "chronic renal failure"	115
	**#6** "chronic kidney insufficiency"	3
	**#7** "ESRD"	20
	**#8** "end stage renal disease"	61
	**#9** #1 OR #2 OR #3 OR #4 OR #5 OR #6 OR #7 OR #8	287

Database	Search term	No. of Article
	**#10** "prevalence"	3,612
	**#11** "epidemiology"	2,221
	**#12** "burden"	987
	**#13** #10 OR #11 OR #12	6,524
	**#14** "asia*"	3,523
	**#15** #9 AND #13	28
	**#16** #15 AND #14	0
