# Supplementary material for: Prevalence of chronic kidney disease stages 3–5 in low- and middle-income countries in Asia: A systematic review and meta-analysis
Source: PLoS One. 2022 Feb 25;17(2):e0264393. doi: 10.1371/journal.pone.0264393 (PMC8880400; doi:10.1371/journal.pone.0264393)
Supplement: S5 Appendix — (PDF) [file pone.0264393.s005.pdf]

S5 Appendix. Characteristic of included studies and Newcastle-Ottawa quality assessment scale (NOS).

Abbreviations:

eGFR, estimated glomerular filtration rate; CKD, chronic kidney disease; EPI, epidemiology collaboration equation; $MDRD_{186}$, Modification of Diet in Renal Disease Study with constant factor of 186; $MDRD_{175}$, Modification of Diet in Renal Disease Study with constant factor of 175; N/A, not applicable from studies.

Table S3. Characteristic of studies included in the meta-analysis.

Author	Year	Country	Targeted-population	Number of participants	Male (%)	HT (%)	DM (%)	Medication use (%)	Study design	eGFR equation	Newcastle-Ottawa Scale
Aekplakorn et al. *, † [1]	2021	Thailand	Age ≥20 years	17,329	48	28.5	9.8	-	Cross-sectional study	CKD-EPI	High
Alam et al.*, † [2]	2014	Pakistan	Age ≥15 years	350	36	20.9	8.6	-	Cross-sectional study	MDRD 186	High
Anand et al.*, † [3]	2014	Bangladesh	Age >30 years	357	51	37.2	-	-	Cross-sectional study	CKD-EPI	High
Anand et al.*, † [4]	2017	India	Age >40 years without self-reported heart disease or stroke	5,294	48.4	37.0	23.7	-	Cross-sectional study	CKD-EPI	High
Anupama et al. *, † [5]	2014	India	Age ≥18 years	2,091	45.6	33.6	3.8	-	Cross-sectional study	MDRD 186	High
Anupama et al.*, † [6]	2017	India	Age ≥18 years	1,331	44	32.6	6.6	-	Cross-sectional study	MDRD 186	High
Bahadoran et al.* [7]	2016	Iran	Age ≥20 years	3,462	13.4	17.9	11.5	-	Cross-sectional study	CKD-EPI	High
Bragg-Gresham et al. * [8]	2020	India	Age range of 18 - 69 years	2,002	58.2	48.2	4.0		Cross-sectional study	CKD-EPI	High
Cao et al.*, † [9]	2013	China	Age >18 years, all HIV-infected and ART-naive patients	538	74.2	2.8	-	-	Cross-sectional study	MDRD 186	High
Chen et al.* [10]	2008	China	Age ≥20 years	6,101	-	19.2	5.5	-	Cross-sectional study	eMDRD 175	High
Chen et al.*, † [11]	2011	China	Age ≥18 years	1,289	-	38.8	2.9	-	Cross-sectional study	eMDRD 175	High
Das et al. * [12]	2019	Bangladesh	Age ≥19 years	218,888	56.6	-	-	-	Cross-sectional study	CKD-EPI	High
Dhimal et al.* [13]	2019	Napal	Age ≥20 years	13,200	42.2	-	8.5	-	Cross-sectional study	N/A	Moderate
Ding et al. * [14]	2012	China	Men ≥55 years and women ≥65 years with stable premature coronary artery disease (CAD)	512	41	68	18.8	-	Cohort study	MDRD 186	Moderate
Domrongkitchaiporn et al.*, † [15]	2004	Thailand	Age range of 35 - 54 years	3,499	77.2	-	6.8	-	Cohort study	MDRD 186	Moderate
Du et al.*, † [16]	2017	China	Age range of 20 - 49 years, married group	3,091,379	48.3	4.1	0.7	-	Cross-sectional study	CKD-EPI	Moderate
Duan et al.*, † [17]	2019	China	Age ≥18 years	23,869	40.2	28.9	11.4	-	Cross-sectional study	CKD-EPI	High
Fan et al.* [18]	2013	China	Age range of 18 - 74 years	4,473	46.7	-	-	-	Cross-sectional study	CKD-EPI	High
Farag et al. *, † [19]	2020	India	Age ≥18 years	1,184	44.0	45.5	22.1		Cross-sectional study	CKD-EPI	Moderate
Farhadnejad et al.* [20]	2019	Tehran, Iran	Age ≥20 years	1,797	48	17.7	11.7	-	Cross-sectional study	MDRD 186	High
Fatema et al.* [21]	2013	Bangladesh	Age ≥18 years	634	88.3	25.2	8.5	-	Cross-sectional study	MDRD 186	Moderate
Feng et al.*, † [22]	2019	Bangladesh, Pakistan and Sri Lanka	Age ≥40 years, hypertensive adults	Bangladesh:868, Pakistan: 685, Sri Lanka:796	Bangladesh: 36.2, Pakistan: 39.3, Sri Lanka: 31.7	100.0	26.7	-	Cross-sectional study	CKD-EPI	High

Author	Year	Country	Targeted-population	Number of participants	Male (%)	HT (%)	DM (%)	Medication use (%)	Study design	eGFR equation	Newcastle-Ottawa Scale
Gallieni et al.* [23]	2013	India	Age ≥30 years	1,526	42.7	39.4	-	-	Cross-sectional study	MDRD 186	High
Guo et al.* [24]	2017	China	Elderly patients with type 2 Diabetes mellitus	21,723	48.7	62.4	100.0	-	Cross-sectional study	CKD-EPI	Moderate
Han et al.* [25]	2019	China	Age ≥60 years	966	18.1	23.5	5.0	-	Cross-sectional study	CKD-EPI	Moderate
Hasan et al. *, † [26]	2012	Bangladesh	Age range of 18-65 years	1,240	52.4	23.2	10.4		Cross-sectional study	MDRD 186	Moderate
He et al.*, † [27]	2016	China	Age ≥20 years, Chinese urban adults	123,629	63.2	23.1	6.6	-	Cross-sectional study	eMDRD 175	High
Herath et al.*, † [28]	2019	Sri Lanka	Age range of 18-93 years, healthy people	7,768	28.9	-	-	-	Cross-sectional study	CKD-EPI	Moderate
Hong et al.*, † [29]	2015	China	Age ≥18 years	41,131	41.9	33.5	7.5	-	Cross-sectional study	eMDRD 175	High
Hooi et al. *, † [30]	2013	Malaysia	Age >18 years	876	47.9	38.4	19.6	NSAIDs 18.8	Cross-sectional study	CKD-EPI	Moderate
Hosseinpanah et al.*, † [31]	2009	Iran	Age >20 years	10,063	42	25.8	13.7	-	Cross-sectional study	MDRD 186	High
Huda et al.* [32]	2012	Bangladesh	Age range of 15-65 years	1,000	33.4	11.6	4.1	-	Cross-sectional study	MDRD 186	Moderate
Hussain et al.*, † [33]	2019	India	Age ≥18 years	323	48.3	68.4	100.0	-	Cross-sectional study	CKD-EPI	High
Imran et al.* [34]	2015	Pakistan	Age ≥30 years	293	65	29.2	24.0	-	Cross-sectional study	CKD-EPI	Moderate
Ingsathit et al. *, † [35]	2009	Thailand	Age ≥18 years	3,459	45.5	27.5	11.9	-	Cross-sectional study	eMDRD 175	High
Ito et al.*, † [36]	2008	Vietnam	Age >40 years	8,505	35.2	30.5	-	NSAIDs 0.1	Cross-sectional study	MDRD 186	High
Jafar et al.* [37]	2005	Pakistan	Age ≥40 years	262	54.2	48.9	10.7	-	Cross-sectional study	MDRD 186	High
Jessani et al.*, † [38]	2014	Pakistan	Age ≥40 years	2,873	47.8	44.9	21.4	-	Cross-sectional study	CKD-EPI	High
Ji et al.* [39]	2017	China	Age >65 years	1,586	44.6	52.6	19.6	-	Cross-sectional study	CKD-EPI	High
Ji et al.*, † [40]	2019	China	Age ≥60 years	34,588	43.3	70.6	24.8	-	Cross-sectional study	eMDRD 175	High
Jiang et al.*, † [41]	2011	China	Age ≥30 years	4,944	44.9	10.6	13.7	-	Cross-sectional study	eMDRD 175	High
Kaewput et al.*, † [42]	2019	Thailand	Age ≥20 years, hypertension patients	9,776	44.1	100.0	11.7	-	Cross-sectional study	CKD-EPI	High
Khajehdehi et al.*, † [43]	2014	Iran	Age ≥18 years	9,404	35.9	-	-	-	Cross-sectional study	MDRD 186	Moderate
Khanam et al. *, † [44]	2016	Bangladesh	Age range of 18–80 years, type 2 diabetic patients	1,317	54.7	16.7	100.0		Cross-sectional study	eMDRD 175	Moderate
Kitiyakara et al.* [45]	2012	Thailand	All age groups	5,526	75	36.3	11.6	-	Cross-sectional study	CKD-EPI	Moderate
Kitiyakara et al. *, † [46]	2020	Thailand	Age range of 35-54 years	3,334	76.6	19.3	6.6		Cross-sectional study	CKD-EPI	High
Le et al.*, † [47]	2020	Vietnam	Age <80 years, type 2 diabetes patients	467	37.3	85.9	100.0	-	Cohort study	CKD-EPI	Moderate
Li et al.*, † [48]	2014	China	, having metabolic syndrome	1,724	37.3	-	-	-	Cross-sectional study	eMDRD 175	High
Li et al. * [49]	2021	China	Age ≥18 years	47,204	42.7	35.2	7.4		Cross-sectional study	eMDRD 175	High

Author	Year	Country	Targeted-population	Number of participants	Male (%)	HT (%)	DM (%)	Medication use (%)	Study design	eGFR equation	Newcastle-Ottawa Scale
Liang et al. *, † [50]	2021	China	Age >18 years	47,086	42.6	34.1	23.3	Nephrotoxic medications, 3.2%	Cross-sectional study	N/A	Moderate
Lin et al.*, † [51]	2017	China	Age ≥ 40 years	250,752	34.6	45.7	21.2		Cohort study	CKD-EPI	High
Liu et al.*, † [52]	2013	China	Acute coronary syndrome, with undiagnosed diabetes or pre-diabetes	2,232	66.4	19.2	11.0	Nephrotoxic medications 2.8	Cross-sectional study	eMDRD 175	High
Liu et al.*, † [53]	2020	China	Age range of 37–84 years	1,434	40.7	-	-	-	Cohort study	CKD-EPI	Moderate
Liu et al.*, † [54]	2013	China	Age range of 20-75 years with periodontal disease	1,268	33.3	90.6	-	-	Cross-sectional study	eMDRD 175	High
Liu et al.*, † [55]	2016	China	Age range of 18 - 80 years	26,655	58.5	15.7	2.9	-	Cross-sectional study	eMDRD 175	Moderate
Lu et al.* [56]	2016	China	Age ≥40 years	239,832	N/A	-	-	-	Cross-sectional study	CKD-EPI	Moderate
Luk et al.* [57]	2015	China, India, Philippines, and Vietnam	Age ≥18 years with type 2 Diabetes mellitus	China:3,415, India: 3,714, Philippines: 3,364, Vietnam: 692	N/A	88.0	100.0	-	Cross-sectional study	MDRD 186	Moderate
Lv et al.*, † [58]	2020	China	Age >45 years	6,267	44.5	45.3	15.9	-	Cross-sectional study	CKD-EPI	High
Mahapatra et al.* [59]	2016	India	Age ≥18 years	1,104	61.4	23.6	11.2	-	Cross-sectional study	MDRD 186	Moderate
Mahdavi-Mazdeh et al.*, † [60]	2010	Iran	Age range of 18–86 years, taxi driver	31,999	98.4	-	-	-	Cross-sectional study	MDRD 186	Moderate
Mohanty et al.* [61]	2020	India	Age range of 20–60 years	2,978	37.3	11.7	5.7	-	Cross-sectional study	MDRD 186	High
Naghibi et al.*, † [62]	2015	Iran	Age range of 20–60 years	1,285	41	22.0	10.5	-	Cross-sectional study	MDRD 186	Moderate
Najafi et al.*, † [63]	2010	Iran	Age ≥18 years	3,591	39	90.3	49.7	-	Cross-sectional study	MDRD 186	Moderate
Nampoothiri et al.* [64]	2019	India	Hepatic steatosis patients	100	56	26.0	20.0	-	Cross-sectional study	CG	High
Nand et al.* [65]	2015	India	Age ≥20 years, having any metabolic syndrome	300	49.7	-	-	-	Cross-sectional study	MDRD 186	Moderate
Naseem et al.* [66]	2017	Pakistan	Age >18 years with essential hypertension	772	60	100.0	55.7	-	Cross-sectional study	N/A	Moderate
Ong-ajyooth et al.*, † [67]	2009	Thailand	Age ≥15 years	3,117	49.1	22.5	7.0	-	Cross-sectional study	MDRD 186	High
Pan et al.*, † [68]	2014	China	Age ≥18 years	47,204	42.7	14.7	3.4	-	Cross-sectional study	eMDRD 175	High
Pan et al.*, † [69]	2015	China	Age >18 years	7,588	47	35.4	7.4	NSAIDs 3.6	Cross-sectional study	CKD-EPI	High
Perkovic et al.* [70]	2007	Thailand	Age ≥35 years	5,146	48.7	21.2	9.7	-	Cross-sectional study	MDRD 186	Moderate
Pongpirul et al.*, † [71]	2017	Thailand	Age >18 years, HIV patients	5,552	N/A	16.6	4.0	-	Cohort study	CKD-EPI	Moderate
Prodjosudjadi et al.*, † [72]	2009	Indonesia	Age range of 18 - 70 years	9,412	35.9	15	3.5	-	Cross-sectional study	eMDRD 175	Moderate

Author	Year	Country	Targeted-population	Number of participants	Male (%)	HT (%)	DM (%)	Medication use (%)	Study design	eGFR equation	Newcastle-Ottawa Scale
Qiu et al.*, † [73]	2019	China	Age ≥18 years	33,300	40.8	31.8	7.7	-	Cross-sectional study	CKD-EPI	High
Rahim et al. *, † [74]	2017	Bangladesh	Type 2 Diabetes mellitus patients	400	42.2	57.2	100.0		Cross-sectional study	CKD-EPI	Moderate
Rai et al.* [75]	2019	India	Overweight and obese general population	103	68	32.4	21.4	-	Cross-sectional study	eMDRD 175	High
Rai et al.* [76]	2019	India	Age ≥45 years	198	62.6	22.2	13.6	-	Cross-sectional study	eMDRD 175	Moderate
Rajput et al. * [77]	2017	India	Age >18 years	3,000	54.0	53.8	100.0		Cross-sectional study	eMDRD 175	Moderate
Ruwanpathirana et al.*, † [78]	2019	Sri Lanka	Age ≥18 years	4,803	31.8	-	-	-	Cross-sectional study	CKD-EPI	High
Saber et al.*, † [79]	2017	Iran	Age range of 15-75 years	988	42	20.0	14.0	-	Cross-sectional study	MDRD 186	Moderate
Sahin et al.*, † [80]	2009	Turkey	Age ≥18 years	1,079	49.4	38.1	5.3	-	Cross-sectional study	eMDRD 175	Moderate
Satirapoj et al.*,† [81]	2013	Thailand	Age range of 15 - 60 years	15,357	80.4	6.8	2.6	-	Cross-sectional study	eMDRD 175	Moderate
Sepanlou et al.*, † [82]	2017	Iran	Age range of 40-75 years	11,409	47.4	43.7	15.6	-	Cohort study	MDRD 186	Moderate
Shan et al. *, † [83]	2010	China	Age >40 years	3,981	54.7	15.0	5.8	-	Cross-sectional study	MDRD 186	High
Sharma et al. * [84]	2010	China, Mongolia and Nepal	China; age range of 15 - 75 years, Mongolia; age >18 years and Nepal; age ≥20 years	China; 1,999, Mongolia; 997 and Nepal; 8,398	China; 27, Mongolia ; 26 and Nepal; 38	China; 4.6, Mongolia; 10.0 and Nepal; 3.3	China; 27.0, and Nepal; 21.0		Cross-sectional study	eMDRD 175	Moderate
Sharma et al.* [85]	2013	Nepal	Age ≥20 years	1,000	-	38.6	7.5	-	Cross-sectional study	MDRD 186	Moderate
Shen et al.*, † [86]	2019	China	Age ≥18 years	1,627	37	-	-	-	Cross-sectional study	eMDRD 175	High
Shi et al. * [87]	2019	China	Age >18 years	8,429	47.2	25.8	10.2	-	Cohort study	CKD-EPI	High
Shi et al. *, † [88]	2020	China	Age ≥35 years	11,694	46.3	50.1	10.4		Cross-sectional study	CKD-EPI	Moderate
Singh et al.*, † [89]	2009	India	Age ≥20 years	5,252	60	43.1	18.8	-	Cross-sectional study	MDRD 186	High
Singh et al.*, † [90]	2013	India	Age >18 years	5,588	55.1	37.3	10.4	NSAIDs 9.3	Cross-sectional study	eMDRD 175	Moderate
Song et al.* [91]	2019	China	Age ≥40 years	3,279	37.3	60.6	-	-	Cross-sectional study	CKD-EPI	High
Sun et al.*, † [92]	2017	China	Age range of 21–94 years with type 2 Diabetes mellitus	1,401	57.2	74.2	100.0	-	Cross-sectional study	CKD-EPI	High
Tatapudi et al. *, † [93]	2019	India	Age >18 years	2,210	44.3	16.7	7.2		Cross-sectional study	N/A	High
Trivedi et al.* [94]	2016	India	Age ≥18 years	2,350	38.8	26.8	9.8	-	Cross-sectional study	MDRD 186	Moderate
Varma et al.* [95]	2010	India	Age ≥18 years	2,595	66	13.2	1.5	-	Cross-sectional study	CKD-EPI	Moderate
Varma et al.* [96]	2010	India	Age >20 years, all healthy adults	1,920	N/A	8.5	1.1	-	Cross-sectional study	MDRD 186	Moderate
Wang et al. *, † [97]	2015	China	Age ≥45 years	8,659	47.6	63.8	26.4	-	Cross-sectional study	CKD-EPI	High

Author	Year	Country	Targeted-population	Number of participants	Male (%)	HT (%)	DM (%)	Medication use (%)	Study design	eGFR equation	Newcastle-Ottawa Scale
Wang et al. *, † [98]	2015	China	Age range of 40-60 years	2,379	51.9	22.1	2.8	-	Cohort study	CKD-EPI	High
Wang et al.*, † [99]	2018	China	Age >20 years	46,949	50	30.4	5.0	-	Cross-sectional study	CKD-EPI	High
Wen et al. *, † [100]	2017	China	Age >18 years	48,054	57.9	8.9	2.2	-	Cross-sectional study	CKD-EPI	Moderate
Xu et al. *, † [101]	2020	China	Age range of 45-59 years	2,004	50.0	14.3	10.6		Cross-sectional study	CKD-EPI	High
Yang et al.* [102]	2015	China	Age ≥ 40 years with obese	1,115	31.3	-	-	-	Cross-sectional study	eMDRD 175	High
Yang et al.*, † [103]	2019	China	Age range of 27-95 years	4,221	40.6	-	-	-	Cross-sectional study	CKD-EPI	High
Yang et al.*, † [104]	2019	China	Age range of 60-92 years	4,242	63.6	-	-	-	Cross-sectional study	CKD-EPI	High
Yang et al. *, † [105]	2021	China	Age range of 20-74 years	36,077	40.5	57.4	14.1		Cross-sectional study	eMDRD 175	High
Yuzbashian et al.* [106]	2016	Iran	Age >27 years	2,382	46	26.0	6.3	-	Cross-sectional study	MDRD 186	High
Zaman et al.*, † [107]	2018	Thailand	Age ≥18 years, type 2 diabetes patients	3,748	35.3	55.2	100.0	-	Cross-sectional study	eMDRD 175	High
Zhang et al.*, † [108]	2019	China	Age range of 18-79 years, type 2 diabetes patients	1,025	74	47.4	100.0	-	Cross-sectional study	MDRD 186	High
Zhang et al.*, † [109]	2020	China	Age >18 years	11,280	58.4	26.0	-	-	Cohort study	eMDRD 175	Moderate
Zhang et al.*, † [110]	2020	China	Age >18 years, diabetes patients	361	63.7	58.7	100.0		Cross-sectional study	CKD-EPI	High

* Studies included in pooled estimation of CKD prevalence in LMICs in Asia

† Studies included in pooled estimation of CKD associated risk factors in LMICs in Asia

Table S4. Newcastle-Ottawa quality assessment scale (NOS) of included studies in the Systematic Review: A cross-sectional studies.

**Author, Year**	**Selection**				**Comparability**	**Outcome**		**Total Score**	**Quality assessment level**
	1. Representativeness of the sample	2.Sample size	3.Non-respondents	4.Ascertainment of the exposure (risk factor)	1.Comparability of subjects in different outcome groups on the basis of design or analysis. Confounding factors controlled.	1.Assessment of outcome	2.Statistical test		
Aekplakorn et al., 2021 [1]	*		*	*	**	**	*	8	High
Alam et al., 2014 [2]	*		*	*	**	**	*	8	High
Anand et al., 2014 [3]	*		*	*	**	**		7	High
Anand et al., 2017 [4]	*		*	**	**	**	*	9	High
Anupama et al., 2014 [5]	*			*	**	**	*	7	High
Anupama et al., 2017 [6]		*		*	**	**	*	7	High
Bahadoran et al., 2016 [7]			*	*	**	**	*	7	High
Bragg-Gresham et al., 2020 [8]	*		*	**	**	**	*	9	High
Cao et al., 2013 [9]			*	**	**	**	*	8	High
Chen et al., 2008 [10]	*		*	*	**	**	*	8	High
Chen et al., 2011 [11]	*		*	*	**	**	*	8	High
Das et al., 2019 [12]				**	**	**	*	7	High
Dhimal et al., 2019 [13]	*	*	*	*		**		6	Moderate
Du et al., 2017 [16]			*	*		**	*	5	Moderate
Duan et al.,2019 [17]	*		*	*	**	**	*	8	High
Fan et al., 2013 [18]	*		*	**	**	**	*	9	High
Farag et al., 2020 [19]	*			**	**	**	*	8	High
Farhadnejad et al., 2019 [20]	*			*	**	**	*	7	High
Fatema et al., 2013 [21]				*	**	**	*	6	Moderate
Feng et al., 2019 [22]	*			*	**	**	*	7	High
Gallieni et al., 2013 [23]				**	**	**	*	7	High
Guo et al., 2017 [24]			*	**		**	*	6	Moderate
Han et al., 2019 [25]	*			*		**	*	5	Moderate
Hasan et al., 2012 [26]		*		*		**		4	Moderate
He et al., 2015 [27]			*	*	**	**	*	7	High
Herath et al., 2019 [28]				*	**	**		5	Moderate

**Author, Year**	**Selection**				**Comparability**	**Outcome**		**Total Score**	**Quality assessment level**
	1. Representativeness of the sample	2.Sample size	3.Non-respondents	4.Ascertainment of the exposure (risk factor)	1.Comparability of subjects in different outcome groups on the basis of design or analysis. Confounding factors controlled.	1.Assessment of outcome	2.Statistical test		
Hong et al., 2015 [29]	*		*	*	**	**	*	8	High
Hooi et al., 2013 [30]	*		*	*		**	*	6	Moderate
Hosseinpanah et al., 2009 [31]	*		*	**	**	**	*	9	High
Huda et al., 2012 [32]	*			*		**	*	5	Moderate
Hussain et al., 2019 [33]	*		*	*	**	**	*	8	High
Imran et al., 2015 [34]				*		**	*	4	Moderate
Ingsathit et al., 2009 [35]	*	*		*	**	**	*	8	High
Ito et al., 2008 [36]			*	**	**	**	*	8	High
Jafar et al., 2005 [37]	*		*	*	**	**	*	8	High
Jessani et al.,2014 [38]	*	*	*	**	**	**	*	10	High
Ji et al., 2017 [39]			*	*	**	**	*	7	High
Ji et al., 2019 [40]	*		*	*	**	**	*	8	High
Jiang et al., 2011 [41]	*		*	**	**	**	*	9	High
Kaewput et al., 2019 [42]	*			*	**	**	*	7	High
Khajehdehi et al., 2014 [43]	*	*		*		**	*	6	Moderate
Khanam et al., 2016 [44]				**		**	*	5	Moderate
Kitiyakara et al., 2012 [45]				*		**	*	4	Moderate
Kitiyakara et al., 2020 [46]			*	*	**	**	*	7	High
Li et al., 2014 [48]	*		*	*	**	**	*	8	High
Li et al., 2021 [49]	*		*	*	**	**	*	8	High
Liang et al., 2021 [50]	*				**		*	4	Moderate
Liu et al., 2013 [52]			*	*	**	**	*	7	High
Liu et al., 2013 [54]			*	**	**	**	*	8	High
Liu et al., 2016 [55]				*	**	**	*	6	Moderate
Lu et al., 2016 [56]			*	*	**	*	*	6	Moderate
Luk et al., 2015 [57]				*		**	*	4	Moderate
Lv et al., 2020 [58]	*			*	**	**	*	7	High
Mahapatra et al., 2016 [59]				*		**	*	4	Moderate

**Author, Year**	**Selection**				**Comparability**	**Outcome**		**Total Score**	**Quality assessment level**
	1. Representativeness of the sample	2.Sample size	3.Non-respondents	4.Ascertainment of the exposure (risk factor)	1.Comparability of subjects in different outcome groups on the basis of design or analysis. Confounding factors controlled.	1.Assessment of outcome	2.Statistical test		
Mahdavi-Mazdeh et al., 2010 [60]				*	**	**	*	6	Moderate
Mohanty et al., 2020 [61]	*	*		**		**	*	7	High
Naghibi et al., 2015 [62]	*			**		**		5	Moderate
Najafi et al., 2010 [63]				*	**	**	*	6	Moderate
Nampoothiri et al., 2019 [64]	*			*	**	**	*	7	High
Nand et al., 2015 [58]				**		**	*	5	Moderate
Naseem et al., 2017 [66]			*	*		*	*	4	Moderate
Ong-ajyooth et al., 2009 [67]	*		*	*	**	**	*	8	High
Pan et al., 2014 [68]	*			*	**	**	*	7	High
Pan et al., 2015 [69]	*		*	*	**	**	*	8	High
Perkovic et al., 2007 [70]	*		*	*		**	*	6	Moderate
Prodjosudjadi et al., 2009 [72]				*	**	**		5	Moderate
Qiu et al., 2019 [73]	*			*	**	**	*	7	High
Rahim et al., 2017 [74]				**		**		4	Moderate
Rai et al., 2019 [75]		*		**	**	**	*	8	High
Rai et al., 2019 [76]				*	**	**	*	6	Moderate
Rajput et al., 2017 [77]				**		**		4	Moderate
Ruwanpathirana et al., 2019 [78]	*		*	*	**	**	*	8	High
Saber et al., 2017 [79]	*	*		*		**		5	Moderate
Sahin et al., 2009 [80]	*	*		**		**		6	Moderate
Satirapoj et al., 2013 [81]				*	**	**	*	6	Moderate
Shan et al., 2009 [83]	*		*	*	**	**	*	8	High
Sharma et al., 2010 [84]				**		**		4	Moderate
Sharma et al., 2013 [85]			*	*		**	*	5	Moderate
Shen et al., 2019 [86]	*	*	*	*		**	*	7	High
Shi et al., 2020 [88]				*	**	**	*	6	Moderate
Singh et al., 2009 [89]	*		*	*	**	**	*	8	High
Singh et al., 2013 [90]				*		**	*	4	Moderate

Author, Year	Selection				Comparability	Outcome		Total Score	Quality assessment level
	1. Representativeness of the sample	2.Sample size	3.Non-respondents	4.Ascertainment of the exposure (risk factor)	1.Comparability of subjects in different outcome groups on the basis of design or analysis. Confounding factors controlled.	1.Assessment of outcome	2.Statistical test		
Song et al., 2019 [91]	*			*	**	**	*	7	High
Sun et al., 2017 [92]				**	**	**	*	7	High
Tatapudi et al., 2019 [93]	*	*	*	*	**	**	*	9	High
Trivedi et al., 2016 [94]				*		**	*	4	Moderate
Varma et al., 2010 [95]				*		**	*	4	Moderate
Varma et al., 2010 [96]				*		**	*	4	Moderate
Wang et al., 2015 [97]	*		*	**	**	**	*	9	High
Wang et al., 2018 [99]	*	*	*	*	**	**	*	9	High
Wen et al., 2017 [100]				*	**	**	*	6	Moderate
Xu et al., 2020 [101]	*		*	*	**	**	*	8	High
Yang et al., 2015 [102]	*			**	**	**	*	8	High
Yang et al., 2019 [103]	*			*	**	**	*	7	High
Yang et al., 2019 [104]	*			*	**	**	*	7	High
Yang et al., 2021 [105]	*			*	**	**	*	7	High
Yuzbashian et al., 2015 [106]	*			*	**	**	*	7	High
Zaman et al., 2018 [107]	*			**	**	**	*	8	High
Zhang et al., 2019 [108]	*			**	**	**	*	8	High
Zhang et al., 2020 [110]	*			**	**	**	*	8	High

Study quality Scoring: High = 7-10 points, Moderate= 4-6 points, and Poor = 0-3 points

Table S5. Newcastle-Ottawa quality assessment scale (NOS) of included studies in the Systematic Review: A cohort studies

**Author, Year**	**Selection**				**Comparability**	**Outcome**			**Total Score**	**Quality assessment level**
	1. Representative -ness of the exposed cohort	2. Selection of the non-exposed cohort	3. Ascertainment of exposure	4. Demonstration that outcome of interest was not present at start of study	1. Comparability of cohorts on the basis of the design or analysis	1. Assessment of outcome	2. Was follow-up long enough for outcomes to occur?	3.Adequacy of follow up of cohorts		
Ding et al., 2012 [14]			*	*		*	*	*	5	Moderate
Domrongkitchaiporn et al, 2004 [15]			*	*	*	*	*	*	6	Moderate
Le et al., 2020 [47]	*	*	*			*	*		5	Moderate
Lin et al., 2017 [51]	*	*	*	*	**	*	*	*	9	High
Liu et al., 2020 [53]		*	*	*	*		*	*	6	Moderate
Pongpirul et al., 2017 [71]			*	*	*	*	*		5	Moderate
Sepanlou et al., 2017 [82]	*	*			**	*	*		5	Moderate
Shi et al., 2019 [87]	*	*	*		**		*	*	7	High
Wang et al., 2015 [98]	*	*	*	*	*	*	*	*	8	High
Zhang et al., 2020 [109]			*	*	*	*		*	5	Moderate

Study quality Scoring: High = 7-9 points, Moderate= 4-6 points, and Poor = 0-3 points

References to included studies and Newcastle-Ottawa quality assessment scale (NOS).

1. Aekplakorn W, Chariyalertsak S, Kessomboon P, Assanangkornchai S, Taneepanichskul S, Neelapaichit N, et al. Women and other risk factors for chronic kidney disease of unknown etiology in Thailand: National Health Examination V Survey. Scientific reports. 2021;11(1). doi: 10.1038/s41598-021-00694-9.
2. Alam A, Amanullah F, Baig-Ansari N, Lotia-Farrukh I, Khan FS. Prevalence and risk factors of kidney disease in urban Karachi: Baseline findings from a community cohort study. BMC Res Notes. 2014;7(1). doi: 10.1186/1756-0500-7-179. PubMed Central PMCID: PMC24670059.
3. Anand S, Khanam MA, Saquib J, Saquib N, Ahmed T, Alam DS, et al. High prevalence of chronic kidney disease in a community survey of urban Bangladeshis: A cross-sectional study. Globalization Health. 2014;10(1). doi: 10.1186/1744-8603-10-9. PubMed Central PMCID: PMC24555767.
4. Anand S, Kondal D, Montez-Rath M, Zheng Y, Shivashankar R, Singh K, et al. Prevalence of chronic kidney disease and risk factors for its progression: A cross-sectional comparison of Indians living in Indian versus U.S. cities. PLoS ONE. 2017;12(3). doi: 10.1371/journal.pone.0173554. PubMed Central PMCID: PMC28296920.
5. Anupama Y, Uma G. Prevalence of chronic kidney disease among adults in a rural community in South India: Results from the kidney disease screening (KIDS) project. Indian journal of nephrology. 2014;24(4):214.
6. Anupama YJ, Hegde SN, Uma G, Patil M. Hypertension is an important risk determinant for chronic kidney disease: results from a cross-sectional, observational study from a rural population in South India. Journal of human hypertension. 2017;31(5):327-32. Epub 2016/11/25. doi: 10.1038/jhh.2016.81. PubMed PMID: 27882930.
7. Bahadoran Z, Mirmiran P, Tahmasebi Nejad Z, Ghasemi A, Azizi F. Serum nitric oxide is associated with the risk of chronic kidney disease in women: Tehran lipid and glucose study. Scandinavian journal of clinical and laboratory investigation. 2016;76(4):304-8. Epub 2016/03/10. doi: 10.3109/00365513.2016.1149880. PubMed PMID: 26956540.
8. Bragg-Gresham J, Thakur JS, Jeet G, Jain S, Pal A, Prasad R, et al. Population-based comparison of chronic kidney disease prevalence and risk factors among adults living in the Punjab, Northern India and the USA (2013-2015). BMJ open. 2020;10(12):e040444. Epub 2020/12/16. doi: 10.1136/bmjopen-2020-040444. PubMed PMID: 33318117; PubMed Central PMCID: PMCPMC7737106.
9. Cao Y, Gong M, Han Y, Xie J, Li X, Zhang L, et al. Prevalence and risk factors for chronic kidney disease among HIV-infected antiretroviral therapy-naïve patients in Mainland China: A multicenter cross-sectional study. Nephrology. 2013;18(4):307-12. doi: 10.1111/nep.12031. PubMed Central PMCID: PMC23311442.
10. Chen W, Chen W, Wang H, Dong X, Liu Q, Mao H, et al. Prevalence and risk factors associated with chronic kidney disease in an adult population from southern China. Nephrology Dialysis Transplantation. 2009;24(4):1205-12.
11. Chen W, Liu Q, Wang H, Chen W, Johnson RJ, Dong X, et al. Prevalence and risk factors of chronic kidney disease: a population study in the Tibetan population. Nephrology, dialysis, transplantation. 2011;26(5):1592-9. doi: 10.1093/ndt/gfq608. PubMed PMID: CN-00802521.

12. Das SK, Afsana SM, Elahi SB, Chisti MJ, Das J, Mamun AA, et al. Renal insufficiency among urban populations in Bangladesh: A decade of laboratory-based observations. 2019;14(4):e0214568.
13. Dhimal M, Karki KB, Sharma SK, Aryal KK, Shrestha N, Poudyal A, et al. Prevalence of Selected Chronic Non-Communicable Diseases in Nepal. J Nepal Health Res Counc. 2019;17(3):394-401. Epub 2019/11/19. doi: 10.33314/jnhrc.v17i3.2327. PubMed PMID: 31735938.
14. Ding Z, Wang X, Chen Z, Zhang X, Tang C, Feng Y, et al. Chronic kidney disease predicts poor prognosis in patients with stable premature coronary artery disease. Eur J Intern Med. 2012;23(8):716-9. doi: 10.1016/j.ejim.2012.07.003. PubMed Central PMCID: PMC22857882.
15. Domrongkitchaiporn S, Sritara P, Kitiyakara C, Stitchantrakul W, Krittaphol V, Lolekha P, et al. Risk factors for development of decreased kidney function in a Southeast Asian population: A 12-year cohort study. J Am Soc Nephrol. 2005;16(3):791-9. doi: 10.1681/ASN.2004030208. PubMed Central PMCID: PMC15677313.
16. Du Y, Zhang S, Hu M, Wang Q, Shen H, Zhang Y, et al. Prevalence of chronic kidney disease markers: Evidence from a three-million married population with fertility desire in rural China. Sci Rep. 2017;7(1). doi: 10.1038/s41598-017-02355-2. PubMed Central PMCID: PMC28578426.
17. Duan J, Wang C, Liu D, Qiao Y, Pan S, Jiang D, et al. Prevalence and risk factors of chronic kidney disease and diabetic kidney disease in Chinese rural residents: a cross-sectional survey. Sci Rep. 2019;9(1):10408. doi: 10.1038/s41598-019-46857-7.
18. Fan KL, Zhang HF, Zhu ZY, Yao WM, Shen J, Liang NX, et al. Association of CYBA rs7195830 polymorphism with estimated glomerular filtration rate in an adult Han sample from Jiangsu province, China. Chin Med J. 2013;126(17):3311-5. doi: 10.3760/cma.j.issn.0366-6999.20123332. PubMed Central PMCID: PMC24033955.
19. Farag YM, Karai Subramanian K, Singh VA, Tatapudi RR, Singh AKJRF. Occupational risk factors for chronic kidney disease in Andhra Pradesh:'Uddanam Nephropathy'. 2020;42(1):1032-41.
20. Farhadnejad H, Asghari G, Emamat H, Mirmiran P, Azizi F. Low-Carbohydrate High-Protein Diet is Associated With Increased Risk of Incident Chronic Kidney Diseases Among Tehranian Adults. J Ren Nutr. 2019;29(4):343-9. Epub 2018/12/24. doi: 10.1053/j.jrn.2018.10.007. PubMed PMID: 30579675.
21. Fatema K, Abedin Z, Mansur A, Rahman F, Khatun T, Sumi N, et al. Screening for chronic kidney diseases among an adult population. Saudi Journal of Kidney Diseases and Transplantation. 2013;24(3):534.
22. Feng L, De Silva HA, Jehan I, Naheed A, Kasturiratne A, Himani G, et al. Regional variation in chronic kidney disease and associated factors in hypertensive individuals in rural South Asia: Findings from control of blood pressure and risk attenuation - Bangladesh, Pakistan and Sri Lanka. Nephrology Dialysis Transplantation. 2019;34(10):1723-30. doi: 10.1093/ndt/gfy184.
23. Gallieni M, Ene-Iordache B, Aiello A, Tucci B, Sala V, Brahmochary Mandal SK, et al. Hypertension and kidney function in an adult population of West Bengal, India: Role of body weight, waist circumference, proteinuria and rural area living. Nephrology. 2013;18(12):798-807. doi: 10.1111/nep.12142. PubMed Central PMCID: PMC23889782.
24. Guo M, Niu JY, Ye XW, Han XJ, Zha Y, Hong Y, et al. Evaluation of various equations for estimating renal function in elderly Chinese patients with type 2 diabetes

mellitus. Clin Interventions Aging. 2017;12:1661-72. doi: 10.2147/CIA.S140289. PubMed Central PMCID: PMC29070944.
25. Han QX, Zhang D, Zhao YL, Liu L, Li J, Zhang F, et al. Analysis of chronic kidney disease staging with different estimated glomerular filtration rate equations in Chinese centenarians. Chin Med J. 2019;132(5):512-8. doi: 10.1097/CM9.0000000000000079.
26. Hasan MJ, Kashem MA, Rahman MH, Qudduhush R, Rahman M, Sharmeen A, et al. Prevalence of chronic kidney disease (CKD) and identification of associated risk factors among rural population by mass screening. 2012;1(1):20-6.
27. He Y, Li F, Wang F, Ma X, Zhao X, Zeng Q. The association of chronic kidney disease and waist circumference and waist-to-height ratio in Chinese urban adults. Medicine. 2016;95(25):e3769. Epub 2016/06/24. doi: 10.1097/md.0000000000003769. PubMed PMID: 27336864; PubMed Central PMCID: PMCPMC4998302 publication elsewhere; and that it has been submitted with the full knowledge and approval of the institution or organization given as the affiliation of the author(s). The authors have no conflicts of interest.
28. Herath N, Dassanayake R, Dissanayake M, Janitha C, Weerakoon K, Kumarasinghe C, et al. Normality data of eGFR and validity of commonly used screening tests for CKD in an area with endemic CKD of unknown etiology; need for age and sex based precise cutoff values. BMC Nephrol. 2019;20(1):298. Epub 2019/08/07. doi: 10.1186/s12882-019-1477-9. PubMed PMID: 31382902; PubMed Central PMCID: PMCPMC6683421.
29. Hong D, Zhang Y, Gao B, Wang J, Li G, Wang L, et al. Metabolic syndrome without diabetes or hypertension still necessitates early screening for chronic kidney disease: Information from a Chinese national cross-sectional study. PLoS ONE. 2015;10(7). doi: 10.1371/journal.pone.0132220. PubMed Central PMCID: PMC26161991.
30. Hooi LS, Ong LM, Ahmad G, Bavanandan S, Ahmad NA, Naidu BM, et al. A population-based study measuring the prevalence of chronic kidney disease among adults in West Malaysia. Kidney international. 2013;84(5):1034-40.
31. Hosseinpanah F, Kasraei F, Nassiri AA, Azizi F. High prevalence of chronic kidney disease in Iran: a large population-based study. BMC public Health. 2009;9(1):1-8.
32. Huda MN, Alam KS. Prevalence of chronic kidney disease and its association with risk factors in disadvantageous population. International journal of nephrology. 2012;2012.
33. Hussain S, Habib A, Najmi AK. Limited knowledge of chronic kidney disease among type 2 diabetes mellitus patients in india. International Journal of Environmental Research and Public Health. 2019;16(8). doi: 10.3390/ijerph16081443.
34. Imran S, Shiekh A, Saeed Z, Khan SA, Malik AO, Patel J. Burden of chronic kidney disease in an urban city of Pakistan, a cross-sectional study. J Pak Med Assoc. 2015;65(4):366-9.
35. Ingsathit A, Thakkinstian A, Chaiprasert A, Sangthawan P, Gojaseni P, Kiattisunthorn K, et al. Prevalence and risk factors of chronic kidney disease in the Thai adult population: Thai SEEK study. Nephrology, dialysis, transplantation : official publication of the European Dialysis and Transplant Association - European Renal Association. 2010;25(5):1567-75. Epub 2009/12/29. doi: 10.1093/ndt/gfp669. PubMed PMID: 20037182.
36. Ito J, Dung DTK, Vuong MT, Tuyen DG, Vinh LD, Huong NT, et al. Impact and perspective on chronic kidney disease in an Asian developing country: A large-scale survey in north Vietnam. Nephron Clin Pract. 2008;109(1):c25-c32. doi: 10.1159/000134379. PubMed Central PMCID: PMC18497502.
37. Jafar TH, Schmid CH, Levey AS. Serum creatinine as marker of kidney function in South Asians: A study of reduced GFR in adults in Pakistan. J Am Soc Nephrol. 2005;16(5):1413-9. doi: 10.1681/ASN.2004121100.

38. Jessani S, Bux R, Jafar TH. Prevalence, determinants, and management of chronic kidney disease in Karachi, Pakistan - A community based cross-sectional study. BMC Nephrol. 2014;15(1). doi: 10.1186/1471-2369-15-90. PubMed Central PMCID: PMC24927636.
39. Ji H, Zhang H, Xiong J, Yu S, Chi C, Bai B, et al. eGFRs from Asian-modified CKD-EPI and Chinese-modified CKD-EPI equations were associated better with hypertensive target organ damage in the community-dwelling elderly Chinese: The Northern Shanghai study. Clin Interventions Aging. 2017;12:1297-308. doi: 10.2147/CIA.S141102. PubMed Central PMCID: PMC28860731.
40. Ji A, Pan C, Wang H, Jin Z, Lee JH, Wu Q, et al. Prevalence and Associated Risk Factors of Chronic Kidney Disease in an Elderly Population from Eastern China. Int J Environ Res Public Health. 2019;16(22). Epub 2019/11/14. doi: 10.3390/ijerph16224383. PubMed PMID: 31717585; PubMed Central PMCID: PMCPMC6888049.
41. Jiang L, Liang Y, Qiu B, Wang F, Duan X, Yang X, et al. Metabolic syndrome and chronic kidney disease in a rural Chinese population. Clin Chim Acta. 2011;412(21-22):1983-8. doi: 10.1016/j.cca.2011.07.014. PubMed Central PMCID: PMC21801716.
42. Kaewput W, Thongprayoon C, Rangsin R, Ruangkanchanasetr P, Bathini T, Mao MA, et al. Association between serum uric acid and chronic kidney disease in patients with hypertension: A multicenter nationwide cross-sectional study. J Evid Based Med. 2019;12(4):235-42. Epub 2019/09/05. doi: 10.1111/jebm.12364. PubMed PMID: 31482688.
43. Khajehdehi P, Malekmakan L, Pakfetrat M, Roozbeh J, Sayadi M. Prevalence of Chronic Kidney Disease and Its Contributing Risk Factors in Southern Iran A Cross-sectional Adult Population-based Study. 2014.
44. Khanam P, Sayeed M, Islam A, Begum T, Habib S, Nahar N, et al. Hospital-based prevalence of chronic kidney disease among the newly registered patients with diabetes. 2016;7(3):2.
45. Kitiyakara C, Yamwong S, Vathesatogkit P, Chittamma A, Cheepudomwit S, Vanavanan S, et al. The impact of different GFR estimating equations on the prevalence of CKD and risk groups in a Southeast Asian cohort using the new KDIGO guidelines. BMC Nephrol. 2012;13(1). doi: 10.1186/1471-2369-13-1. PubMed Central PMCID: PMC22226403.
46. Kitiyakara C, Saranburut K, Thongmung N, Chittamma A, Vanavanan S, Donsakul K, et al. Long-term effects of socioeconomic status on the incidence of decreased glomerular filtration rate in a Southeast Asian cohort. Journal of Epidemiology and Community Health. 2020;74(11):925-32. doi: 10.1136/jech-2019-212718.
47. Le HT, Le TT, Tran NMT, Nguyen TTT, Minh NCS, Le QT, et al. Decreased glomerular filtration rate in patients with at least 5 years of type 2 diabetes in Ho Chi Minh City, Vietnam: Prevalence and associated factors. Primary Care Diabetes. 2020;14(2):173-80. doi: https://doi.org/10.1016/j.pcd.2019.08.003.
48. Li Y, Chen Y, Liu X, Liang Y, Shao X, Zhang Y, et al. Metabolic syndrome and chronic kidney disease in a Southern Chinese population. Nephrology. 2014;19(6):325-31. doi: 10.1111/nep.12219. PubMed Central PMCID: PMC24548104.
49. Li G, Huang J, Wang J, Zhao M, Liu Y, Guo X, et al. Long-Term Exposure to Ambient PM(2.5) and Increased Risk of CKD Prevalence in China. Journal of the American Society of Nephrology : JASN. 2021;32(2):448-58. Epub 2020/12/19. doi: 10.1681/asn.2020040517. PubMed PMID: 33334736; PubMed Central PMCID: PMCPMC8054885.

50. Liang Z, Wang W, Wang Y, Ma L, Liang C, Li P, et al. Urbanization, ambient air pollution, and prevalence of chronic kidney disease: A nationwide cross-sectional study. Environment international. 2021;156:106752. Epub 2021/07/14. doi: 10.1016/j.envint.2021.106752. PubMed PMID: 34256301.
51. Lin L, Lu J, Chen L, Mu Y, Ye Z, Liu C, et al. Glycemic status and chronic kidney disease in Chinese adults: Findings from the REACTION study. Journal of diabetes. 2017;9(9):837-45. Epub 2016/10/14. doi: 10.1111/1753-0407.12490. PubMed PMID: 27734593.
52. Liu K, Liu Q, Chen W, Liang M, Luo W, Wu X, et al. Prevalence and Risk Factors of CKD in Chinese Patients with Periodontal Disease. PLoS ONE. 2013;8(8). doi: 10.1371/journal.pone.0070767. PubMed Central PMCID: PMC23951003.
53. Liu Y, Yuan Y, Xiao Y, Li Y, Yu Y, Mo T, et al. Associations of plasma metal concentrations with the decline in kidney function: A longitudinal study of Chinese adults. Ecotoxicology and environmental safety. 2020;189:110006. doi: 10.1016/j.ecoenv.2019.110006.
54. Liu H, Zhou C, Yu J, Jiang Q, Hu D. Reduced kidney function in acute coronary syndrome patients with undiagnosed diabetes or pre-diabetes. Nephrology. 2013;18(4):263-8. doi: 10.1111/nep.12033. PubMed Central PMCID: PMC23332008.
55. Liu J, Chen Z, Li W, Xu G, Llu J, Yt B, et al. Obesity indices for prediction of chronic kidney disease: A cross-sectional study in 26 655 Chinese adults. J Cent South Univ Med Sci. 2016;41(5):445-54. doi: 10.11817/j.issn.l672-7347.2016.05.001. PubMed Central PMCID: PMC27269917.
56. Lu J, Mu Y, Su Q, Shi L, Liu C, Zhao J, et al. Reduced Kidney Function Is Associated With Cardiometabolic Risk Factors, Prevalent and Predicted Risk of Cardiovascular Disease in Chinese Adults: Results From the REACTION Study. J Am Heart Assoc. 2016;5(7). doi: 10.1161/JAHA.116.003328. PubMed Central PMCID: PMC27451464.
57. Luk AO, Li X, Zhang Y, Guo X, Jia W, Li W, et al. Quality of care in patients with diabetic kidney disease in Asia: The Joint Asia Diabetes Evaluation (JADE) Registry. Diabet Med. 2016;33(9):1230-9. doi: 10.1111/dme.13014. PubMed Central PMCID: PMC26511783.
58. Lv S, Shen Z, Zhang H, Yu X, Chen J, Gu Y, et al. Association between exposure to the Chinese famine during early life and the risk of chronic kidney disease in adulthood. Environ Res. 2020;184:109312. Epub 2020/03/11. doi: 10.1016/j.envres.2020.109312. PubMed PMID: 32151839.
59. Mahapatra HS, Gupta YP, Sharma N, Buxi G. Identification of high-risk population and prevalence of kidney damage among asymptomatic central government employees in Delhi, India. Saudi Journal of Kidney Diseases and Transplantation. 2016;27(2):362.
60. Mahdavi-Mazdeh M, Saeed Hashemi Nazri S, Hajghasemi E, Nozari B, Zinat Nadia H, Mahdavi A. Screening for decreased renal function in taxi drivers in Tehran, Iran. Renal failure. 2010;32(1):62-8.
61. Mohanty NK, Sahoo KC, Pati S, Sahu AK, Mohanty R. Prevalence of Chronic Kidney Disease in Cuttack District of Odisha, India. Int J Environ Res Public Health. 2020;17(2). Epub 2020/01/16. doi: 10.3390/ijerph17020456. PubMed PMID: 31936746; PubMed Central PMCID: PMCPMC7014305.
62. Naghibi M, Mojahedi MJ, Jarrahi L, Emadzadeh A, Ahmadi R, Emadzadeh M, et al. Prevalence of chronic kidney disease and its risk factors in Gonabad, Iran. Iranian Journal of Kidney Diseases. 2015;9(6):449.
63. Najafi I, Attari F, Islami F, Shakeri R, Malekzadeh F, Salahi R, et al. Renal function and risk factors of moderate to severe chronic kidney disease in Golestan Province, northeast of Iran. PLoS ONE. 2010;5(12):e14216.

64. Nampoothiri RV, Duseja A, Rathi M, Agrawal S, Sachdeva N, Mehta M, et al. Renal Dysfunction in Patients With Nonalcoholic Fatty Liver Disease is Related to the Presence of Diabetes Mellitus and Severity of Liver Disease. Journal of Clinical and Experimental Hepatology. 2019;9(1):22-8. doi: https://doi.org/10.1016/j.jceh.2017.12.005.
65. Nand N, Sharma M, Anand L, Shankar V, Kumar H. Evaluation of renal functions in patients having metabolic syndrome in Asian Indian cohort. J Indian Acad Clin Med. 2015;16(1):33-8.
66. Naseem R, Adam AM, Khan F, Dossal A, Khan I, Khan A, et al. Prevalence and characteristics of resistant hypertensive patients in an Asian population. Indian Heart Journal. 2017;69(4):442-6. doi: https://doi.org/10.1016/j.ihj.2017.01.012.
67. Ong-ajyooth L, Vareesangthip K, Khonputsa P, Aekplakorn W. Prevalence of chronic kidney disease in Thai adults: a national health survey. BioMed Central Ltd [Internet]. 2009. Available from: Item Resolution URL http://www.biomedcentral.com/1471-2369/10/35.
68. Pan Y, Zhang L, Wang F, Li X, Wang H, Group CNSoCKDW. Status of non-steroidal anti-inflammatory drugs use and its association with chronic kidney disease: A cross-sectional survey in C hina. Nephrology. 2014;19(10):655-60.
69. Pan L, Ma R, Wu Y, Feng L, Song Ys, Ye Xd, et al. Prevalence and risk factors associated with chronic kidney disease in a Z huang ethnic minority area in C hina. Nephrology. 2015;20(11):807-13.
70. Perkovic V, Cass A, Patel AA, Suriyawongpaisal P, Barzi F, Chadban S, et al. High prevalence of chronic kidney disease in Thailand. Kidney Int. 2008;73(4):473-9. Epub 2007/12/07. doi: 10.1038/sj.ki.5002701. PubMed PMID: 18059458.
71. Pongpirul W, Pongpirul K, Ananworanich J, Klinbuayaem V, Avihingsanon A, Prasithsirikul W. Chronic kidney disease incidence and survival of Thai HIV-infected patients. AIDS. 2018;32(3):393-8. doi: 10.1097/QAD.0000000000001698.
72. Prodjosudjadi W, Suhardjono, Suwitra K, Pranawa, Widiana IGR, Loekman JS, et al. Detection and prevention of chronic kidney disease in Indonesia: Initial community screening. Nephrology. 2009;14(7):669-74. doi: 10.1111/j.1440-1797.2009.01137.x. PubMed Central PMCID: PMC19796026.
73. Qiu Y, Zhao Q, Gu Y, Wang N, Yu Y, Wang R, et al. Association of Metabolic Syndrome and Its Components with Decreased Estimated Glomerular Filtration Rate in Adults. Annals of Nutrition and Metabolism. 2019;75(3):168-78. doi: 10.1159/000504356.
74. Rahim MA, Mitra P, Haque HF, Samdani TS, Zaman S, Uddin KNJIJoMS. Prevalence of chronic kidney disease stages 3-5 among patients with type 2 diabetes mellitus in Bangladesh. 2017;11(1):19-24.
75. Rai PK, Rai P, Bedi S. Prevalence and risk factors of chronic kidney disease in overweight and obese population in a tertiary care hospital in North India. Saudi J Kidney Dis Transpl. 2019;30(6):1431-8. Epub 2020/01/14. doi: 10.4103/1319-2442.275488. PubMed PMID: 31929291.
76. Rai PK, Rai P, Bhat RG, Bedi S. Chronic Kidney Disease among Middle-Aged and Elderly Population: A cross-sectional screening in a Hospital Camp in Varanasi, India. Saudi J Kidney Dis Transpl. 2019;30(4):795-802. Epub 2019/08/30. doi: 10.4103/1319-2442.265454. PubMed PMID: 31464235.
77. Rajput R, Kumar KP, Seshadri K, Agarwal P, Talwalkar P, Kotak B, et al. Prevalence of chronic kidney disease (CKD) in type 2 diabetes mellitus patients: START-India study. 2017;8(2):2-5.

78. Ruwanpathirana T, Senanayake S, Gunawardana N, Munasinghe A, Ginige S, Gamage D, et al. Prevalence and risk factors for impaired kidney function in the district of Anuradhapura, Sri Lanka: a cross-sectional population-representative survey in those at risk of chronic kidney disease of unknown aetiology. BMC Public Health. 2019;19(1):763. Epub 2019/06/16. doi: 10.1186/s12889-019-7117-2. PubMed PMID: 31200694; PubMed Central PMCID: PMCPMC6570843.
79. Saber A, Naghibzadeh Tahami A, Najafipour H, Azmandian J. Assessment of prevalence of chronic kidney disease and its predisposing factors in Kerman city. Nephro-Urology Monthly. 2017;9(2).
80. Sahin I, Yildirim B, Cetin I, Etikan I, Ozturk B, Ozyurt H, et al. Prevalence of chronic kidney disease in the Black Sea Region, Turkey, and investigation of the related factors with chronic kidney disease. Renal failure. 2009;31(10):920-7.
81. Satirapoj B, Supasyndh O, Mayteedol N, Punpanich D, Chaiprasert A, Nata N, et al. Obesity and its relation to chronic kidney disease: A population-based, cross-sectional study of a T hai army population and relatives. Nephrology. 2013;18(3):229-34.
82. Sepanlou SG, Barahimi H, Najafi I, Kamangar F, Poustchi H, Shakeri R, et al. Prevalence and determinants of chronic kidney disease in northeast of Iran: Results of the Golestan cohort study. PLoS ONE. 2017;12(5):e0176540.
83. Shan Y, Zhang Q, Liu Z, Hu X, Liu D. Prevalence and risk factors associated with chronic kidney disease in adults over 40 years: A population study from Central China. Nephrology. 2010;15(3):354-61. doi: 10.1111/j.1440-1797.2009.01249.x. PubMed Central PMCID: PMC20470307.
84. Sharma SK, Zou H, Togtokh A, Ene-Iordache B, Carminati S, Remuzzi A, et al. Burden of CKD, proteinuria, and cardiovascular risk among Chinese, Mongolian, and Nepalese participants in the International Society of Nephrology screening programs. 2010;56(5):915-27.
85. Sharma SK, Dhakal S, Thapa L, Ghimire A, Tamrakar R, Chaudhary S, et al. Community-based screening for chronic kidney disease, hypertension and diabetes in Dharan. 2013.
86. Shen Q, Jin W, Ji S, Chen X, Zhao X, Behera TR. The association between socioeconomic status and prevalence of chronic kidney disease: A cross-sectional study among rural residents in eastern China. Medicine. 2019;98(11):e14822. Epub 2019/03/19. doi: 10.1097/md.0000000000014822. PubMed PMID: 30882664; PubMed Central PMCID: PMCPMC6426623.
87. Shi Z, Zhang M, Liu J. Chili Intake Is Inversely Associated with Chronic Kidney Disease among Adults: A Population-Based Study. Nutrients. 2019;11(12). Epub 2019/12/11. doi: 10.3390/nu11122949. PubMed PMID: 31817083; PubMed Central PMCID: PMCPMC6949978.
88. Shi W, Wang H, Zhou Y, Sun Y, Chen Y. Synergistic interaction of hyperuricemia and hypertension on reduced eGFR: insights from a general Chinese population. Postgraduate medicine. 2020;132(3):263-9. doi: 10.1080/00325481.2020.1718387.
89. Singh NP, Ingle GK, Saini VK, Jami A, Beniwal P, Lal M, et al. Prevalence of low glomerular filtration rate, proteinuria and associated risk factors in North India using Cockcroft-Gault and Modification of Diet in Renal Disease equation: An observational, cross-sectional study. BMC Nephrol. 2009;10(1). doi: 10.1186/1471-2369-10-4. PubMed Central PMCID: PMC19220921.
90. Singh AK, Farag YMK, Mittal BV, Subramanian KK, Reddy SRK, Acharya VN, et al. Epidemiology and risk factors of chronic kidney disease in India - Results from the SEEK (Screening and Early Evaluation of Kidney Disease) study. BMC Nephrol. 2013;14(1). doi: 10.1186/1471-2369-14-114

10.1016/j.dsx.2012.05.016. Epub 2012 10.1016/j.dsx.2012.05.016 23153977; Devi, P., Rao, M., Sigamani, A., Prevalence, risk factors and awareness of hypertension in India: A systematic review (2012) J Hum Hypertens, , doi:10.1038/jhh.2012.33; Du, X., Hu, B., Jiang, L., Wan, X., Fan, L., Wang, F., Implication of CKD-EPI equation to estimate glomerular filtration rate in Chinese patients with chronic kidney disease (2011) Ren Fail, 33 (9), pp. 859-865. , 10.3109/0886022X.2011.605533 21851197; Gifford, F.J., Methven, S., Boag, D.E., Spalding, E.M., Macgregor, M.S., Chronic kidney disease prevalence and secular trends in a UK population: The impact of MDRD and CKD-EPI formulae (2011) QJM, 104 (12), pp. 1045-1053. , 10.1093/qjmed/hcr122 21821654; Stevens, L.A., Li, S., Kurella Tamura, M., Chen, S.C., Vassalotti, J.A., Norris, K.C., Comparison of the CKD epidemiology collaboration (CKD-EPI) and modification of diet in renal disease (MDRD) study equations: Risk factors for and complications of CKD and mortality in the kidney early evaluation program (KEEP) (2011) Am J Kidney Dis, 57 (SUPPL 2), pp. 199-216. , 21338849; McFarlane, S.I., McCullough, P.A., Sowers, J.R., Soe, K., Chen, S.C., Li, S., Comparison of the CKD epidemiology collaboration (CKD-EPI) and modification of diet in renal disease (MDRD) study equations: Prevalence of and risk factors for diabetes mellitus in CKD in the kidney early evaluation program (KEEP) (2011) Am J Kidney Dis, 57 (SUPPL 2), pp. 1924-1931. , 21338847.

91. Song J, Wang C, Ma A, Zheng H, Zheng W, Hou X, et al. Self-reported snoring is associated with chronic kidney disease independent of metabolic syndrome in middle-aged and elderly Chinese. Journal of Diabetes Investigation. 2019;10(1):124-30. doi: 10.1111/jdi.12855.
92. Sun X, He J, Ji X-L, Zhao Y-M, Lou H-Y, Song X-X, et al. Association of chronic kidney disease with coronary heart disease and stroke risks in patients with type 2 diabetes mellitus: an observational cross-sectional study in Hangzhou, China. Chin Med J. 2017;130(1):57-63.
93. Tatapudi RR, Rentala S, Gullipalli P, Komarraju AL, Singh AK, Tatapudi VS, et al. High prevalence of CKD of unknown etiology in Uddanam, India. 2019;4(3):380-9.
94. Trivedi H, Vanikar A, Patel H, Kanodia K, Kute V, Nigam L, et al. High prevalence of chronic kidney disease in a semi-urban population of Western India. Clinical kidney journal. 2016;9(3):438-43.
95. Varma PP, Raman DK, Ramakrishnan T, Singh P, Varma A. Prevalence of early stages of chronic kidney disease in apparently healthy central government employees in India. Nephrology Dialysis Transplantation. 2010;25(9):3011-7.
96. Varma P, Raman D, Ramakrishnan T, Singh P. Prevalence of early stages of chronic kidney disease in healthy Army personnel. Medical Journal Armed Forces India. 2011;67(1):9-14.
97. Wang S, Chen R, Liu Q, Shu Z, Zhan S, Li L. Prevalence, awareness and treatment of chronic kidney disease among middle-aged and elderly: The China Health and Retirement Longitudinal Study. Nephrology. 2015;20(7):474-84. doi: 10.1111/nep.12449. PubMed Central PMCID: PMC25773384.
98. Wang Q, Xie D, Xu X, Qin X, Tang G, Wang B, et al. Blood pressure and renal function decline: A 7-year prospective cohort study in middle-aged rural Chinese men and women. J Hypertens. 2015;33(1):136-43. doi: 10.1097/HJH.0000000000000360. PubMed Central PMCID: PMC25255396.
99. Wang F, He K, Wang J, Zhao M-H, Li Y, Zhang L, et al. Prevalence and Risk Factors for CKD: A Comparison Between the Adult Populations in China and the United States. Kidney International Reports. 2018;3(5):1135-43. doi: https://doi.org/10.1016/j.ekir.2018.05.011.

100. Wen J, Chen Y, Huang Y, Lu Y, Liu X, Zhou H, et al. Association of the TG/HDL-C and non-HDL-C/HDL-C ratios with chronic kidney disease in an adult Chinese population. Kidney and Blood Pressure Research. 2017;42(6):1141-54.
101. Xu SS, Hua J, Huang YQ, Shu L. Association between dietary patterns and chronic kidney disease in a middle-aged Chinese population. Public Health Nutrition. 2020;23(6):1058-66. doi: 10.1017/S1368980019002805.
102. Yang S, Xiao F, Pan L, Zhang H, Ma Z, Liu S, et al. Association of serum irisin and body composition with chronic kidney disease in obese Chinese adults: A cross-sectional study. BMC Nephrol. 2015;16(1). doi: 10.1186/s12882-015-0009-5. PubMed Central PMCID: PMC25884312.
103. Yang S, Li M, Chen Y, Zhao X, Chen X, Wang H, et al. Comparison of the Correlates Between Body Mass Index, Waist Circumference, Waist-to-Height Ratio, and Chronic Kidney Disease in a Rural Chinese Adult Population. J Ren Nutr. 2019;29(4):302-9.e1. Epub 2018/12/26. doi: 10.1053/j.jrn.2018.10.008. PubMed PMID: 30581064.
104. Yang Y, Zhou W, Wang Y, Zhou R. Gender-specific association between uric acid level and chronic kidney disease in the elderly health checkup population in China. Ren Fail. 2019;41(1):197-203. Epub 2019/04/12. doi: 10.1080/0886022x.2019.1591994. PubMed PMID: 30973288; PubMed Central PMCID: PMCPMC6461085.
105. Yang Y, Wang N, Jiang Y, Zhao Q, Chen Y, Ying X, et al. The prevalence of diabetes mellitus with chronic kidney disease in adults and associated factors in Songjiang District, Shanghai. Annals of palliative medicine. 2021;10(7):7214-24. Epub 2021/07/16. doi: 10.21037/apm-21-803. PubMed PMID: 34263637.
106. Yuzbashian E, Asghari G, Mirmiran P, Zadeh-Vakili A, Azizi F. Sugar-sweetened beverage consumption and risk of incident chronic kidney disease: Tehran lipid and glucose study. Nephrology. 2016;21(7):608-16. doi: 10.1111/nep.12646. PubMed Central PMCID: PMC26439668.
107. Zaman SB, Karim MA, Hossain N, Al Kibria GM, Islam SMS. Plasma triglycerides as a risk factor for chronic kidney disease in type 2 diabetes mellitus: Evidence from northeastern Thailand. Diabetes Research and Clinical Practice. 2018;138:238-45. doi: https://doi.org/10.1016/j.diabres.2018.02.011.
108. Zhang C, Zhong Y, Tian H. Increased cardio-ankle vascular index is independently associated with chronic kidney disease: A cross-sectional study in Chinese patients with type 2 diabetes mellitus. J Diabetes Complications. 2019;33(9):623-7. Epub 2019/07/01. doi: 10.1016/j.jdiacomp.2019.05.009. PubMed PMID: 31255433.
109. Zhang M, Wang K, Zheng H, Zhao X, Xie S, Liu C. Monocyte lymphocyte ratio predicts the new-onset of chronic kidney disease: A cohort study. Clin Chim Acta. 2020;503:181-9. doi: 10.1016/j.cca.2019.11.021.
110. Zhang Z, Cui T, Cui M, Kong X. High prevalence of chronic kidney disease among patients with diabetic foot: A cross-sectional study at a tertiary hospital in China. Nephrology (Carlton). 2020;25(2):150-5. Epub 2019/04/27. doi: 10.1111/nep.13596. PubMed PMID: 31025471.
