# Supplementary material for: Prevalence of chronic kidney disease stages 3–5 in low- and middle-income countries in Asia: A systematic review and meta-analysis
Source: PLoS One. 2022 Feb 25;17(2):e0264393. doi: 10.1371/journal.pone.0264393 (PMC8880400; doi:10.1371/journal.pone.0264393)

S6 Appendix. Other forest plots in chronic kidney disease prevalence and associated risk factors.

Abbreviations:

CKD, chronic kidney disease; LMICs, low- and middle- income counties; LDLc, low-density lipoprotein cholesterol; HDLc, high-density lipoprotein cholesterol; HT, hypertension; HIV, human immunodeficiency viruses

Fig S1. Meta-analysis of chronic kidney disease prevalence in LMICs in Asia stratified by quality of study.

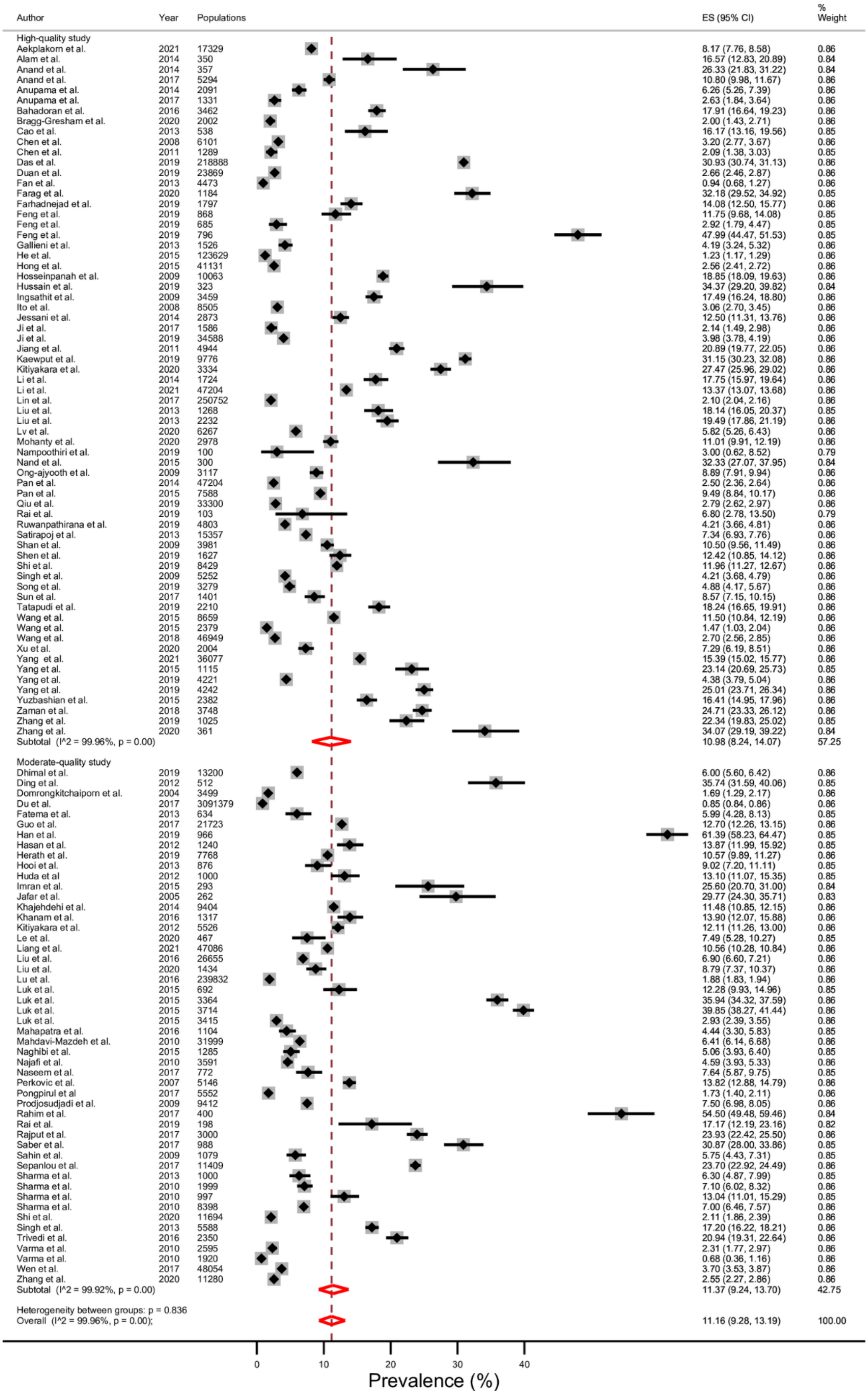

Fig S2. Meta-analysis of chronic kidney disease prevalence in LMICs in Asia stratified by study design.

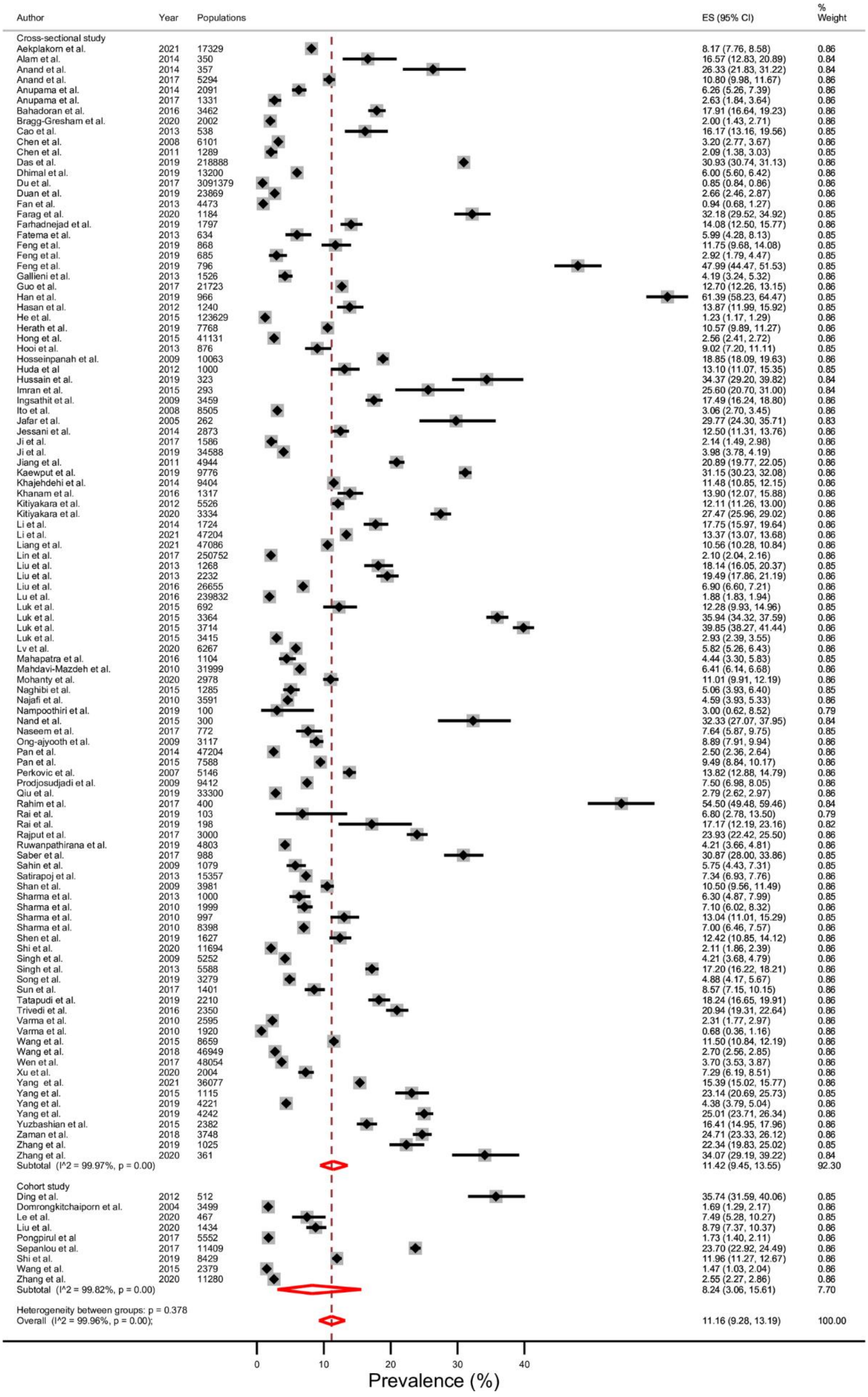

Fig S3. Meta-analysis of chronic kidney disease prevalence in LMICs in Asia stratified by classification of CKD (methods for measurement of eGFR).

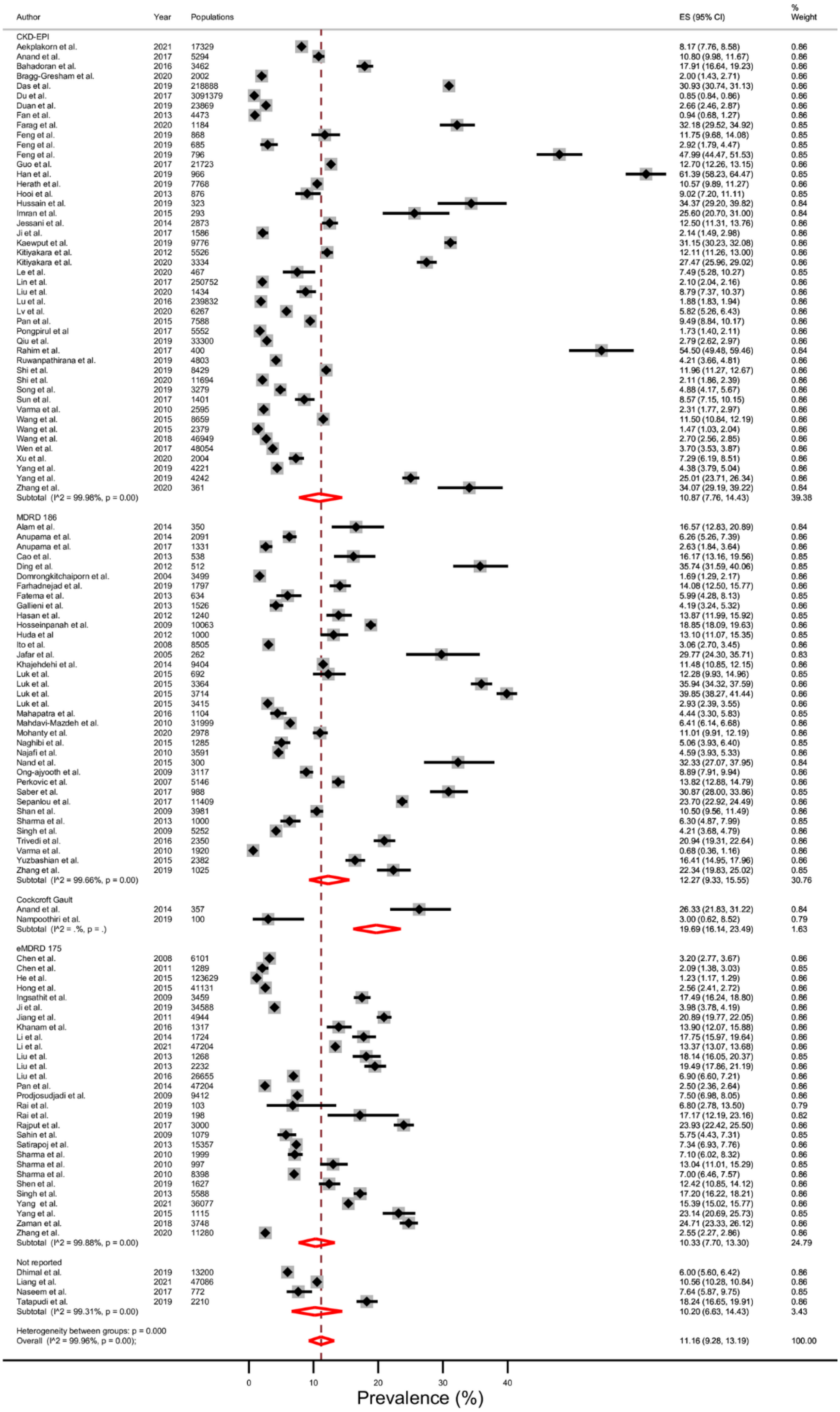

Fig S4. Meta-analysis of chronic kidney disease prevalence in LMICs in Asia stratified by economic group.

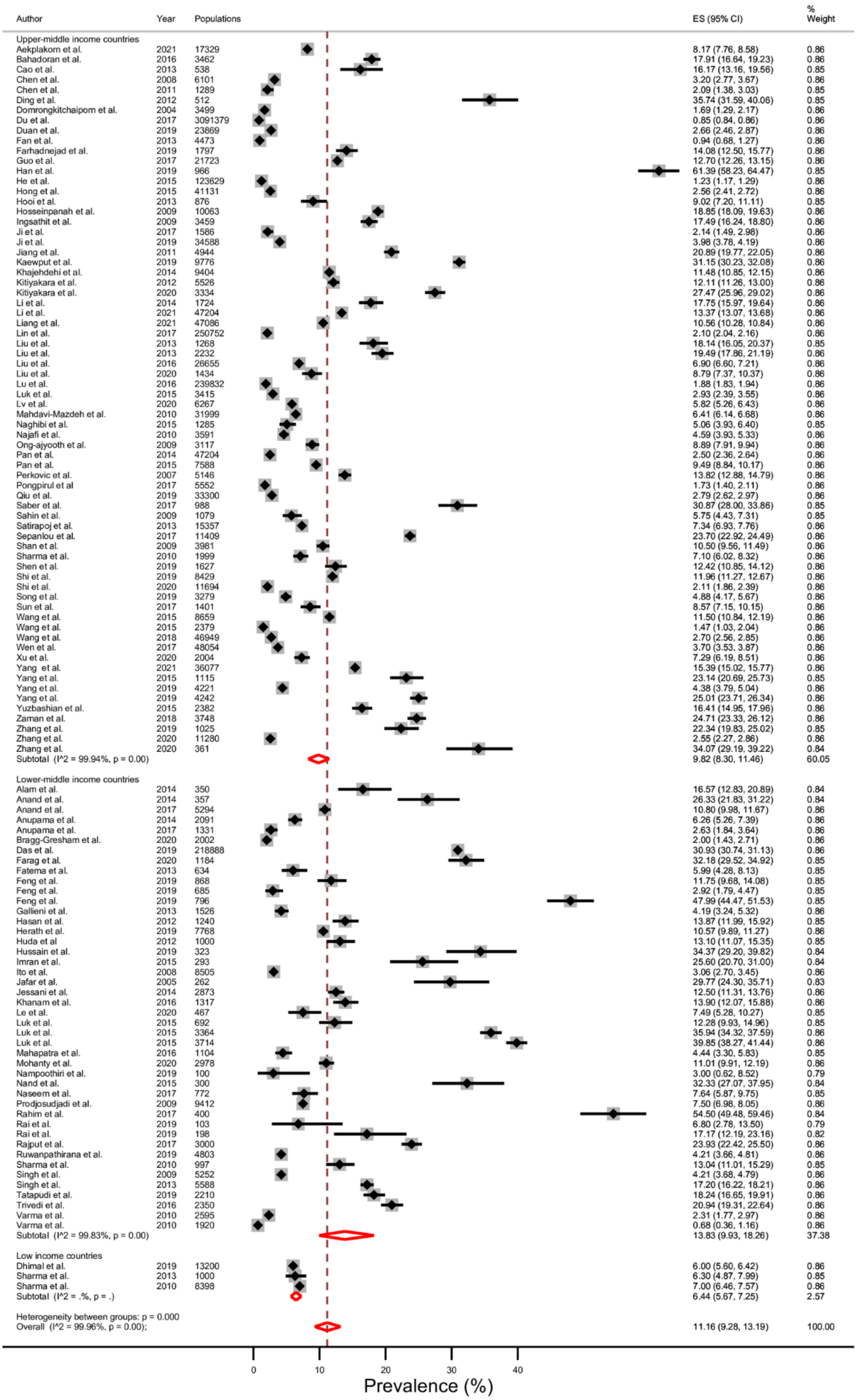

Fig S5. Meta-analysis of association between chronic kidney disease and elderly age (age ≥60 years or <60 years).

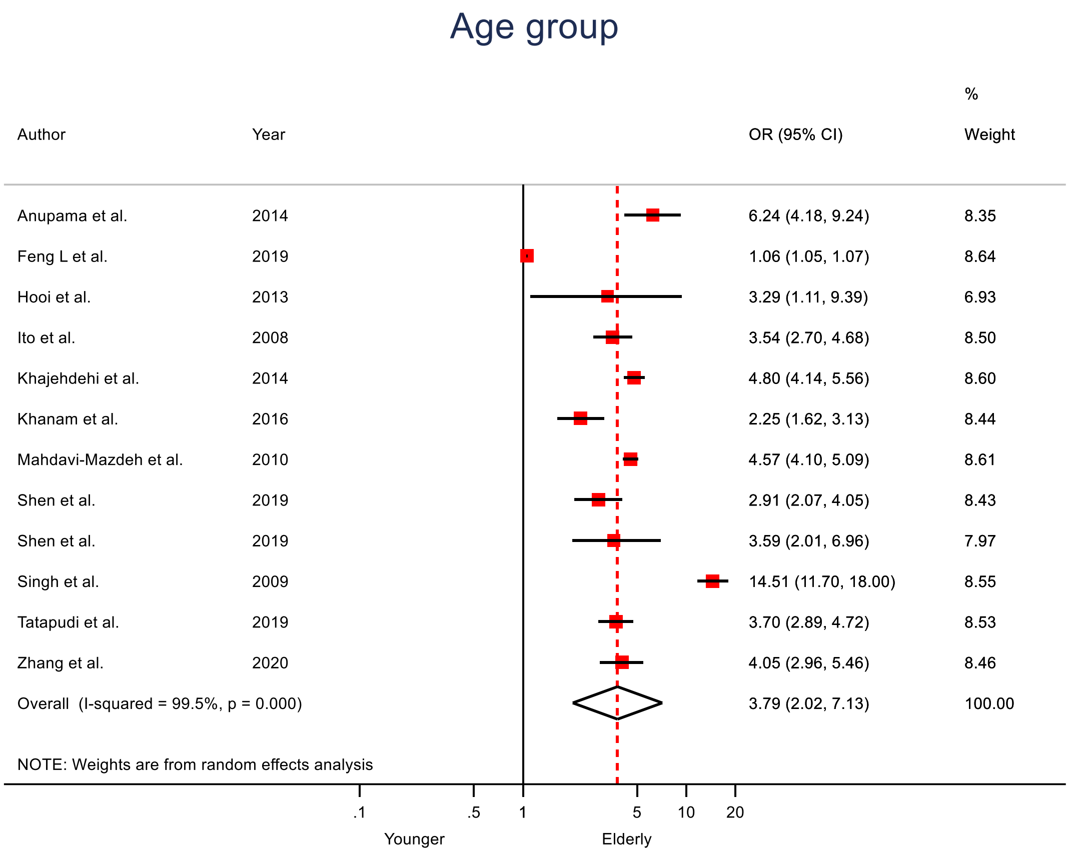

Fig S6. Meta-analysis of association between chronic kidney disease and gender (male or female).

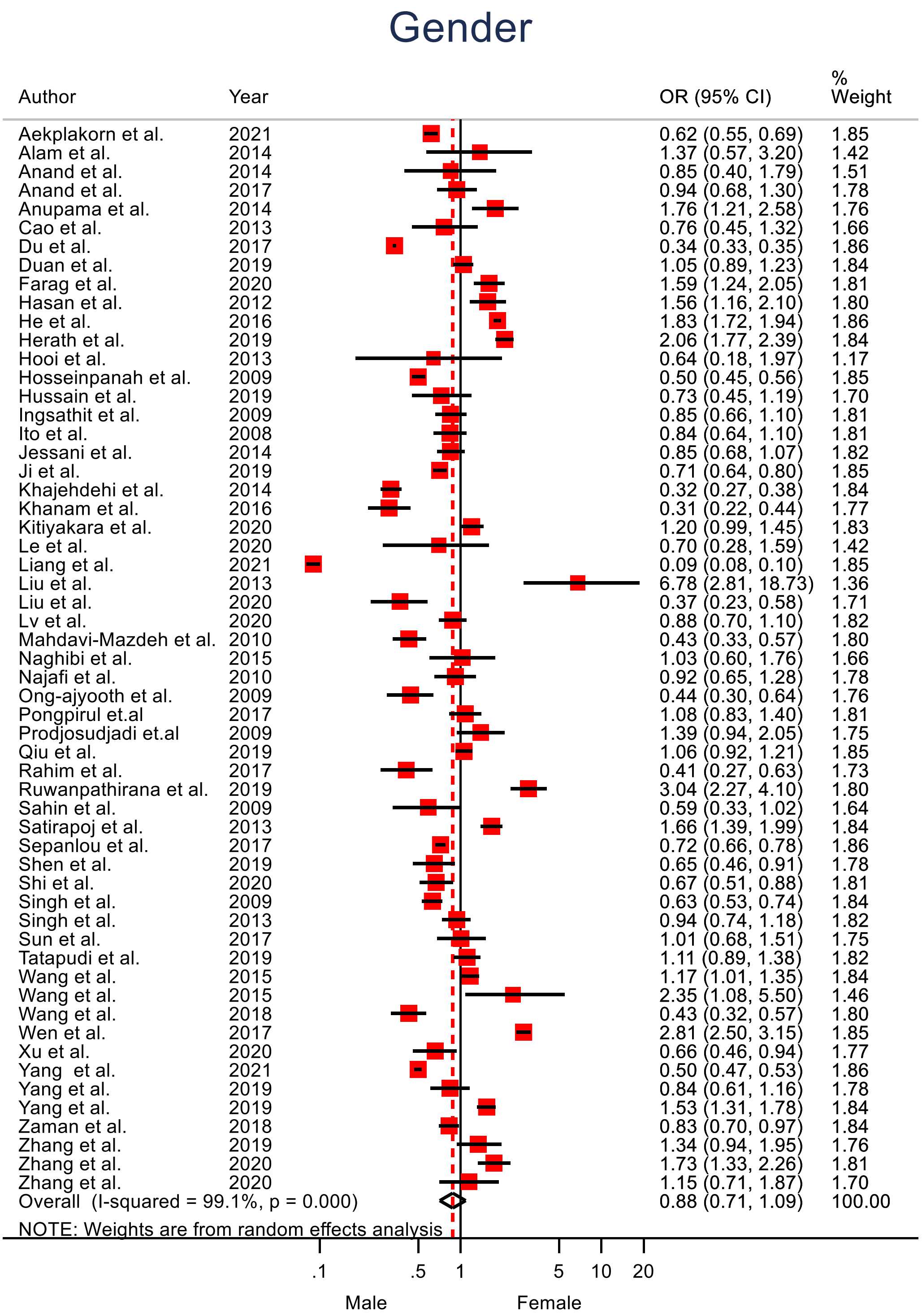

Fig S7. Meta-analysis of association between chronic kidney disease and obese (BMI ≥25 or 18-25 kg/m^2).

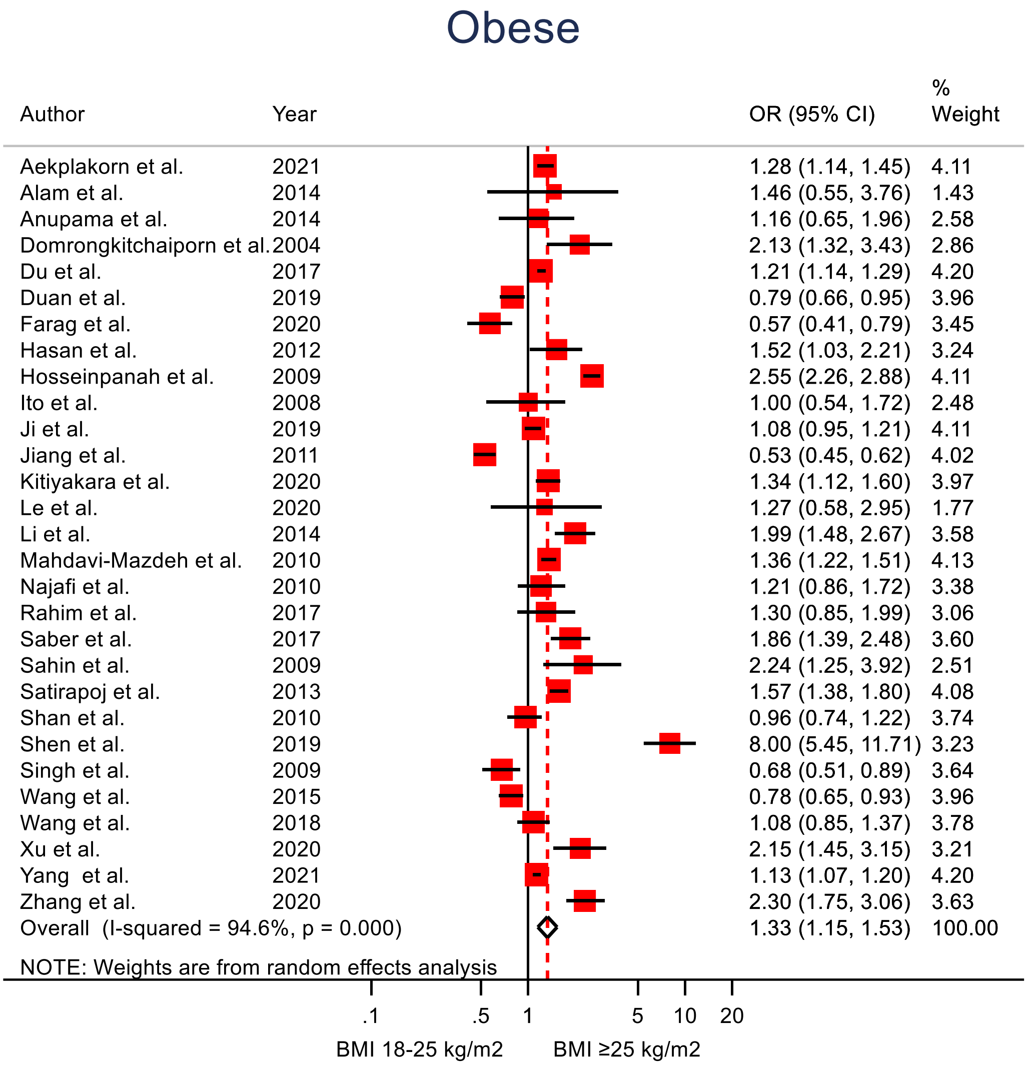

Fig S8. Meta-analysis of association between chronic kidney disease and lower weight (BMI <18 or 18-25 kg/m^2).

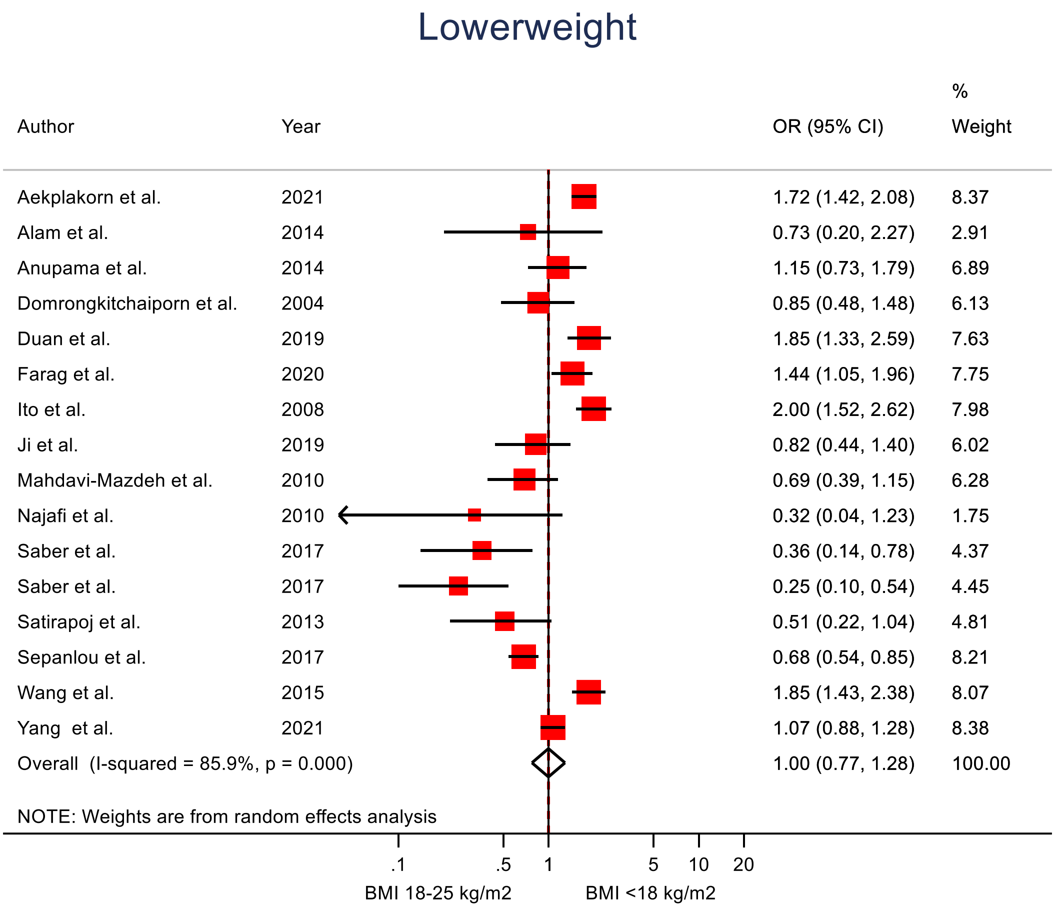

Fig S9. Meta-analysis of association between chronic kidney disease and hypertension (yes or no).

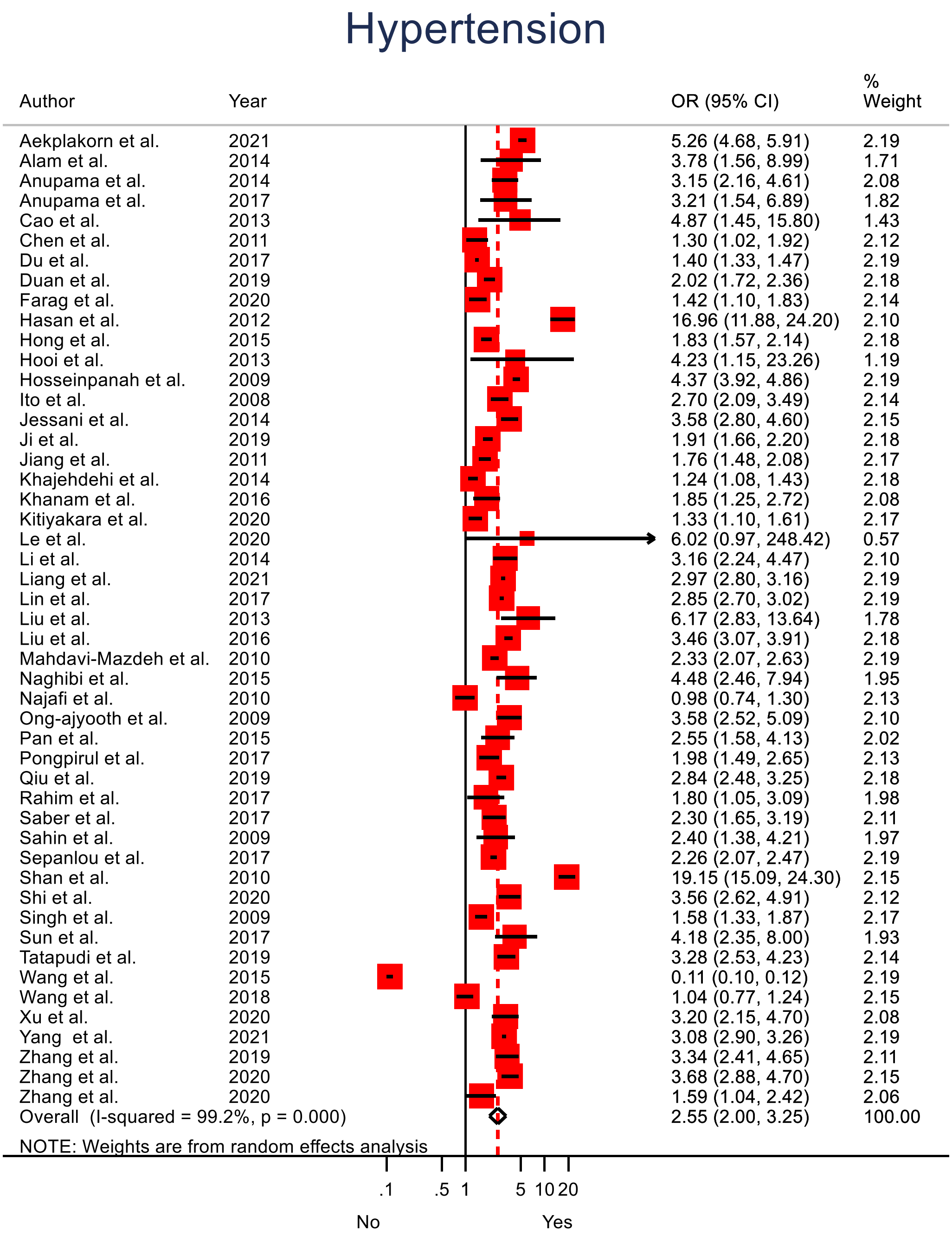

Fig S10. Meta-analysis of association between chronic kidney disease and diabetes (yes or no).

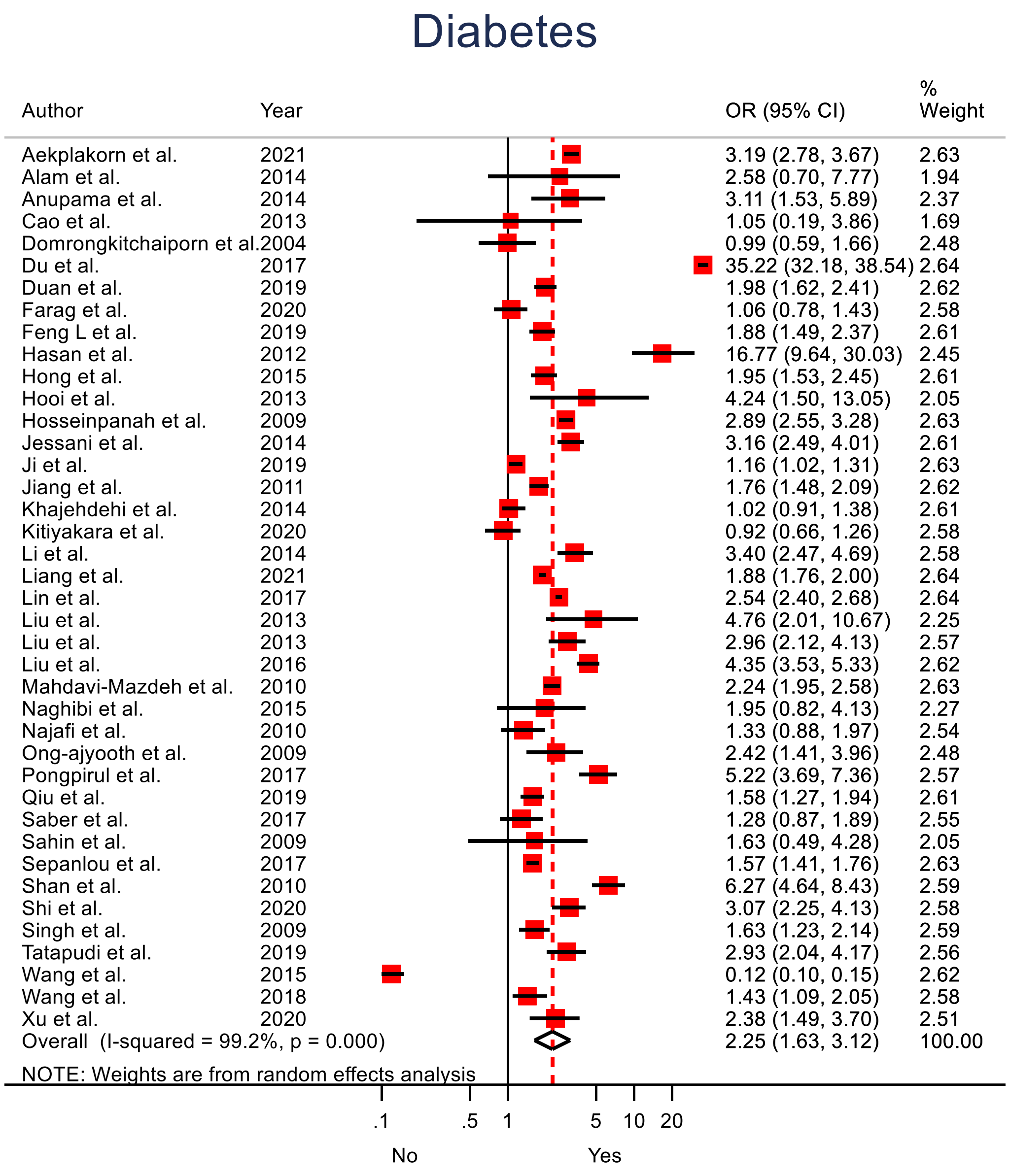

Fig S11. Meta-analysis of association between chronic kidney disease and dyslipidaemia (yes or no).

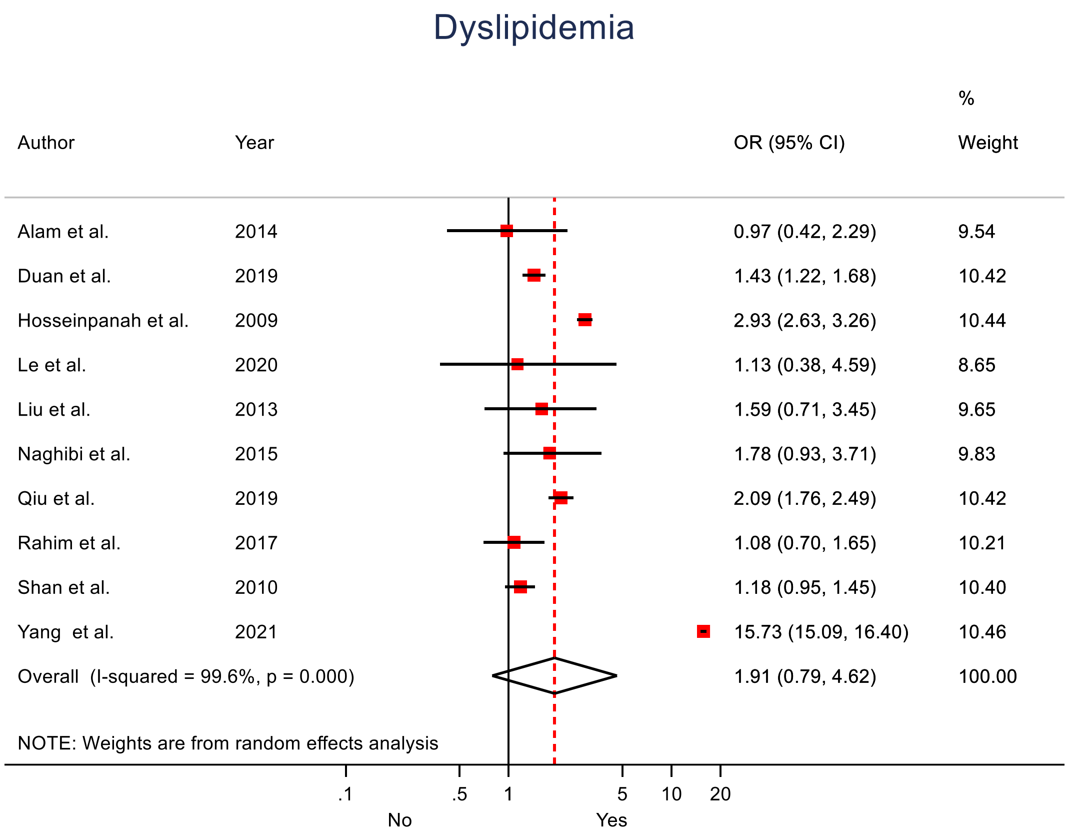

Fig S12. Meta-analysis of association between chronic kidney disease and Hypertriglyceridemia (yes or no).

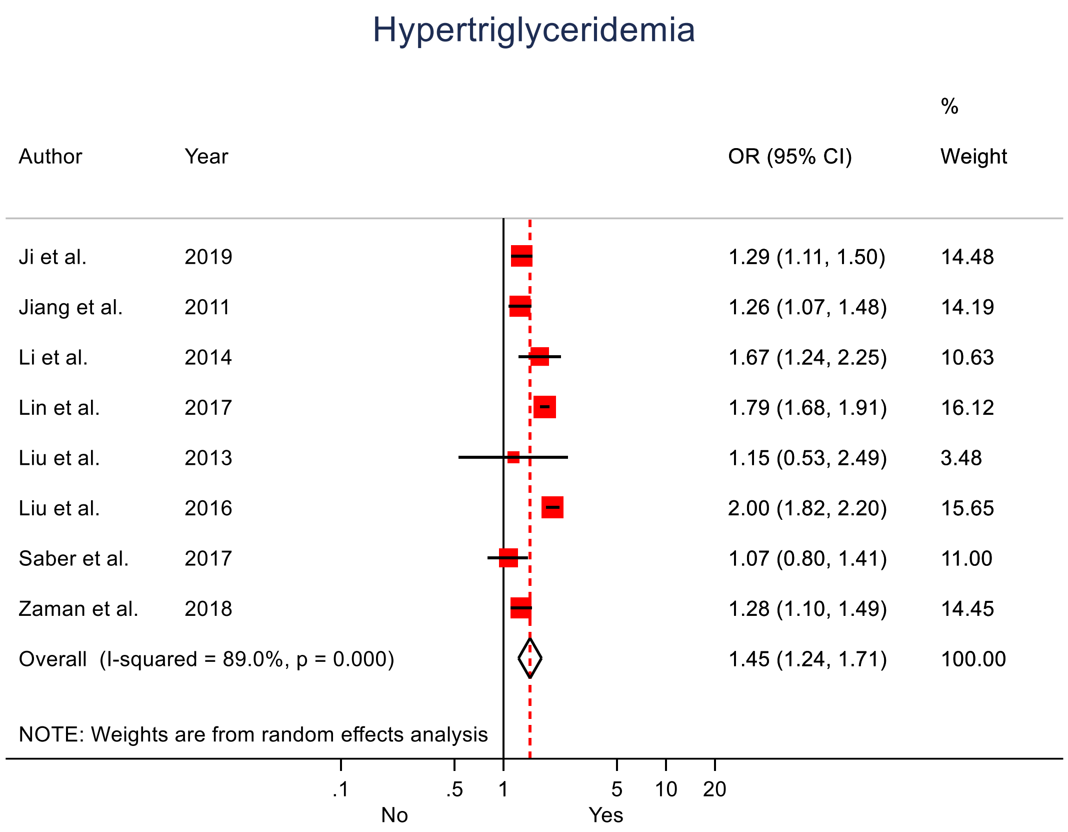

Fig S13. Meta-analysis of association between chronic kidney disease and Hypercholesterolaemia (yes or no).

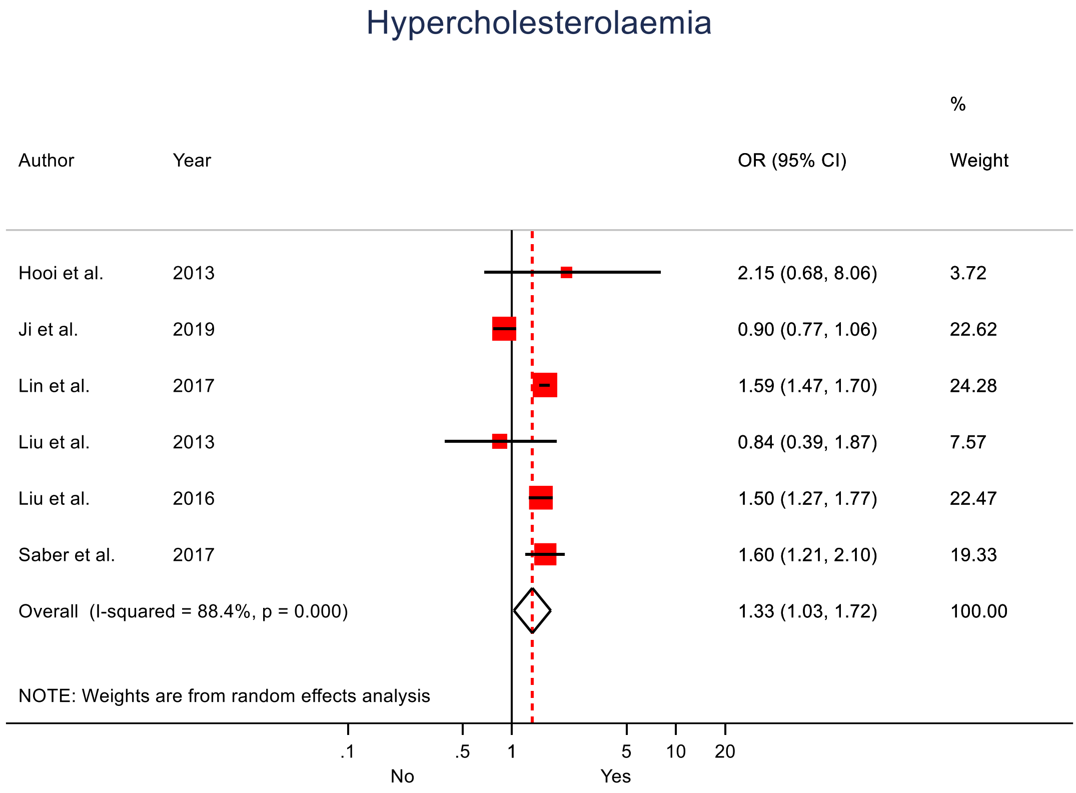

Fig S14. Meta-analysis of association between chronic kidney disease and high level of low-density lipoprotein cholesterol (yes or no).

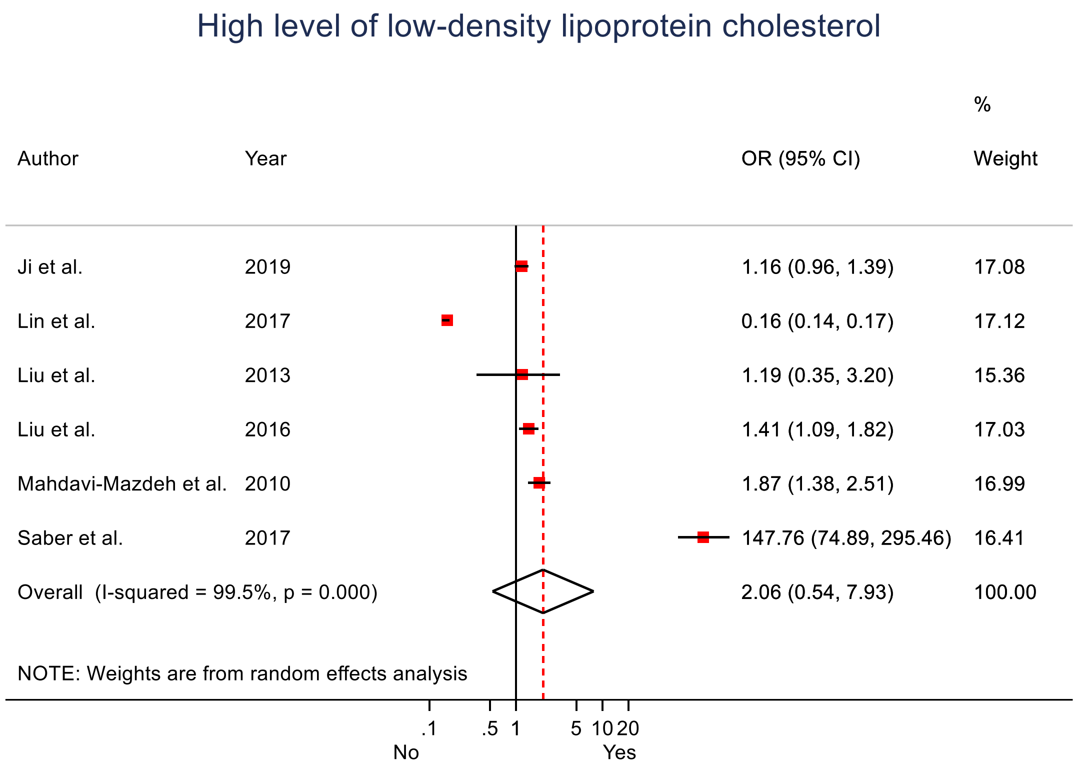

Fig S15. Meta-analysis of association between chronic kidney disease and low level of high-density lipoprotein cholesterol (yes or no).

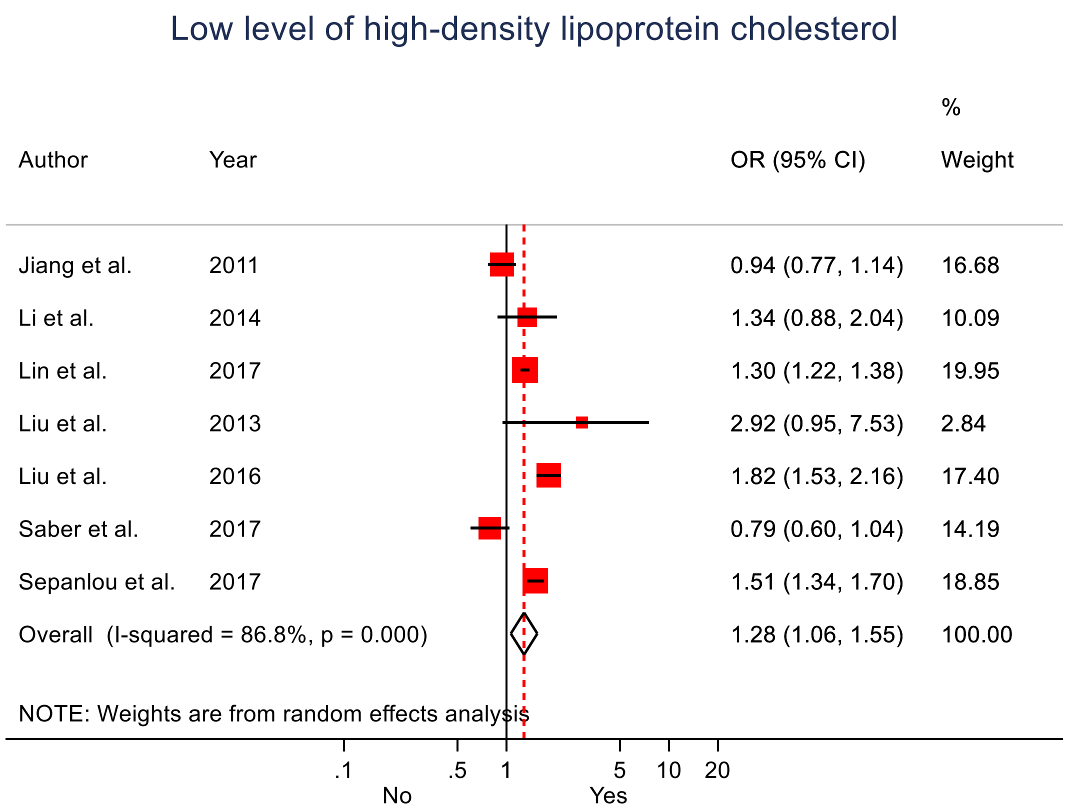

Fig S16. Meta-analysis of association between chronic kidney disease and history of coronary heart disease (yes or no).

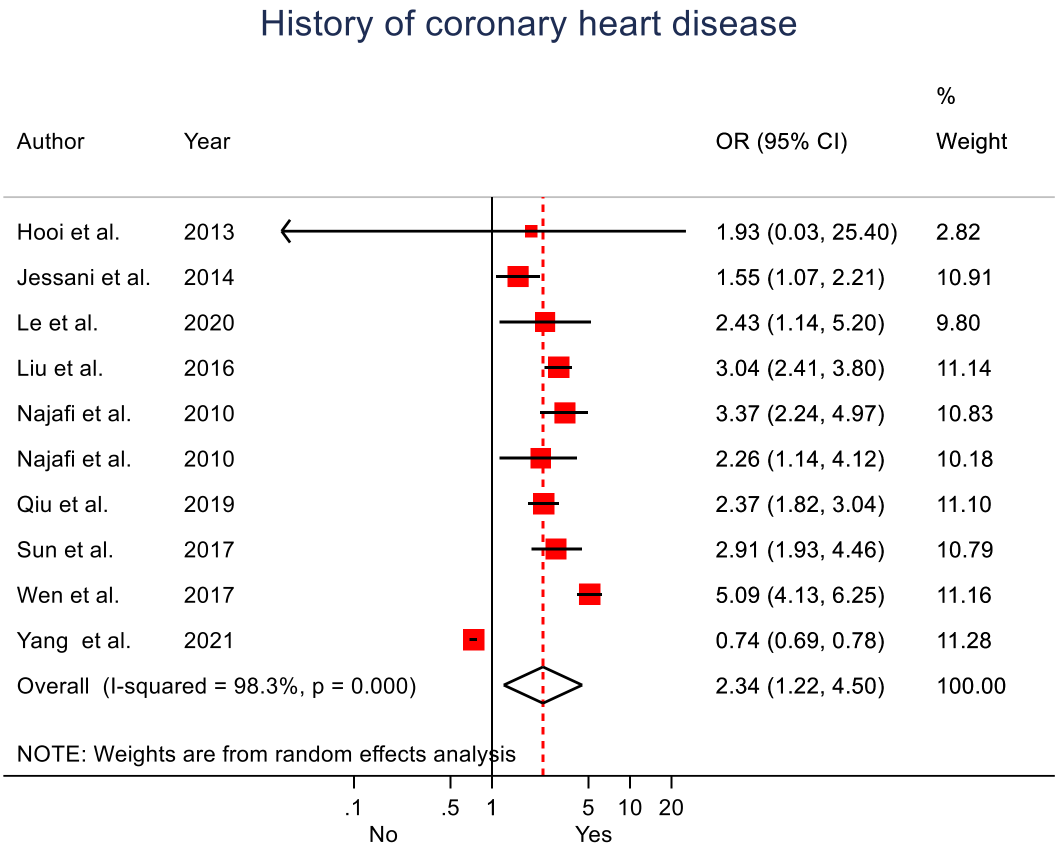

Fig S17. Meta-analysis of association between chronic kidney disease and history of stroke (yes or no).

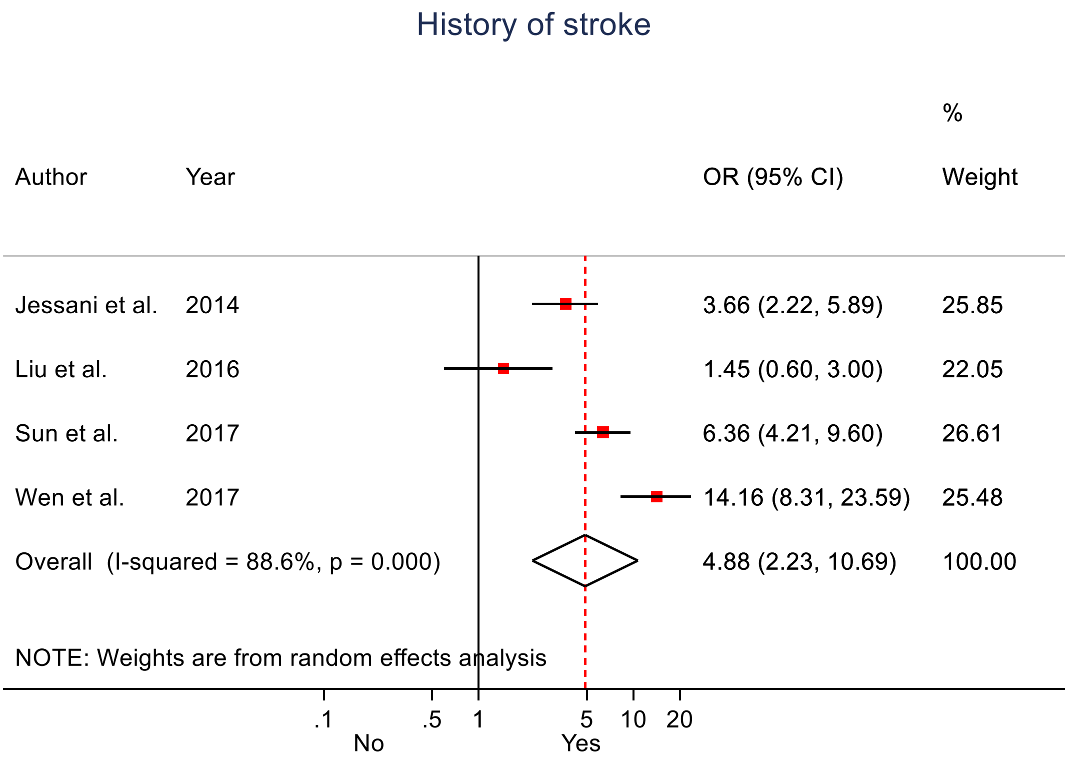

Fig S18. Meta-analysis of association between chronic kidney disease and history of cardiovascular disease (yes or no).

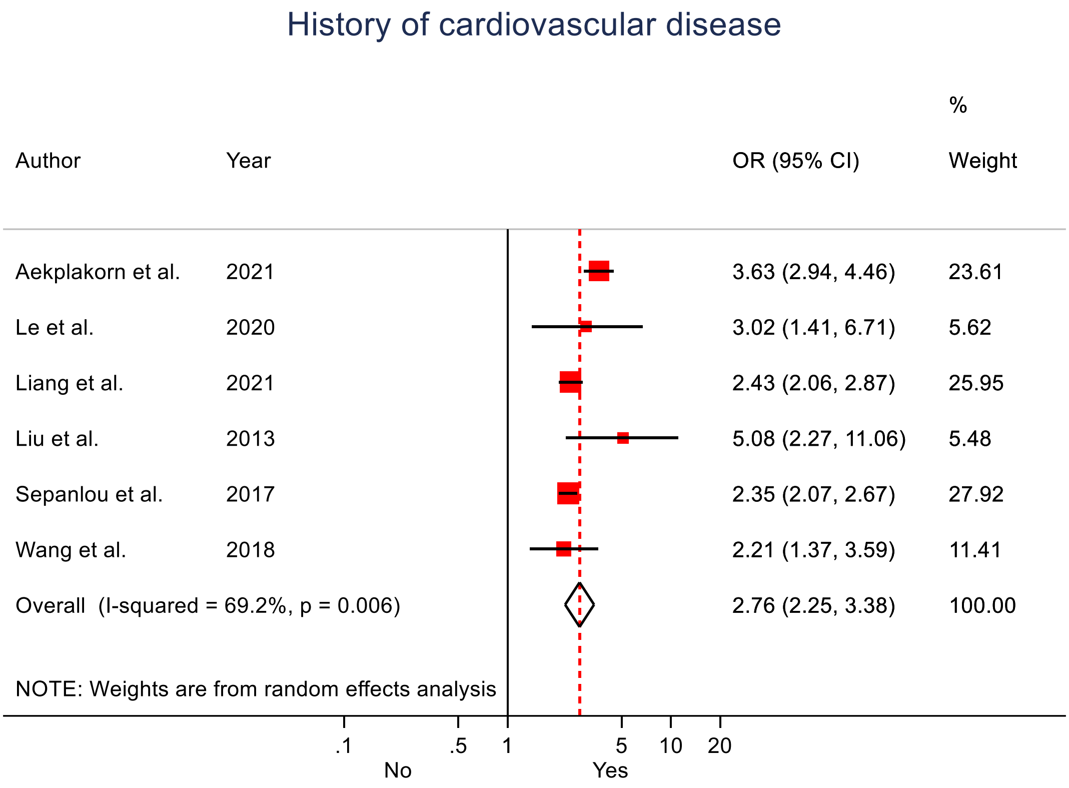

Fig S19. Meta-analysis of association between chronic kidney disease and education (< high school or ≥ high school).

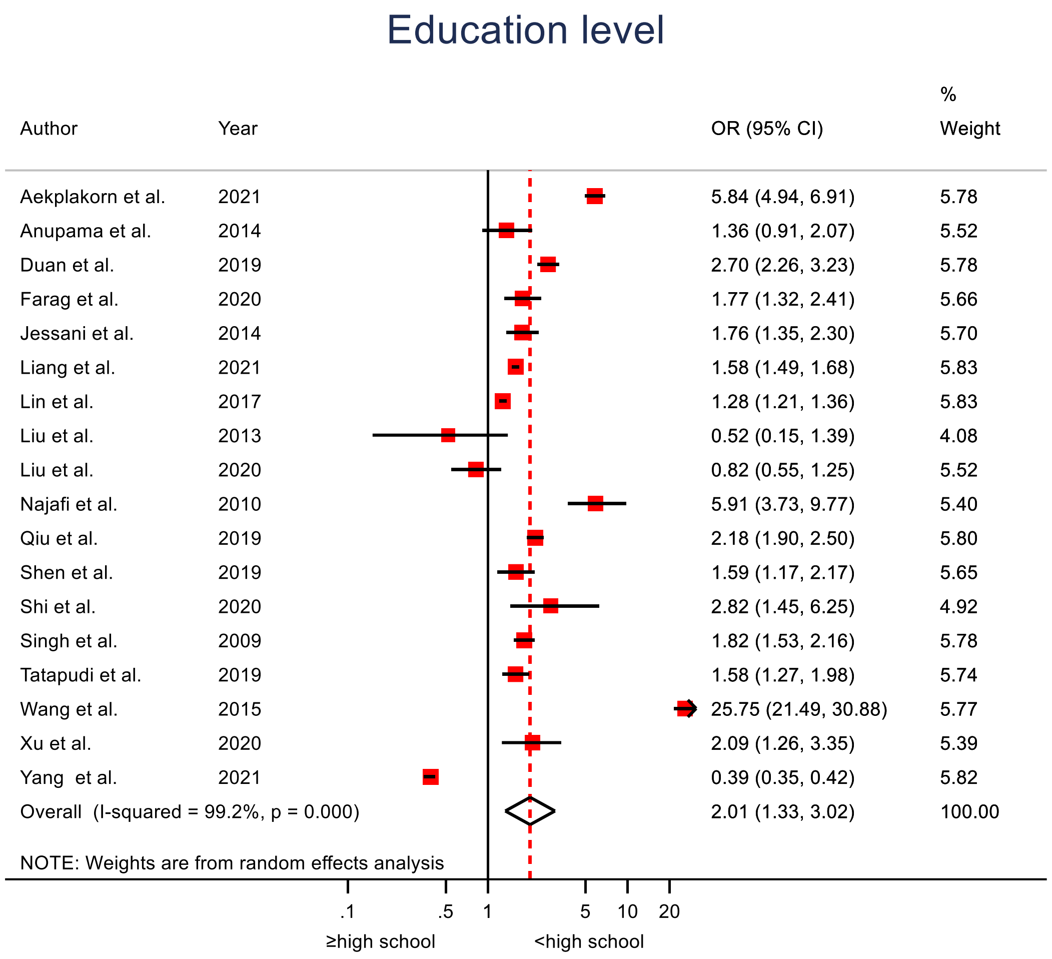

Fig S20. Meta-analysis of association between chronic kidney disease and marital status (being unmarried or married).

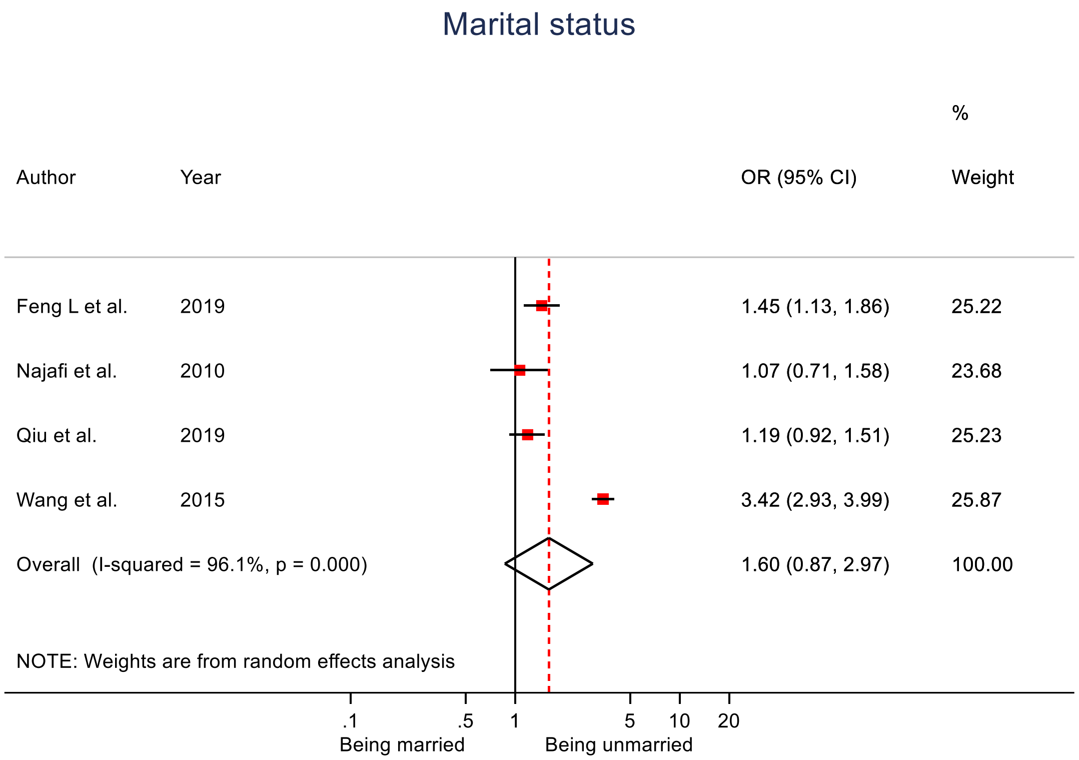

Fig S21. Meta-analysis of association between chronic kidney disease and hyperuricaemia (yes or no).

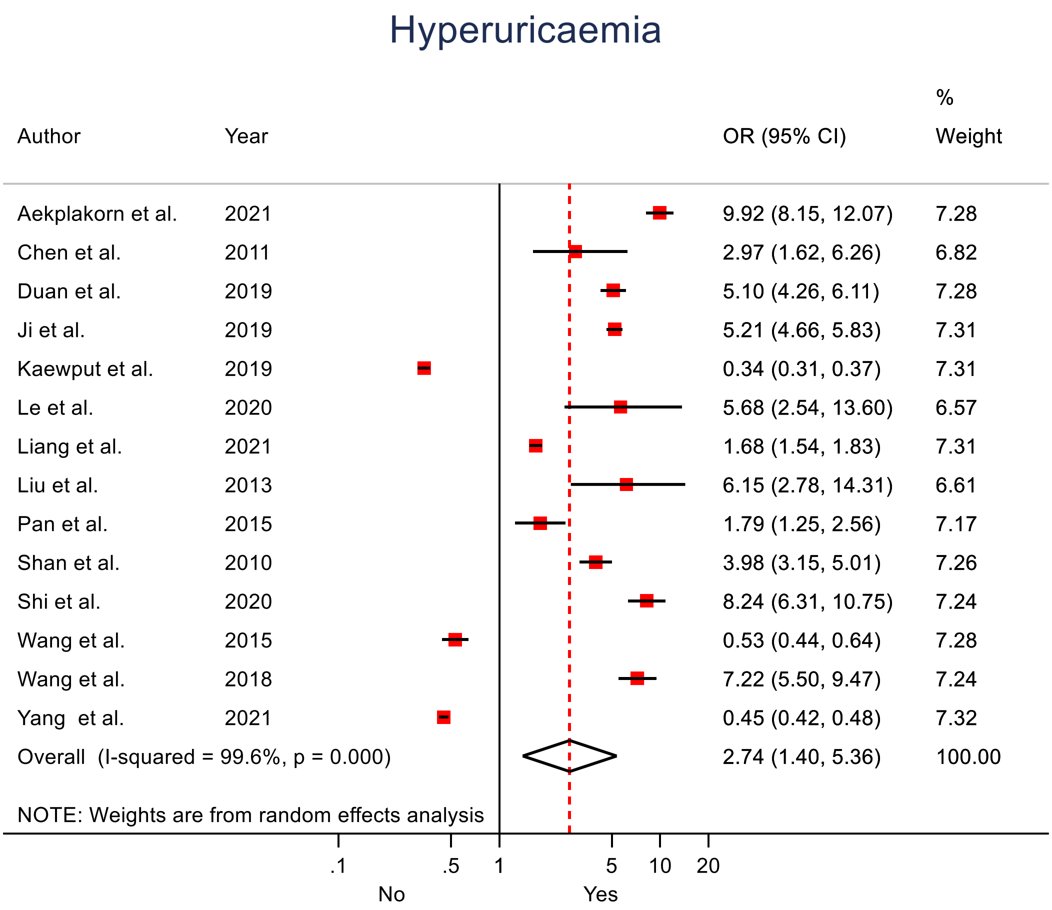

Fig S22. Meta-analysis of association between chronic kidney disease and anemia (yes or no).

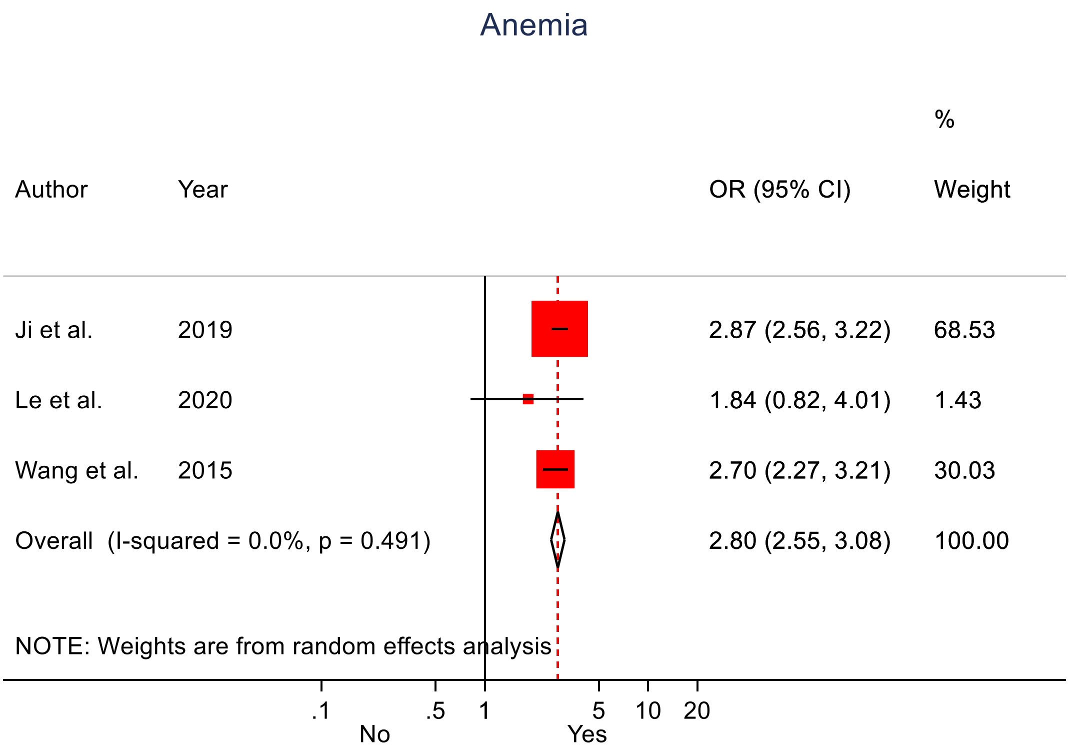

Fig S23. Meta-analysis of association between chronic kidney disease and smoking status (current smoker or non-smoker).

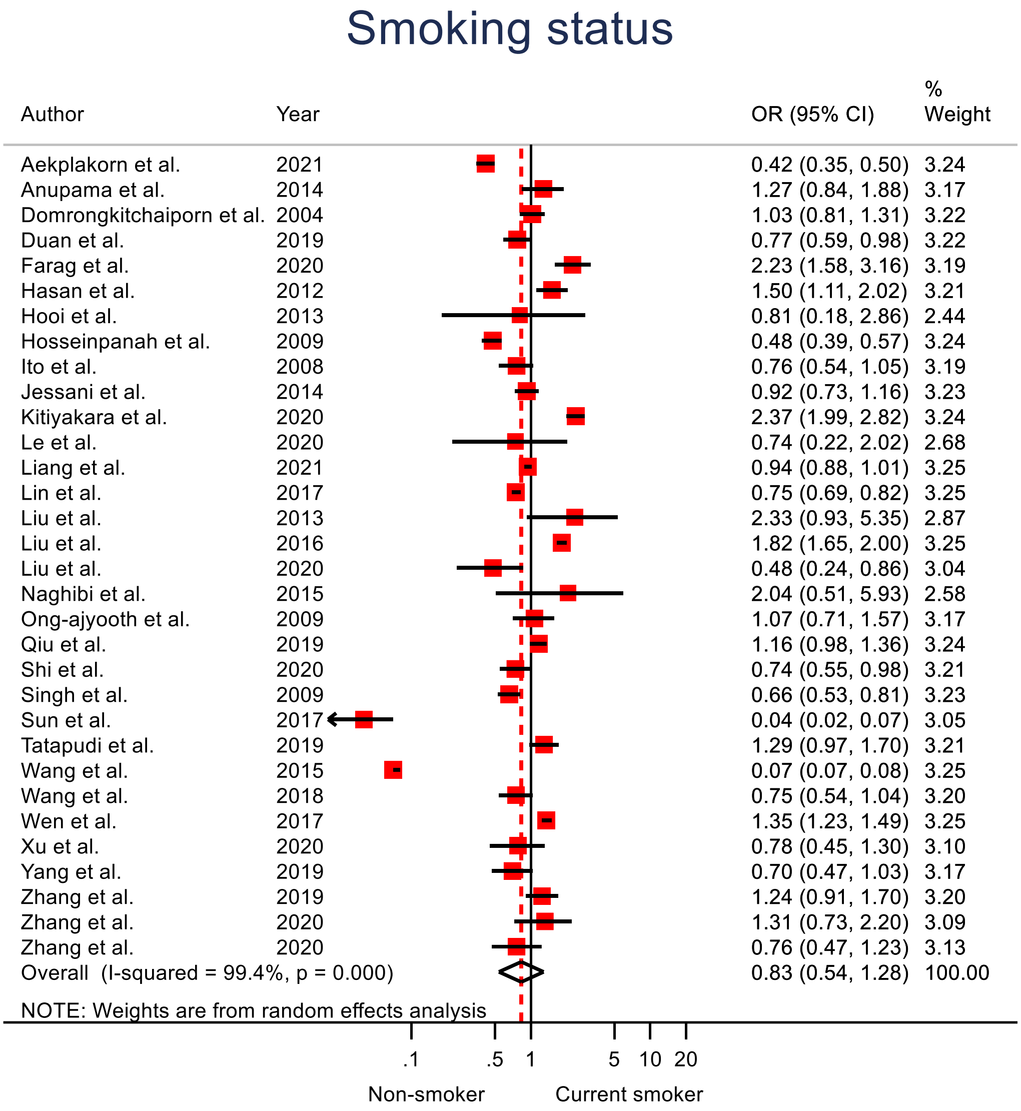

Fig S24. Meta-analysis of association between chronic kidney disease and alcohol consumption (current drinker or non-drinker).

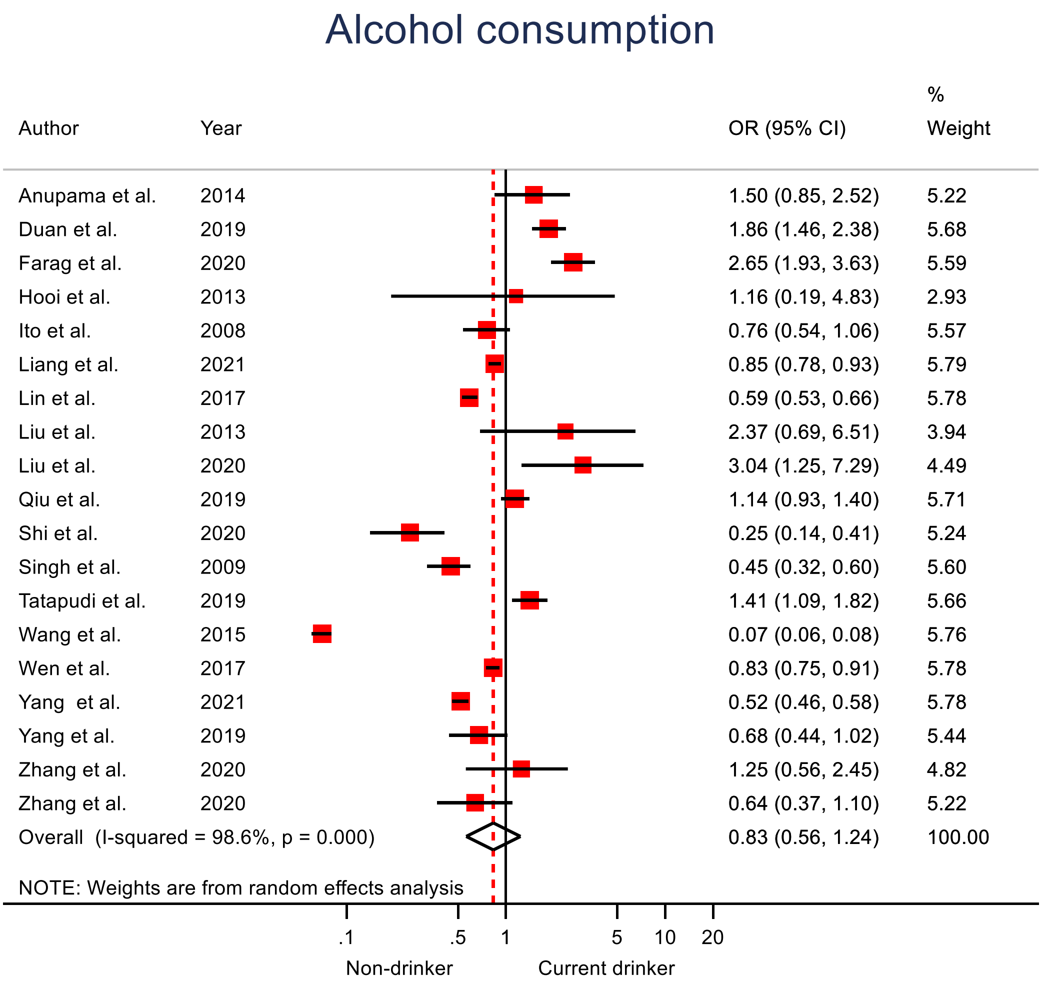

Fig S25. Meta-analysis of association between chronic kidney disease and family history of hypertension (yes or no).

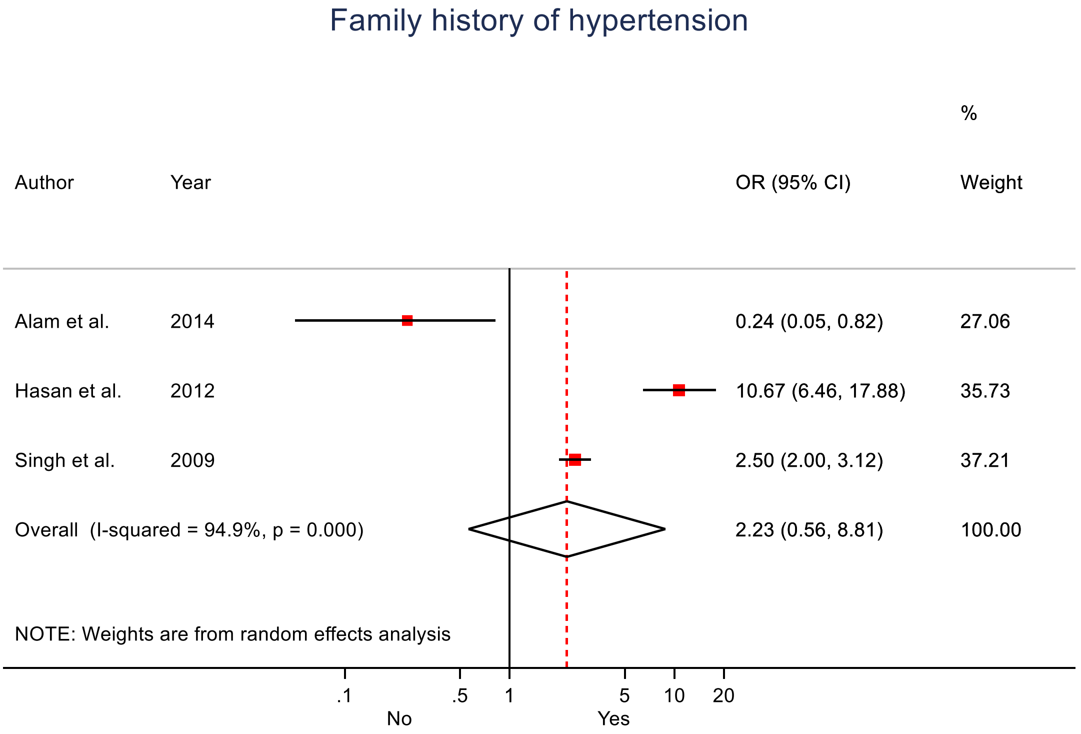

Fig S26. Meta-analysis of association between chronic kidney disease and family history of chronic kidney disease (yes or no).

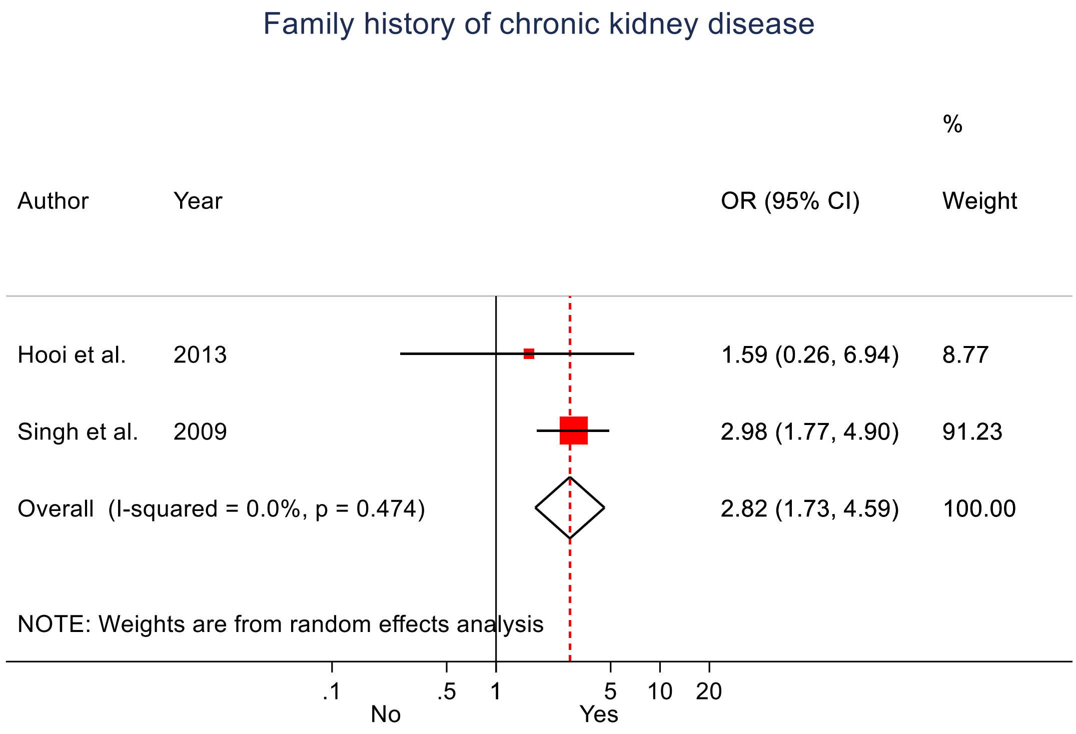

Fig S27. Meta-analysis of association between chronic kidney disease and physical activity (inactive or active).

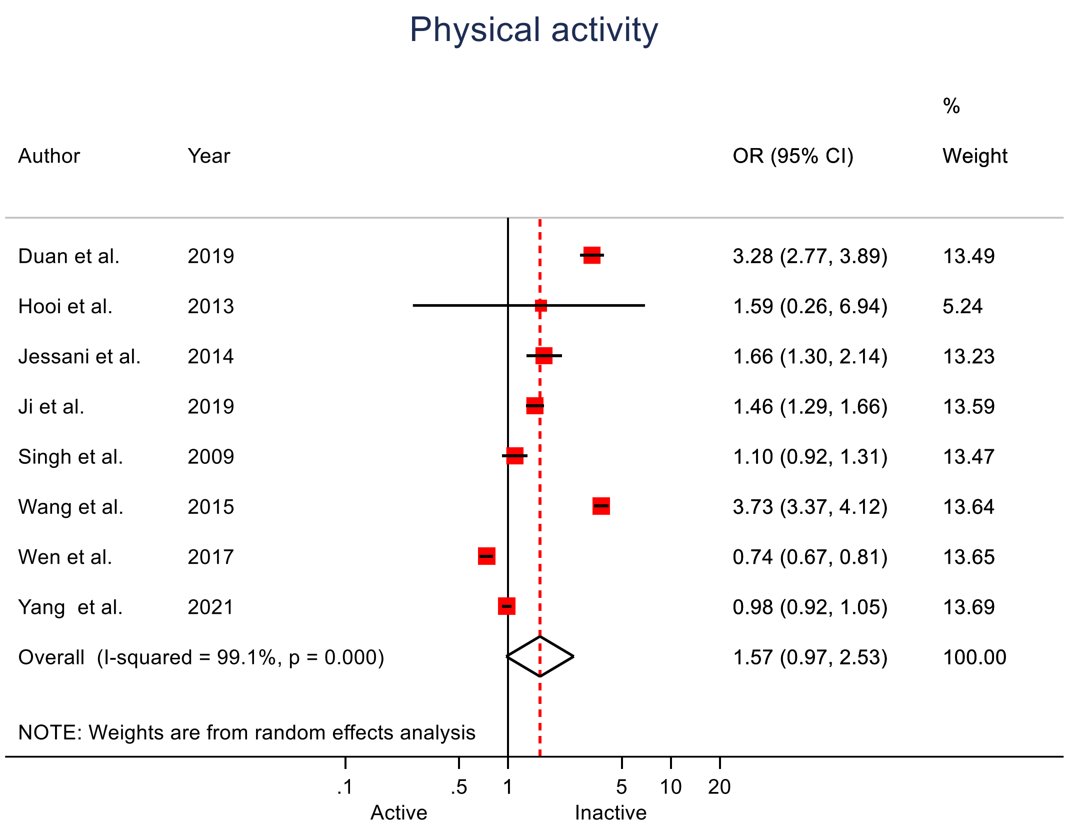

Fig S28. Meta-analysis of association between chronic kidney disease and nonsteroidal anti-inflammatory drugs: NSAIDs use (yes or no).

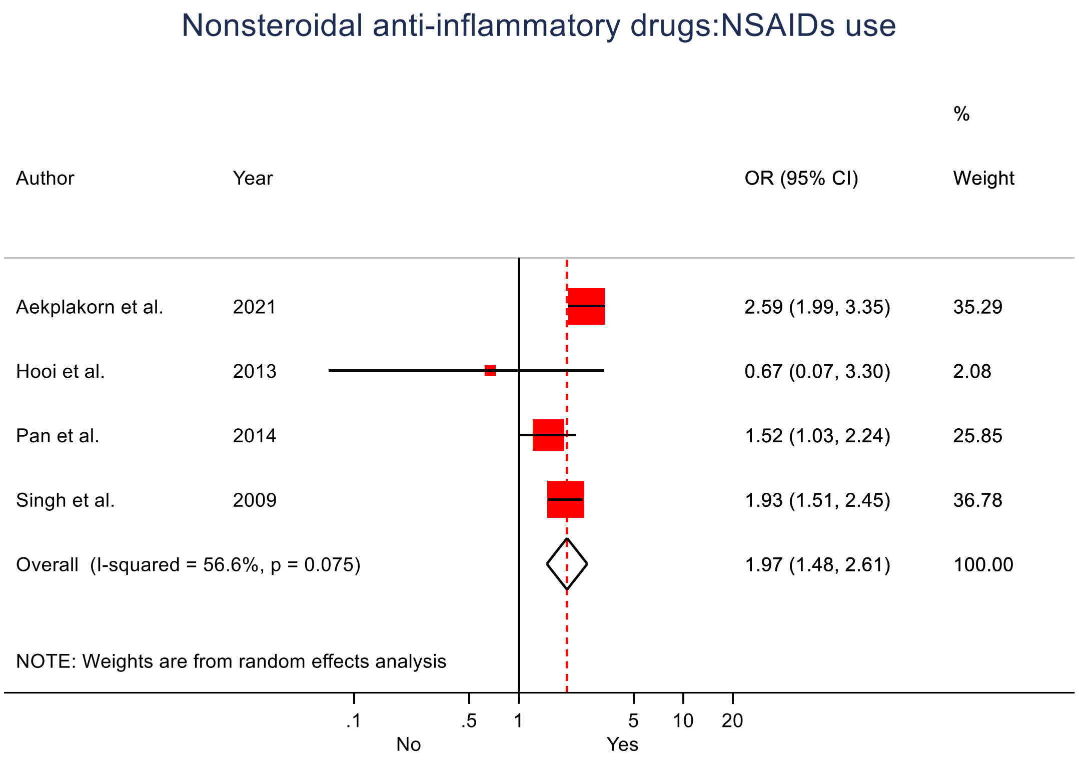

Fig S29. Meta-analysis of association between chronic kidney disease and CD4 cell count in HIV patients (≥200 or <200 cells/ml).

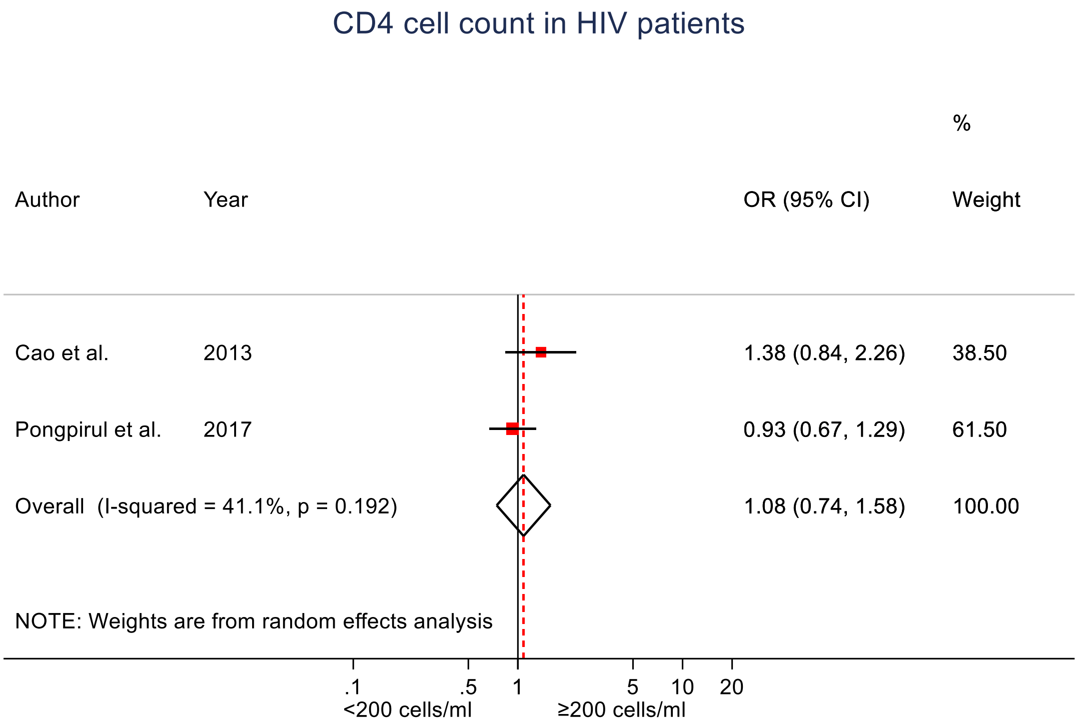

Supplement: S6 Appendix — (PDF) [file pone.0264393.s006.pdf]
