# Supplementary material for: Prevalence of chronic kidney disease stages 3–5 in low- and middle-income countries in Asia: A systematic review and meta-analysis
Source: PLoS One. 2022 Feb 25;17(2):e0264393. doi: 10.1371/journal.pone.0264393 (PMC8880400; doi:10.1371/journal.pone.0264393)
Supplement: S7 Appendix — (PDF) [file pone.0264393.s007.pdf]

S7 Appendix. Funnel plots, Egger' s test and trim-and-fill method in the pooled prevalence and associated risk factors of CKD stage 3-5 in LMICs in Asia.

Abbreviations:

CKD, chronic kidney disease; LMICs, low- and middle- income counties; LDLc, low-density lipoprotein cholesterol; HDLc, high-density lipoprotein cholesterol; HT, hypertension; HIV, human immunodeficiency viruses

Fig S30. Publication bias of included studies on the pooled prevalence of CKD stage 3-5 in LMICs in Asia.

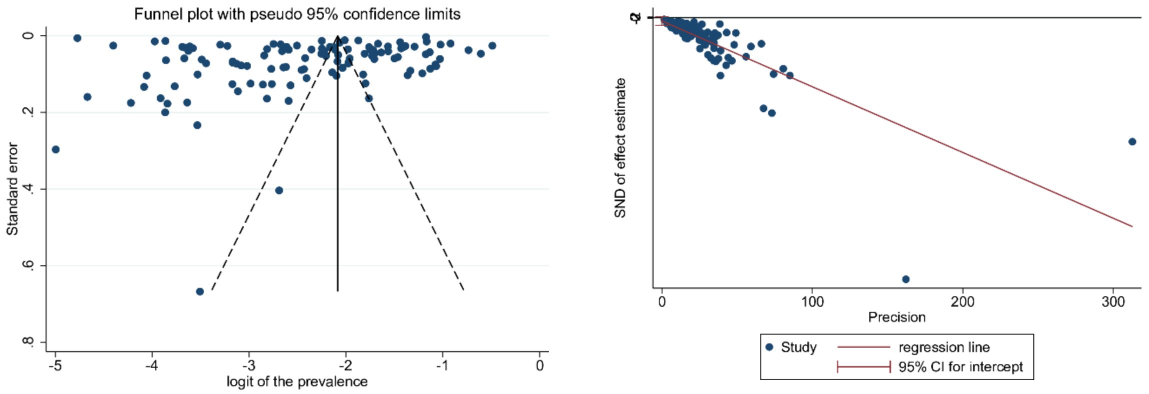

(Egger's test *P* = 0.137)

Fig S31. Publication bias of included studies on the association between elderly and CKD stage 3-5.

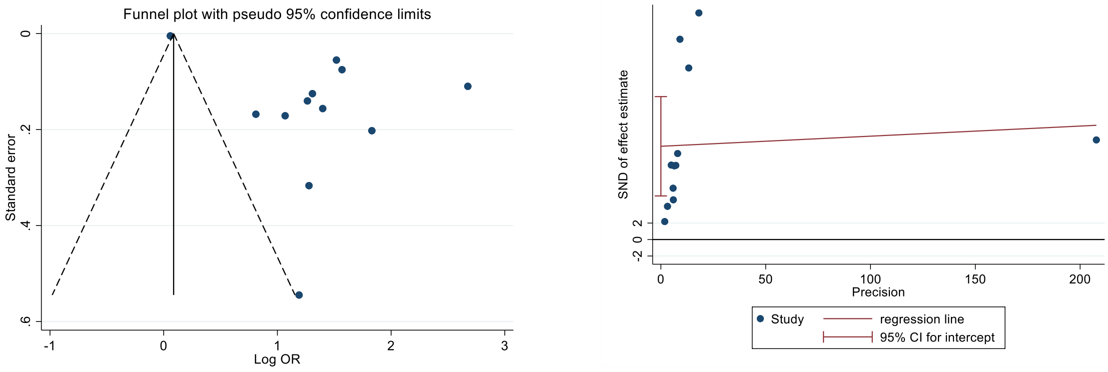

(Egger's test *P* = 0.002; trim-and-fill analysis = no trimming performed, data unchanged)

Fig S32. Publication bias of included studies on the association between gender (male) and CKD stage 3-5.

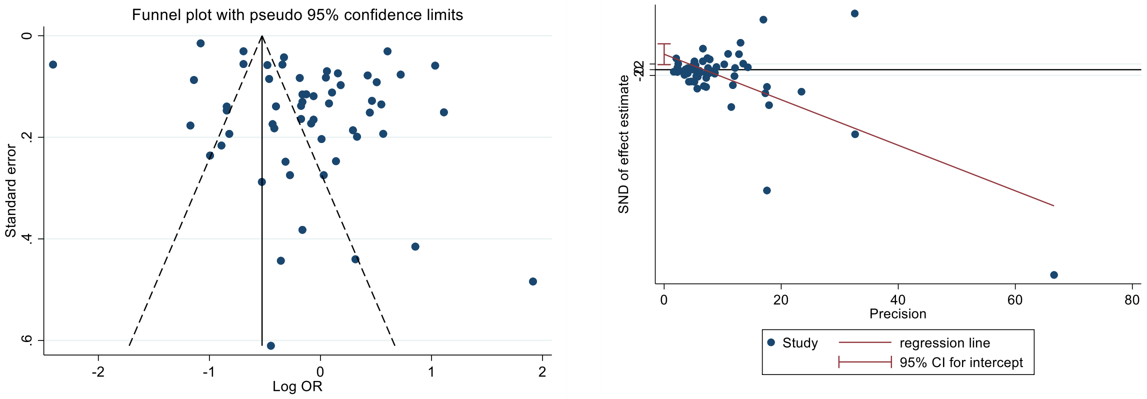

(Egger’s test P = 0.004; trim-and-fill analysis = no trimming performed, data unchanged)

Fig S33. Publication bias of included studies on the association between education and CKD stage 3-5.

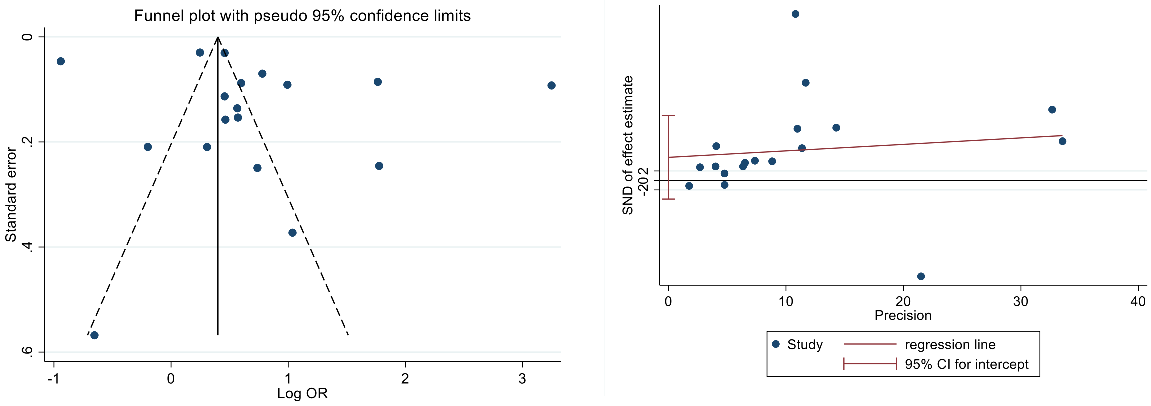

(Egger’s test P = 0.258)

Fig S34. Publication bias of included studies on the association between marital status and CKD stage 3-5.

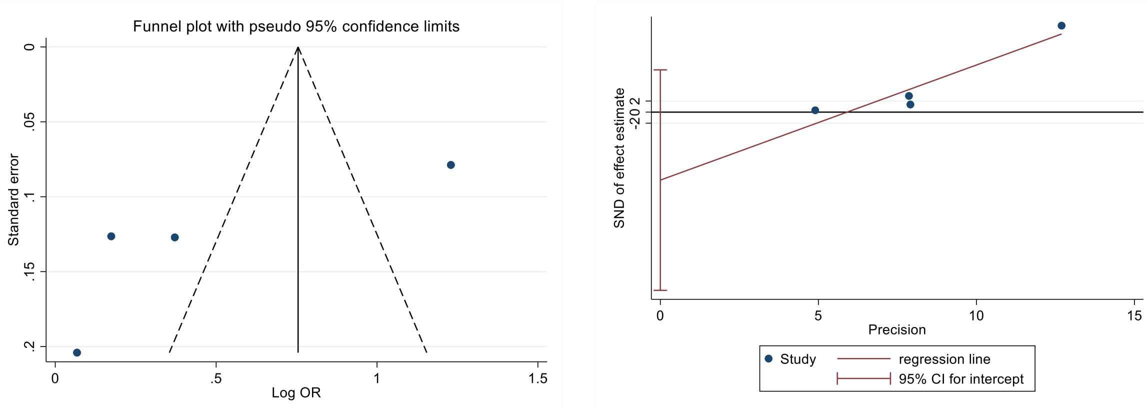

(Egger's test P = 0.118)

Fig S35. Publication bias of included studies on the association between obese and CKD stage 3-5.

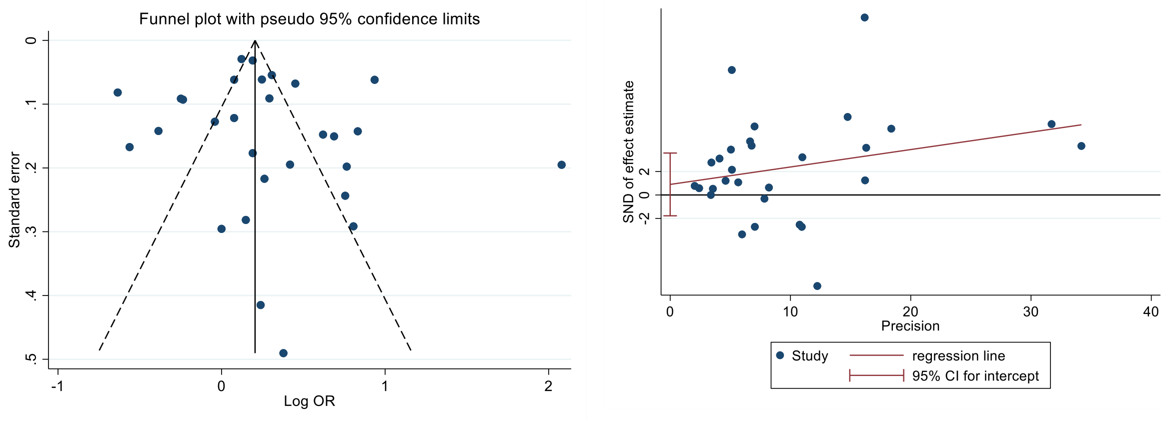

(Egger's test P = 0.496)

Fig S36. Publication bias of included studies on the association between lower weight and CKD stage 3-5.

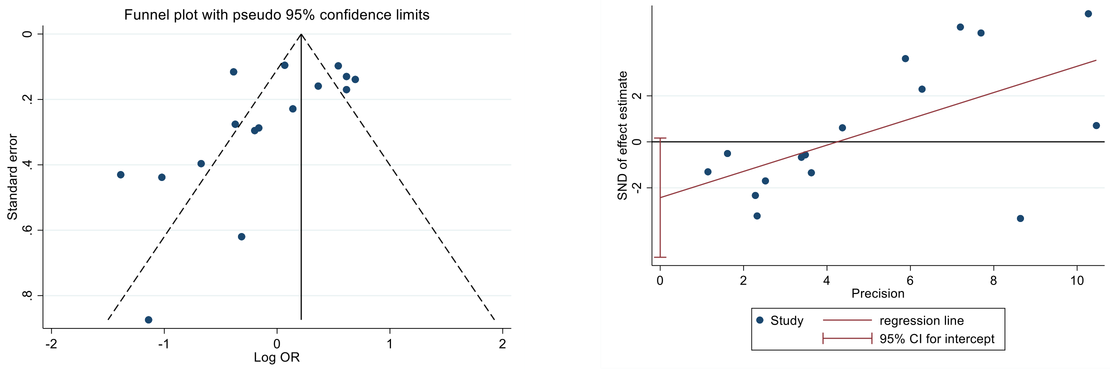

(Egger's test *P* = 0.064)

Fig S37. Publication bias of included studies on the association between hypertension and CKD stage 3-5.

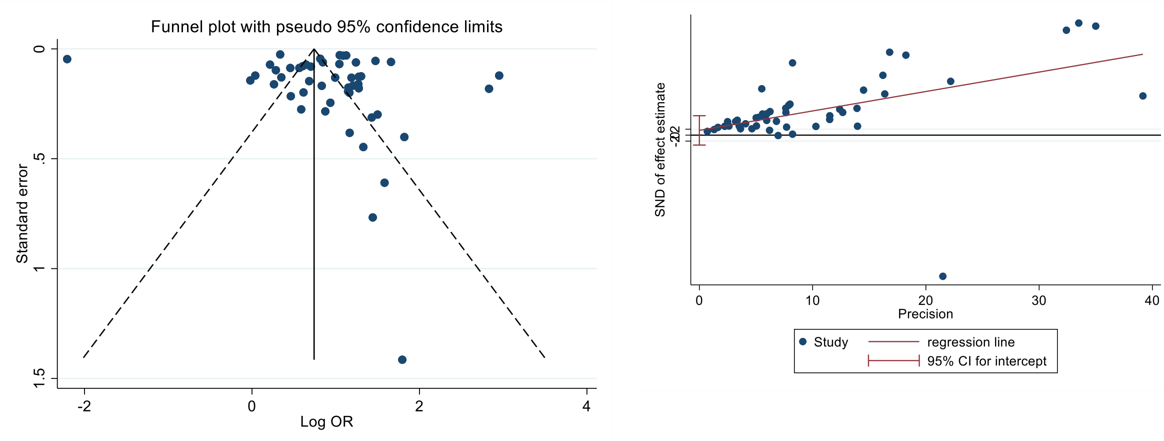

(Egger's test *P* = 0.518)

Fig S38. Publication bias of included studies on the association between diabetes and CKD stage 3-5.

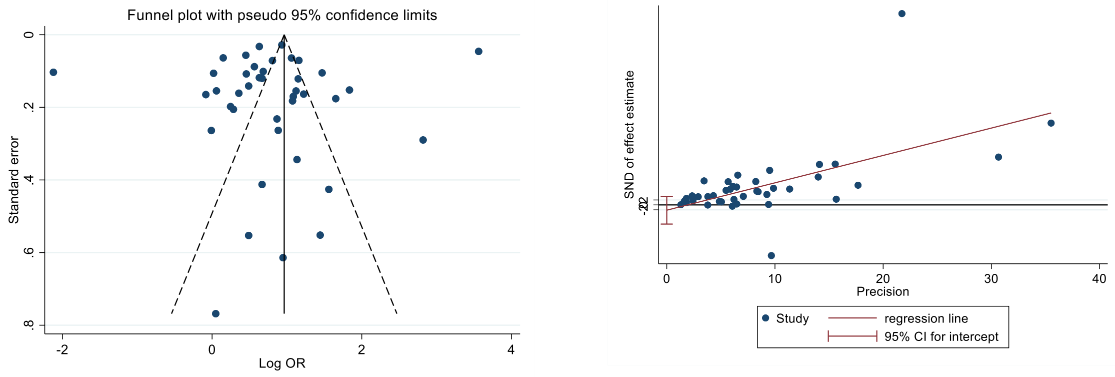

(Egger's test *P* = 0.446)

Fig S39. Publication bias of included studies on the association between dyslipidemia and CKD stage 3-5.

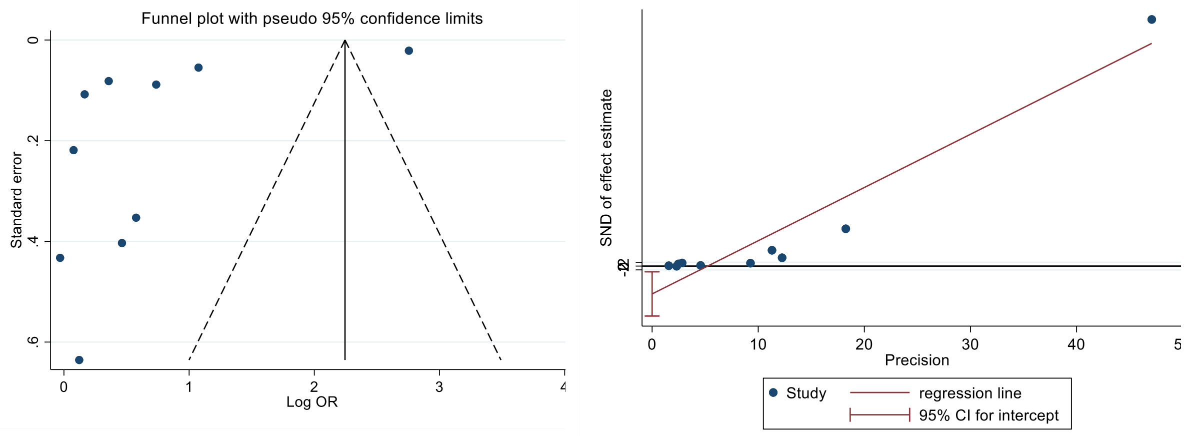

(Egger's test *P* = 0.020; trim-and-fill analysis = no trimming performed, data unchanged)

Fig S40. Publication bias of included studies on the association between hypertriglyceridemia and CKD stage 3-5.

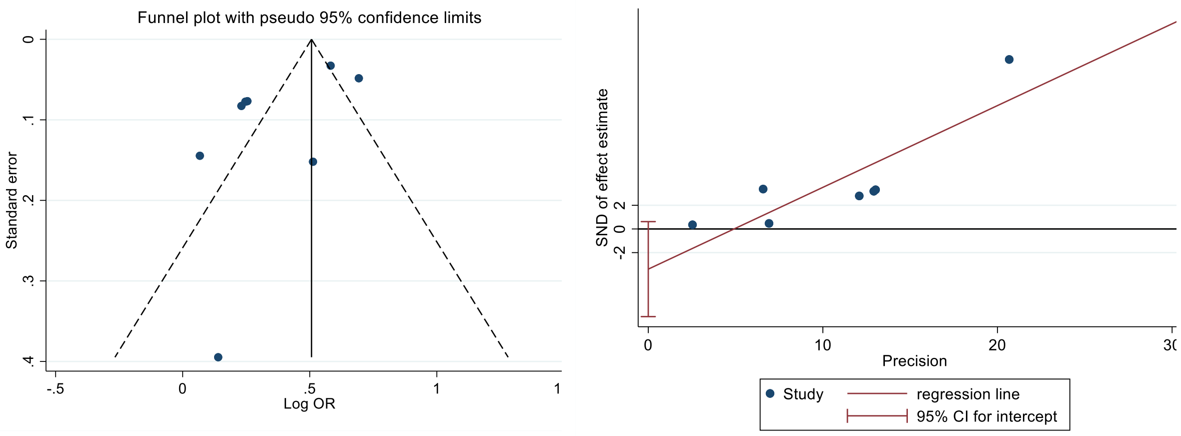

(Egger's test *P* = 0.084)

Fig S41. Publication bias of included studies on the association between Hypercholesterolemia and CKD stage 3-5.

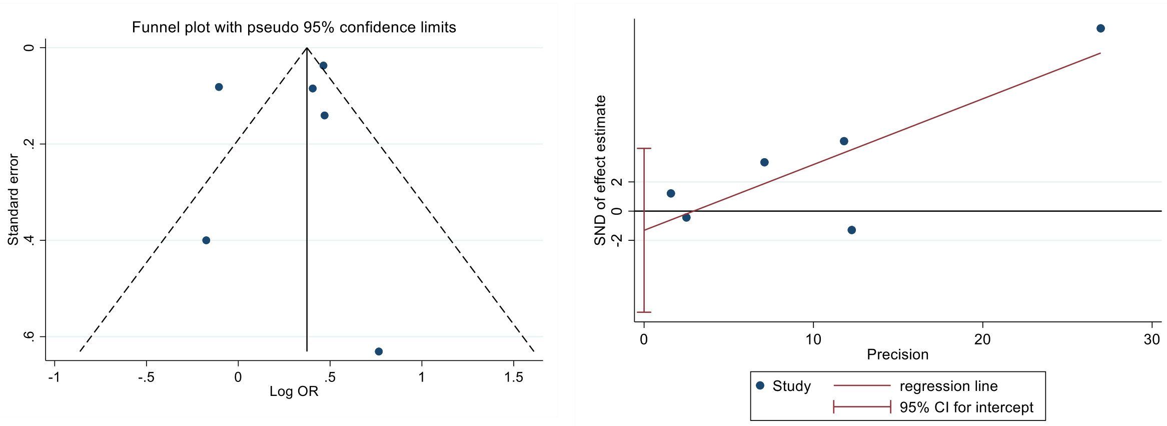

(Egger's test *P* = 0.551)

Fig S42. Publication bias of included studies on the association between high LDL cholesterol and CKD stage 3-5.

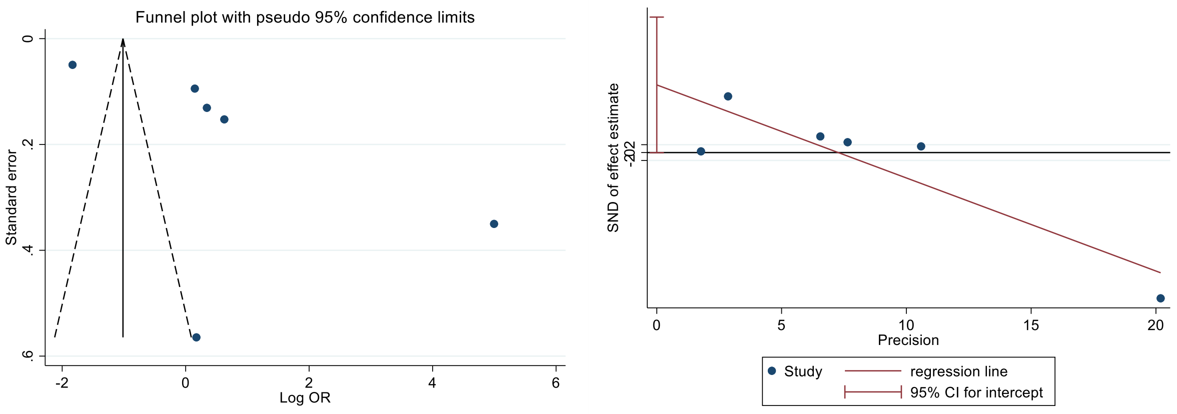

(Egger's test *P* = 0.050; trim-and-fill analysis = no trimming performed, data unchanged)

Fig S43. Publication bias of included studies on the association between low HDL cholesterol and CKD stage 3-5.

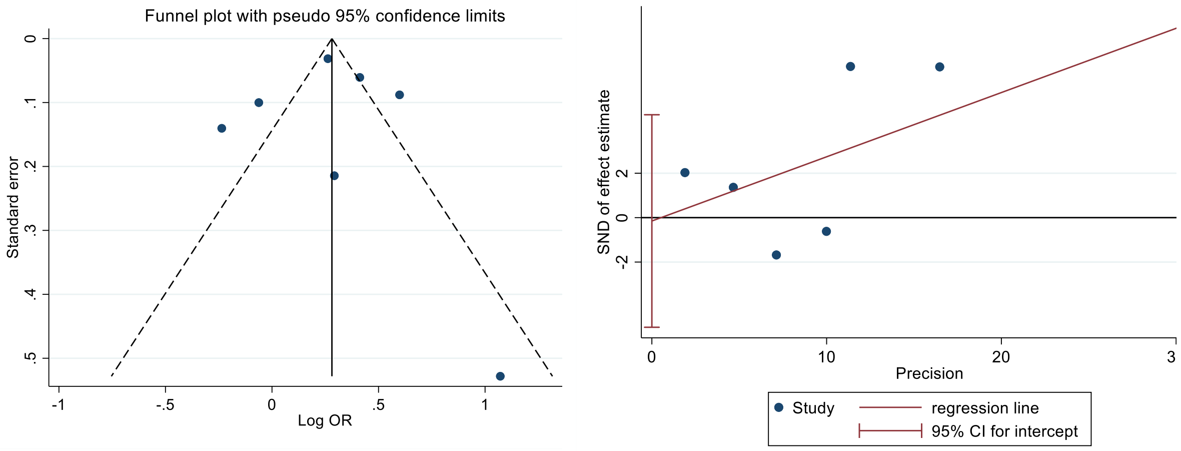

(Egger's test *P* = 0.938)

Fig S44. Publication bias of included studies on the association between hyperuricemia and CKD stage 3-5.

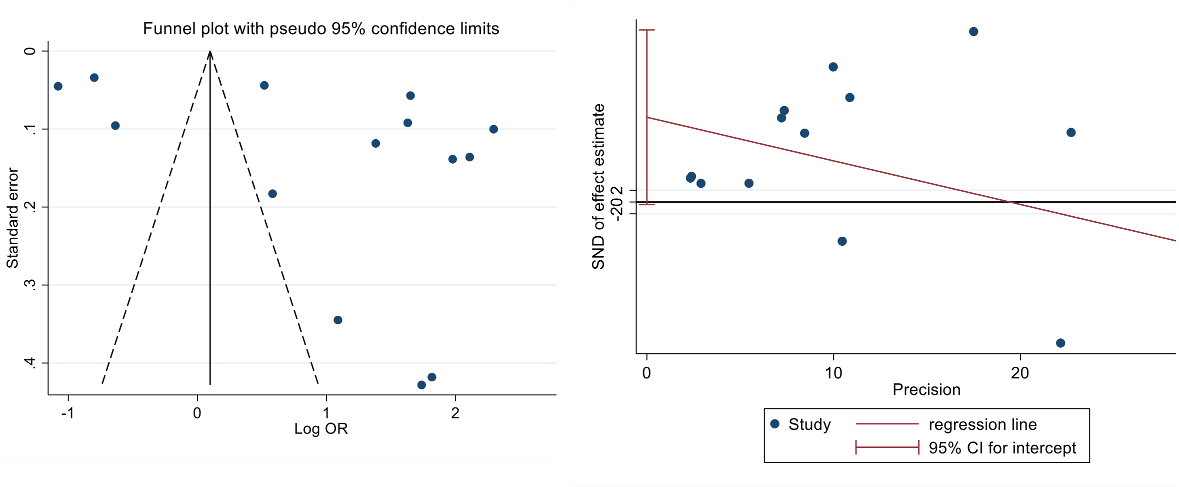

(Egger’s test P = 0.056)

Fig S45. Publication bias of included studies on the association between anemia and CKD stage 3-5.

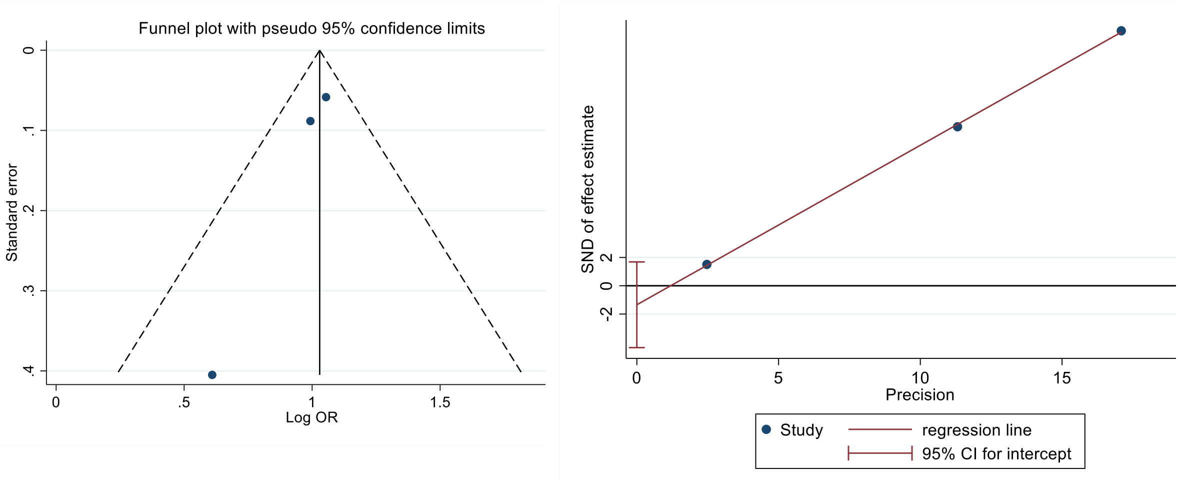

(Egger’s test P = 0.112)

Fig S46. Publication bias of included studies on the association between smoking status and CKD stage 3-5.

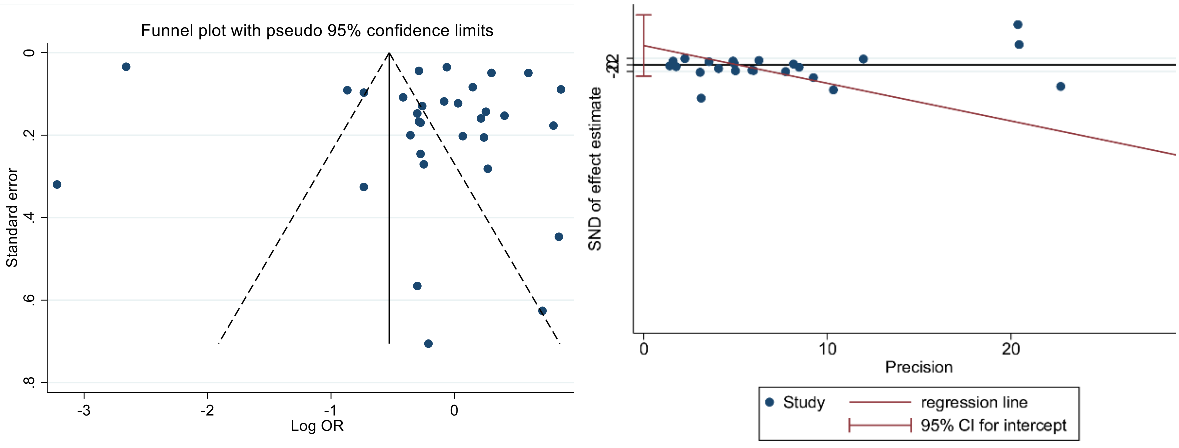

(Egger's test *P* = 0.199)

Fig S47. Publication bias of included studies on the association between alcohol consumption and CKD stage 3-5.

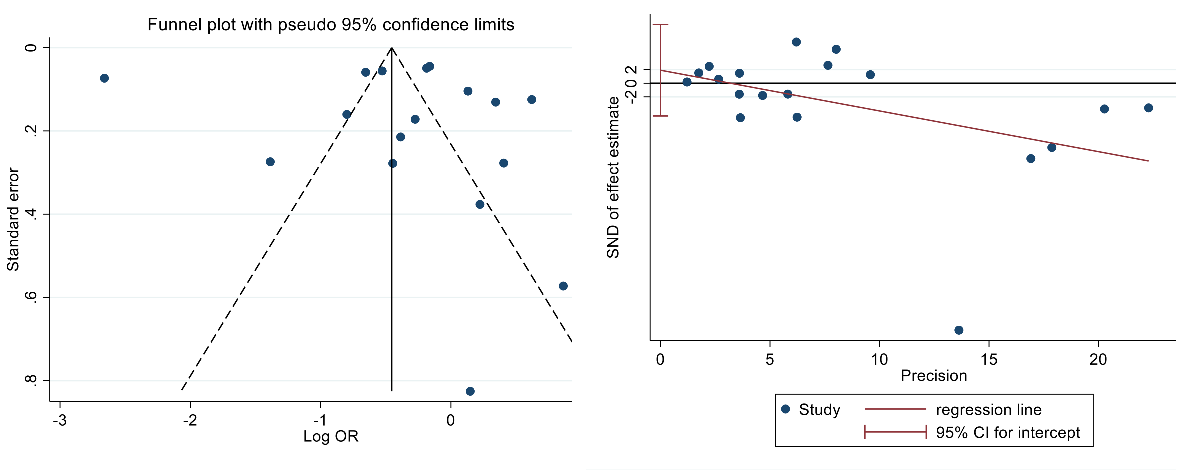

(Egger's test *P* = 0.557)

Fig S48. Publication bias of included studies on the association between history of coronary heart disease and CKD stage 3-5.

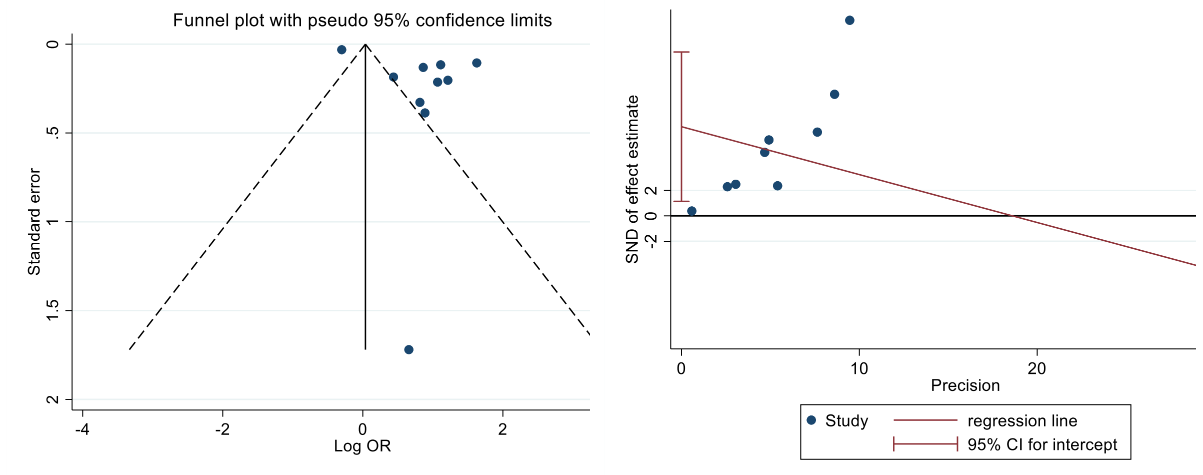

(Egger's test *P* = 0.025; trim-and-fill analysis, the recalculated OR with twelve adjusted studies was 1.99, 95% CI 1.18-3.35, *P =0.010*)

Fig S49. Publication bias of included studies on the association between history of stroke and CKD stage 3-5.

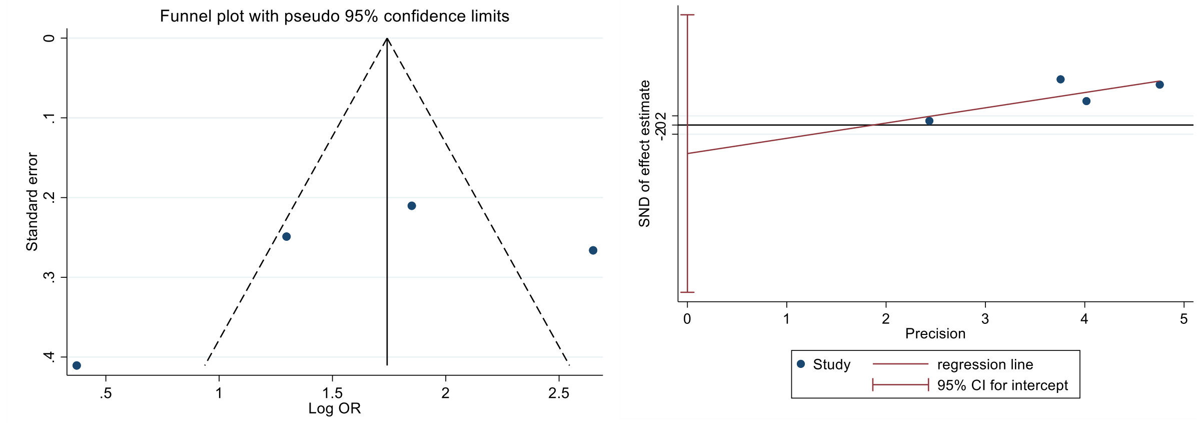

(Egger's test *P* = 0.470)

Fig S50. Publication bias of included studies on the association between history of cardiovascular disease and CKD stage 3-5.

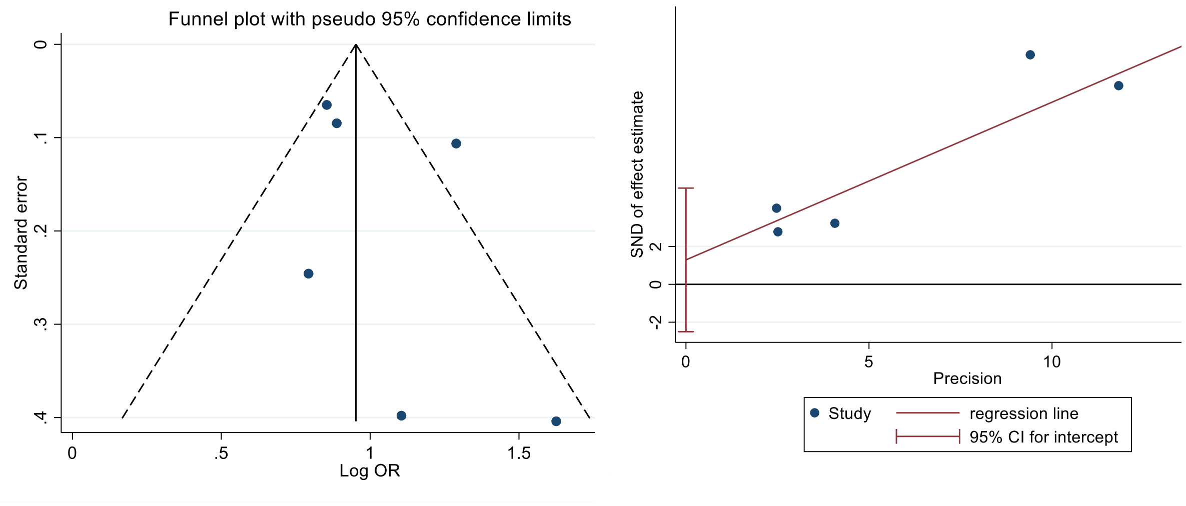

(Egger's test *P* = 0.398)

Fig S51. Publication bias of included studies on the association between family history of hypertension and CKD stage 3-5.

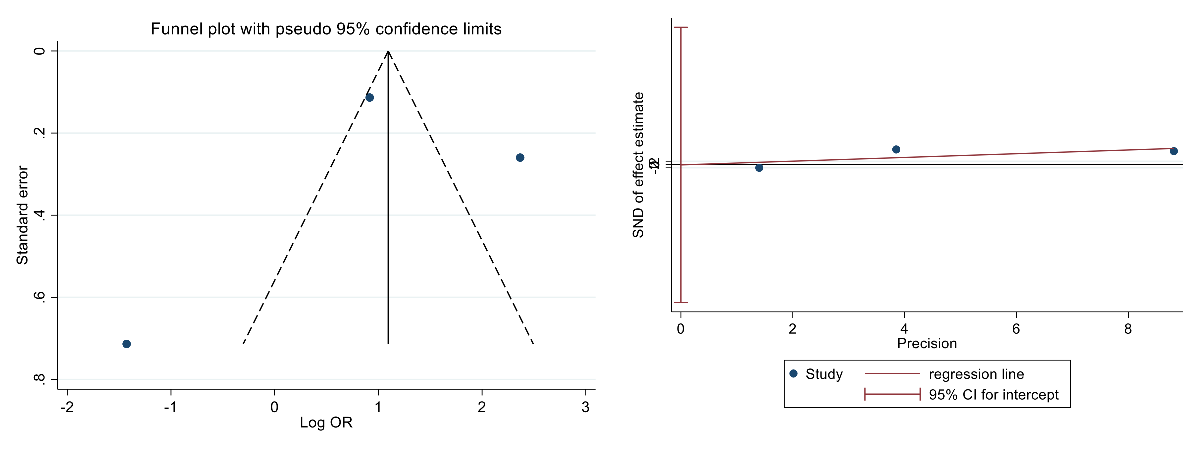

(Egger's test *P* =0.977)

Fig S52. Publication bias of included studies on the association between family history of chronic kidney disease and CKD stage 3-5.

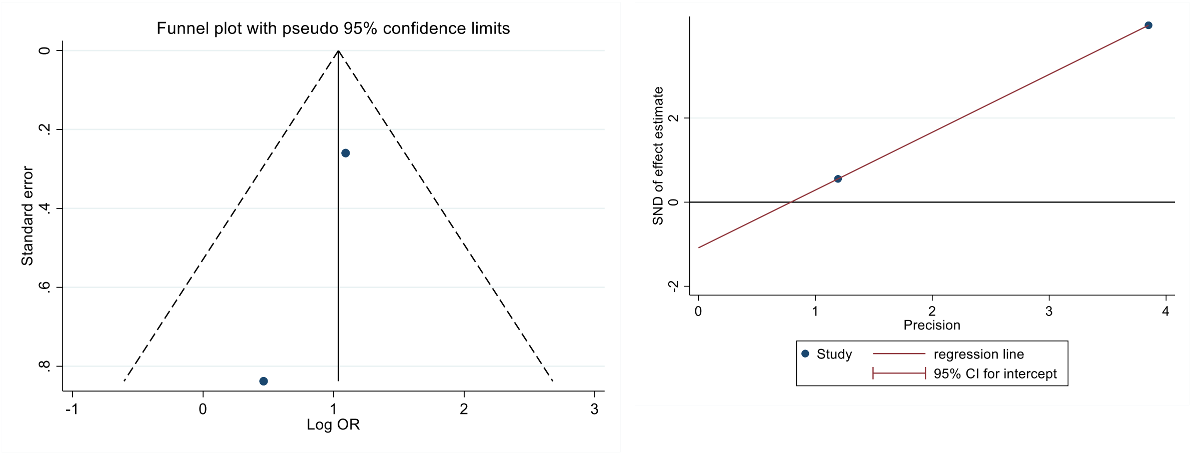

(Egger's test *P* = can't compute)

Fig S53. Publication bias of included studies on the association between physical activity and CKD stage 3-5.

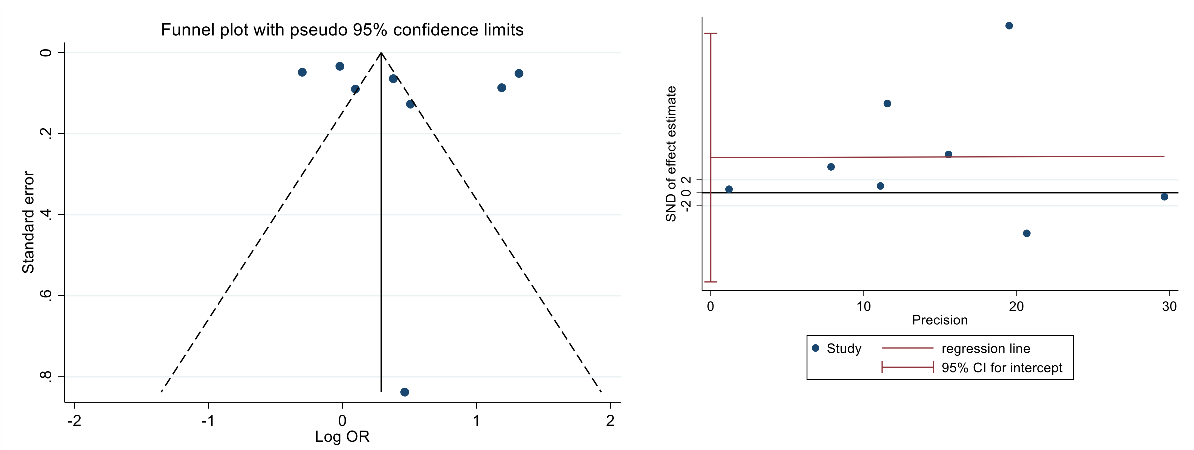

(Egger's test *P* = 0.514)

Fig S54. Publication bias of included studies on the association between nonsteroidal anti-inflammatory drugs: NSAIDs use and CKD stage 3-5.

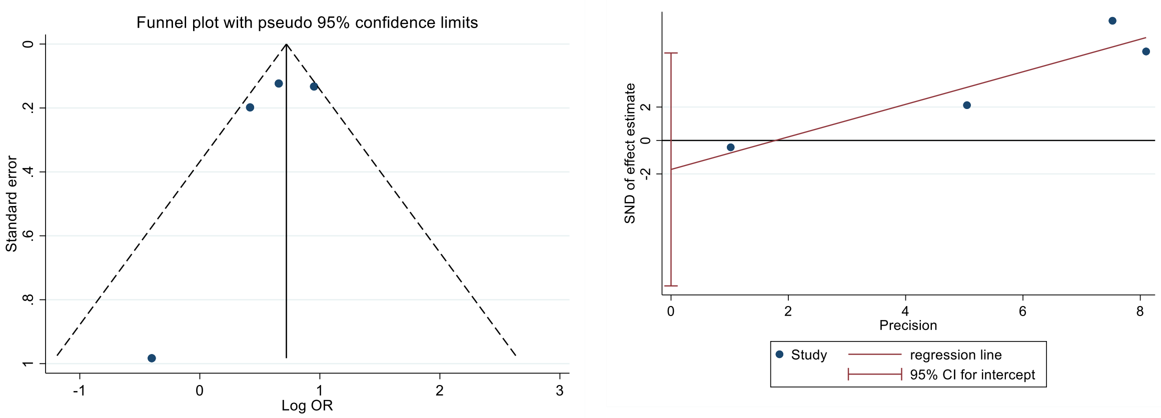

(Egger’s test P = 0.397)

Fig S55. Publication bias of included studies on the association between CD4 cell count in HIV patients and CKD stage 3-5.

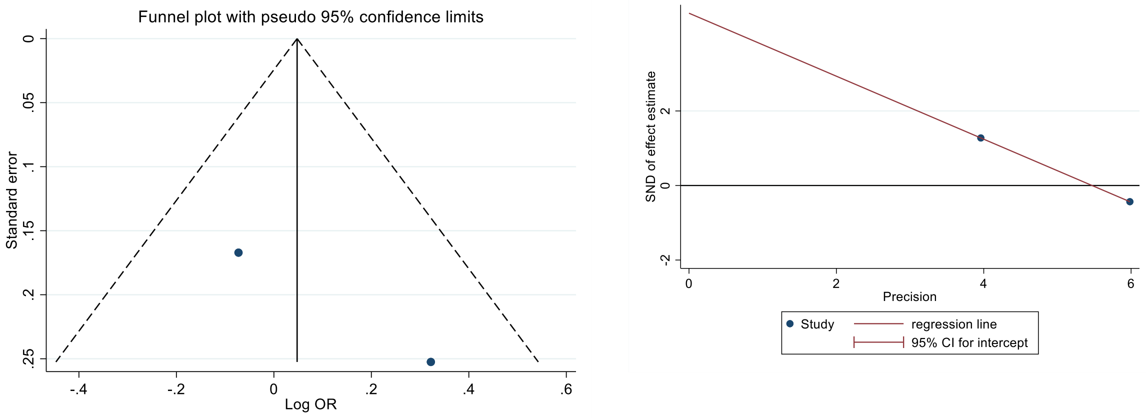

(Egger’s test P = can’t compute)
