# Supplementary material for: Prevalence of chronic kidney disease stages 3–5 in low- and middle-income countries in Asia: A systematic review and meta-analysis
Source: PLoS One. 2022 Feb 25;17(2):e0264393. doi: 10.1371/journal.pone.0264393 (PMC8880400; doi:10.1371/journal.pone.0264393)
Supplement: S8 Appendix — (PDF) [file pone.0264393.s008.pdf]

Citation

Pongpan Suriyong, Penkarn Kanjanarat, Chidchanok Ruengorn, Chairat Shayakul, Puree Anantachoti. Prevalence of Chronic Kidney Disease Stage 3-5 in Low- and Middle-income Countries in Asia: A Systematic Review and Meta-analysis. PROSPERO 2019 CRD42019120519 Available from: https://www.crd.york.ac.uk/prospero/display_record.php?ID=CRD42019120519

Review question [1 change]

What was the prevalence of CKD in low- and middle-income countries in Asia?

Searches [1 change]

We will search articles from the electronic databases published from inception until 30 th Nov, 2020, including PubMed/MEDLINE, ScienceDirect, EMBASE, Scopus, Cochrane, Thai Library Integrated System and Thai Thesis Database. The conference topics and gray literature sources would be interrogated. The literature search would be limited to the English or Thai language. The search strategies had been developed related to prevalence, chronic kidney disease, region and country economics in Asia.

Types of study to be included [2 changes]

We will include the published articles that:

1) They could be identified from the search from databases and unpublished literature in conferences and other sources by hand search.

2) They were descriptive studies and observation studies, i.e., cross-sectional and cohort studies.

3) They included both males and females.

4) They were included a population of at least 50 participants.

5) The study participants were adults aged 15 years and above.

6) Chronic kidney disease was estimated by serum creatinine (Scr) or estimated glomerular filtration rate (eGFR), using the CKD-EPI creatinine equation or 4-variable Modification of Diet in Renal Disease (MDRD) or body surface area (BSA) standardized Cockcroft-Gault (CG) equations.

7) They were available in English or in Thai language.

Condition or domain being studied [1 change]

Chronic kidney disease in adult populations aged 15 years and above who lived on the low- and middle-income countries in Asia. Moreover, chronic kidney disease was estimated by serum creatinine (Scr) or estimated glomerular filtration rate (eGFR), using the CKD-EPI creatinine equation or 4-variable Modification of Diet in Renal Disease (MDRD) or body surface area (BSA) standardized Cockcroft-Gault (CG) equations.

Participants/population [1 change]

Adult participants aged 15 years and above, lived in low- and middle-income countries in Asia (both genders in general population and high-risk populations).

Intervention(s), exposure(s) [1 change]

Eligibility criteria: We will include Descriptive studies and observation studies, i.e., cross-sectional and cohort studies reported prevalence of CKD in the population in low- and middle-income countries in Asia.

Exclusion criteria: We will exclude studies that (1) review articles, (2) not available in full-text version, (3) selected participants based on the absence or presence of kidney disease, (4) studied only in pregnant women, (5) studied only in children, and (6) duplicate publications.

Comparator(s)/control

None

Context [1 change]

Chronic kidney disease (CKD) is increased rapidly around the world and posts significant economic burden. Most people with CKD lives in low-income and middle- income countries (LMICs) and in Asia. The death rate from CKD was growing more than 5% yearly. Recently, there are 2 review studies of epidemiology of CKD specific to LMICs and to region, first study is a systematic review and meta-analysis of the relevant evidence on CKD prevalence in the African, and another study describes the prevalence of CKD in LMICs. However, there is little understanding of epidemiology of CKD in LMICs in Asia. Based on our review until present, there is no meta-analysis study of the prevalence of CKD and associated risk factors of CKD in LMICs in Asia. Hence, the researchers aim to find the pool of prevalence of CKD in LMICsin Asia.

Main outcome(s) [2 changes]

Prevalence of CKD in populations in LMICs in Asia. We will include studies that estimated chronic kidney disease by serum creatinine (Scr) or estimated glomerular filtration rate (eGFR), using the CKD-EPI creatinine equation or 4-variable Modification of Diet in Renal Disease (MDRD) or body surface area (BSA) standardized Cockcroft-Gault (CG) equations.

Measures of effect

Prevalence and odds ratios/relative risks

Additional outcome(s) [1 change]

None

Measures of effect

Not applicable

Data extraction (selection and coding)

Data details from each study will be extracted and inputted in a standard data collection form. Data extraction was independently performed by two investigators. Any dissimilarities in data extraction will be discussed until reaching a consensus. We would include the data details as follows: title of the article, first author's surname, authors' affiliation, publication year, study design, location where the study was conducted, study population, sample size, inclusion criteria, exclusion criteria, risk factors and diagnosis methods of CKD, number of cases, number of controls, percentage of male and female, potential confounders, and the study outcomes. Subsequently, we would extract prevalence of CKD, odds ratios (OR) or relative risk (RR), and adjusted OR if available.

Risk of bias (quality) assessment [1 change]

The included studies will be evaluated their quality by two investigators. We would use the Newcastle-Ottawa quality assessment scale (NOS) to assess bias in each study. If there was any inconsistent assessment, we would resolve through discussion to create consensus assessment in each study. This study will include descriptive, cross-sectional and cohort studies and categorize each component of the study as containing high, low or unclear risk of bias. We will use each bias-reducing standard to award stars of NOS, which consist of 8 multiple-choice questions, to assess quality of each non-randomized study in three areas, including 1) the selection of the study participants (maximum of 4 stars), 2) the comparability of groups by adjusting for first and second most appropriate factors (maximum of 2 stars), and 3) the ascertainment of the

interested outcome in cohort studies (maximum of 3 stars). A total score of <3 is considered poor, 4–6 moderate, and 7–9 high quality. We will select moderate to high quality studies to test sensitivity analysis.

We will control the validity of the systematic reviews and publication bias by using objective judgement funnel and Egger's test.

Strategy for data synthesis

We will analyze data using fixed effect model or random-effects model to perform pooled estimates of prevalence, RR, ORs, weighted mean difference (WMD) and 95% confidence interval (95%CI). Heterogeneity will be assessed using Cochran Q, I^2 and ?² statistic, and a p-value <0.05 which was considered statistically significant. If summary estimates with a conforming I^2 < or = 50%, we will calculate pooled estimates using a fixed-effects model. If Cochran's Q test quantified the proportion of the total variation across studies rather than expected variance (df) and summary estimated with a conforming I^2 > 50% we will calculate pooled estimates using a random-effects model.

Analysis of subgroups or subsets [1 change]

We will perform the pre-planned subgroup analysis to repeat the random effects of meta-analysis after the addition of a low-quality study on the pooled estimate of prevalence of CKD.

Contact details for further information

Pongpan Suriyong
pp_suriyong1@hotmail.com

Organisational affiliation of the review

Faculty of Pharmacy, Chiang Mai University, Chiang Mai, Thailand.
www.pharmacy.cmu.ac.th

Review team members and their organisational affiliations [1 change]

Mr Pongpan Suriyong. Faculty of Pharmacy, Chiang Mai University, Chiang Mai, Thailand.
Dr Penkarn Kanjanarat. Department of Pharmaceutical Care, Faculty of Pharmacy, Chiang Mai University, Chaing Mai, Thailand.
Dr Chidchanok Ruengorn. Department of Pharmaceutical Care, Faculty of Pharmacy, Chiang Mai University, Chaing Mai, Thailand.
Professor Chairat Shayakul. Renal Division, Department of Medicine, Faculty of Medicine Siriraj Hospital, Mahidol University, Bangkok, Thailand.
Assistant/Associate Professor Puree Anantachoti. Social and Administrative Pharmacy Department, Faculty of Pharmaceutical Science, Chulalongkorn University, Bangkok, Thailand.

Type and method of review

Epidemiologic, Meta-analysis, Systematic review

Anticipated or actual start date

01 October 2018

Anticipated completion date

30 April 2019

Funding sources/sponsors [1 change]

Graduate School, Chiang Mai University. Division of Research Administration, Academic Services and International Relations, Faculty of Pharmacy, Chiang Mai University

Conflicts of interest

Language

English

Country [1 change]

Thailand

Stage of review
Review Ongoing

Subject index terms status
Subject indexing assigned by CRD

Subject index terms
Asia; Humans; Income; Prevalence; Renal Insufficiency, Chronic; Risk Factors

Date of registration in PROSPERO
12 April 2019

Date of first submission
16 March 2019

Stage of review at time of this submission

**Stage**	**Started**	**Completed**
Preliminary searches	Yes	Yes
Piloting of the study selection process	Yes	Yes
Formal screening of search results against eligibility criteria	Yes	No
Data extraction	No	No
Risk of bias (quality) assessment	No	No
Data analysis	No	No

Revision note
Adjusting for the specific objective.

The record owner confirms that the information they have supplied for this submission is accurate and complete and they understand that deliberate provision of inaccurate information or omission of data may be construed as scientific misconduct.
The record owner confirms that they will update the status of the review when it is completed and will add publication details in due course.

Versions
12 April 2019
18 August 2020
22 November 2021
